# Supplementary material for: PCYT1A Regulates Phosphatidylcholine Homeostasis from the Inner Nuclear Membrane in Response to Membrane Stored Curvature Elastic Stress
Source: Dev Cell. 2018 May 21;45(4):481–495.e8. doi: 10.1016/j.devcel.2018.04.012 (PMC5971203; doi:10.1016/j.devcel.2018.04.012)
Supplement: Document S1. Figures S1–S7 [file mmc1.pdf]

Developmental Cell, Volume *45*

Supplemental Information

PCYT1A Regulates Phosphatidylcholine Homeostasis from the Inner Nuclear Membrane in Response to Membrane Stored Curvature Elastic Stress

Afreen Haider, Yu-Chen Wei, Koini Lim, Antonio D. Barbosa, Che-Hsiung Liu, Ursula Weber, Marek Mlodzik, Kadri Oras, Simon Collier, M. Mahmood Hussain, Liang Dong, Satish Patel, Anna Alvarez-Guaita, Vladimir Saudek, Benjamin J. Jenkins, Albert Koulman, Marcus K. Dymond, Roger C. Hardie, Symeon Siniossoglou, and David B. Savage

SUPPLEMENTAL INFORMATION

Fig. S1

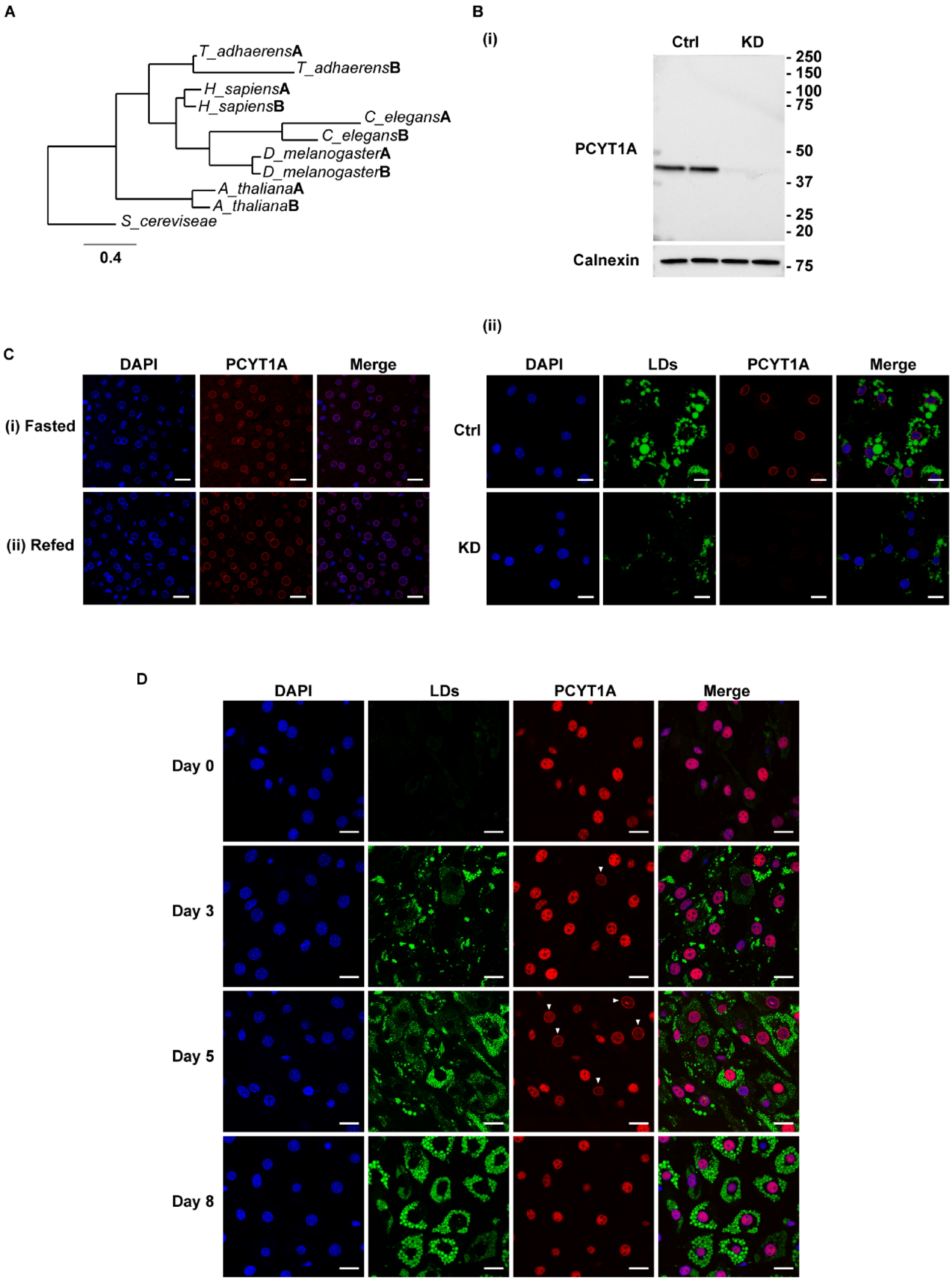

Figure S1. Related to figure 1. PCYT1A nuclear localization in WT mouse hepatocytes and differentiating OP9 adipocytes.

(A) CCT phylogenetic tree based on the alignment of sequences comprising the catalytic and M-domain of CCT from selected Metazoan, fungal and plant representatives. UniProt sequence IDs from top to bottom: B3RI62, B3RI63, P49585, Q9Y5K3, Q8IU09, P49583, Q7K4C7, Q9W0D9, Q9ZV56, F4JJE0, P13259; Labels A and B distinguish the paralogues in one species. Alignments were carried out with PROMALS (http://prodata.swmed.edu/promals/promals.php) and the tree was calculated with PhyMl algorithm with aLTR test as implemented in Phylogeny.fr package (http://www.phylogeny.fr). The branch length is proportional to the number of substitutions per site.

(B)Verification of the specificity of a PCYT1A primary antibody.

(i) PCYT1A immunoblotting using lysates obtained from mouse OP9 pre-adipocytes transfected with 30 nM non-targeting negative control (Ctrl) or *PCYT1A* specific siRNA (KD); Calnexin is used as a loading control. (ii) Immunostaining of PCYT1A in mouse OP9 adipocytes at day 2 of differentiation (induction with insulin & oleic acid as described in Methods). PCYT1A shows nuclear localization in cells treated with negative control siRNA (Ctrl), while it is almost undetectable in cells treated with *PCYT1A* specific siRNA (KD). BODIPY (green) was used to stain lipid droplets (LDs) as described in Methods.

(C) PCYT1A localizes to the nuclear membrane of hepatocytes from 10-week old WT mice (i) fasted for 16 h or (ii) fasted for 16 h and refed for 4 h.

(D) During adipogenic differentiation induced by hormonal mixture (dexamethasone, IBMX and insulin) in OP9 cells, PCYT1A is found in the intranuclear region in pre-adipocytes and transiently translocates to nuclear membrane (cells marked with white arrow heads) between days 3 and 5 of differentiation. BODIPY (green) was used to stain lipid droplets (LDs) as described in Methods.

Scale bars, 20 μm.

Fig. S2

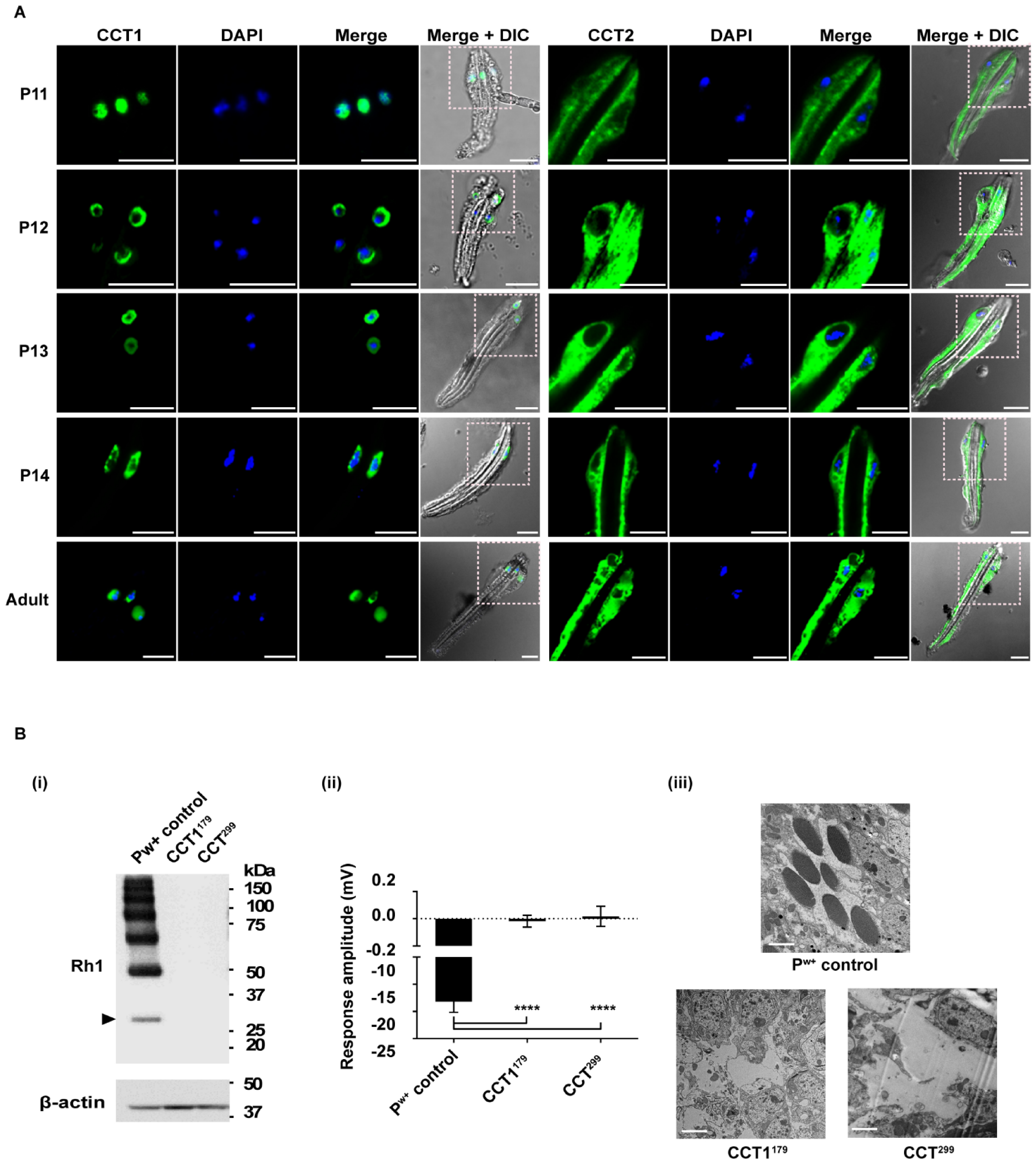
A
CCT1
DAPI
Merge
Merge + DIC
CCT2
DAPI
Merge
Merge + DIC
P11
P12
P13
P14
Adult
B
(i)
Pw+ control
CCT1179
CCT299
kDa
150
100
75
50
37
25
20
Rh1
β-actin
50
37
(ii)
Response amplitude (mV)
0.2
0.0
-0.2
-10
-15
-20
-25
****
****
Pw+ control
CCT1179
CCT299
(iii)
Pw+ control
CCT1179
CCT299

Figure S2. Related to figure 2. CCT1 is associated with the nuclear membrane in photoreceptors during pupal development.

(A) Individual ommatidia from different pupal (P11-14) and adult stages of transgenic flies expressing N-terminal GFP-tagged CCT1 or CCT2 were dissociated, stained with DAPI and imaged as described in methods. Merged fluorescent channels are shown with the DIC images (merge + DIC) for context. All panels are zoomed in regions from the boxed region in the 'merge + DIC' panel. Scale bars, 10 µm.

(B) Eye-specific GMR-hid *CCT* null flies, CCT^{179} (*CCT1*Δ) and CCT^{299} (*CCT1*Δ and *CCT2*Δ) are blind and have defective rhabdomere development (i) Rhodopsin expression is essentially undetectable in flies with *CCT* null eyes compared to the wild-type control (P^{w+}) as seen in immunoblots probed with an Rh1-antibody. (ii) Response amplitude generated in ERG recordings at maximal intensity of light was negligible in *CCT* null eyes. Data are mean ± SD from 4-5 flies of each genotype. One-way ANOVA with Bonferroni multiple comparisons ****, $p<0.0001$. (iii)Transmission electron micrographs of retinal sections from the wild-type flies (P^{w+} control) and flies with *CCT* null eyes ($CCT1^{179}$ and CCT^{299}). Scale bars, 2µm.

Fig. S3

A

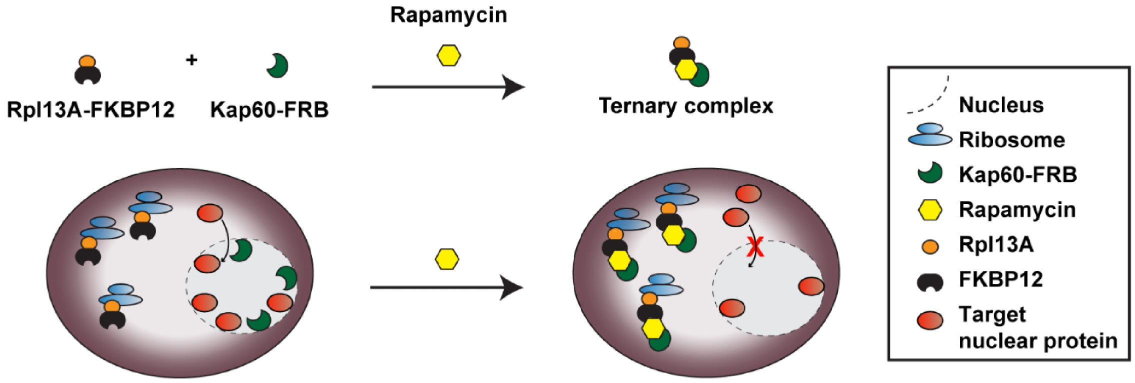

Rapamycin
Rpl13A-FKBP12
Kap60-FRB
Ternary complex
Nucleus
Ribosome
Kap60-FRB
Rapamycin
Rpl13A
FKBP12
Target nuclear protein

B

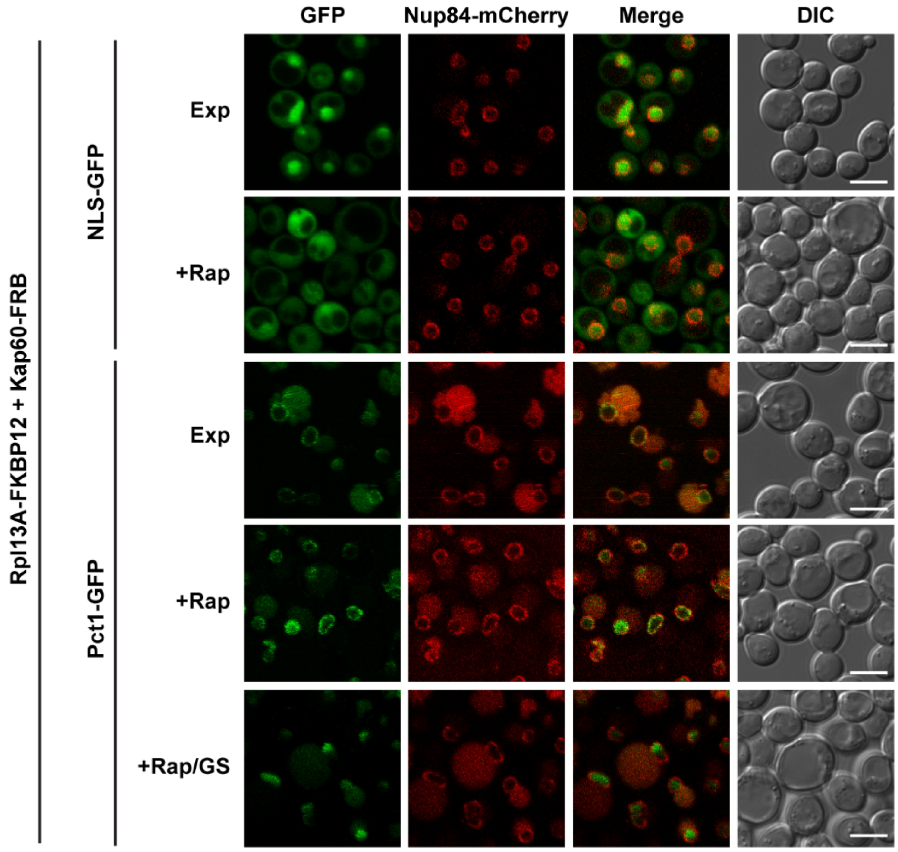

GFP
Nup84-mCherry
Merge
DIC
Exp
+Rap
Exp
+Rap
+Rap/GS
NLS-GFP
Pct1-GFP
Rpl13A-FKBP12 + Kap60-FRB

Figure S3. Related to figure 3. Pct1 associates with the inner nuclear membrane.

(A) Schematic of the anchor-away technique (adapted from Haruki *et al*., 2008). Rapamycin promotes tethering of the karyopherin Kap60 (Kap60-FRB) to the ribosomal protein Rpl13A (Rpl13A-FKBP12), due to heterodimerization of FKBP12 and FRB, causing depletion of Kap60 from the nucleus and would block the nuclear import of target proteins.

(B) Anchor-away strain expressing Rpl13A-2xFKBP12, Kap60-FRB, Nup84-mCherry (nuclear membrane marker) and Pct1-GFP was grown to the exponential phase (Exp) and treated with rapamycin (+Rap) for 15 min. Glucose starvation (+Rap/GS) was induced after the initial rapamycin treatment by transferring the cells into rapamycin-medium lacking glucose for 30 min. To confirm that depletion of Kap60 prevents nuclear import, GFP fused to a known nuclear localisation signal (NLS-GFP) was used as a positive control. Cells were imaged as described in Methods. Scale bars, 5 µm. NLS-GFP concentrates in the nucleus in untreated cells but it is increasingly redistributed to the cytosol following Kap60 inactivation demonstrating that nuclear import is successfully blocked.

Fig. S4

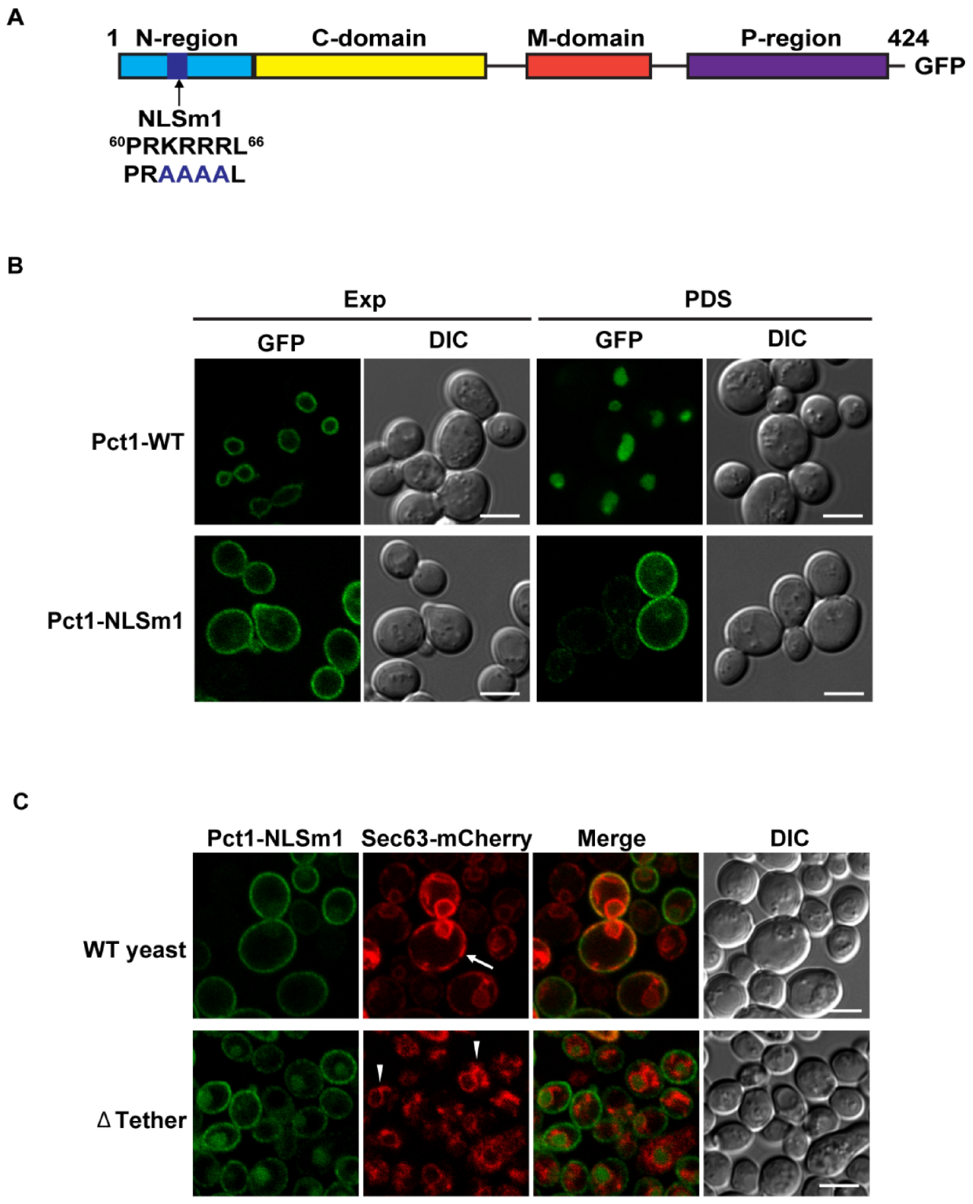
A
1 N-region
C-domain
M-domain
P-region
424
GFP
NLSm1
60PRKRRRL66
PRAAAAL
B
Exp
PDS
GFP
DIC
GFP
DIC
Pct1-WT
Pct1-NLSm1
C
Pct1-NLSm1
Sec63-mCherry
Merge
DIC
WT yeast
Δ Tether

Figure S4. Related to figure 3. Abrogation of the Pct1 nuclear localisation signal (NLS1) sequence inhibits Pct1 nuclear translocation.

(A) Diagram showing the putative nuclear localization sequence (NLS) of Pct1. Basic residues of the NLS motif were mutated to alanine (blue) in NLSm1-Pct1-GFP. C-domain, catalytic domain; M-domain, membrane binding domain; P-region, phosphorylated region.

(B) *pct1*Δ cells expressing the GFP-tagged wild-type Pct1 (Pct1-WT) or NLSm1 mutant (Pct1-NLSm1) were grown to the indicated growth phase and imaged as described in Methods. Scale bars, 5 µm.

(C) GFP-tagged Pct1-NLSm1 and Sec63-mCherry (ER marker) were expressed in the Δtether strain and the corresponding wild type (WT) yeast strain, grown to exponential phase and imaged as described in Methods. White arrows mark the normal cortical ER in WT strain and arrowheads indicate the collapsed cortical ER in the Δtether cells. Scale bars, 5 µm.

Fig. S5

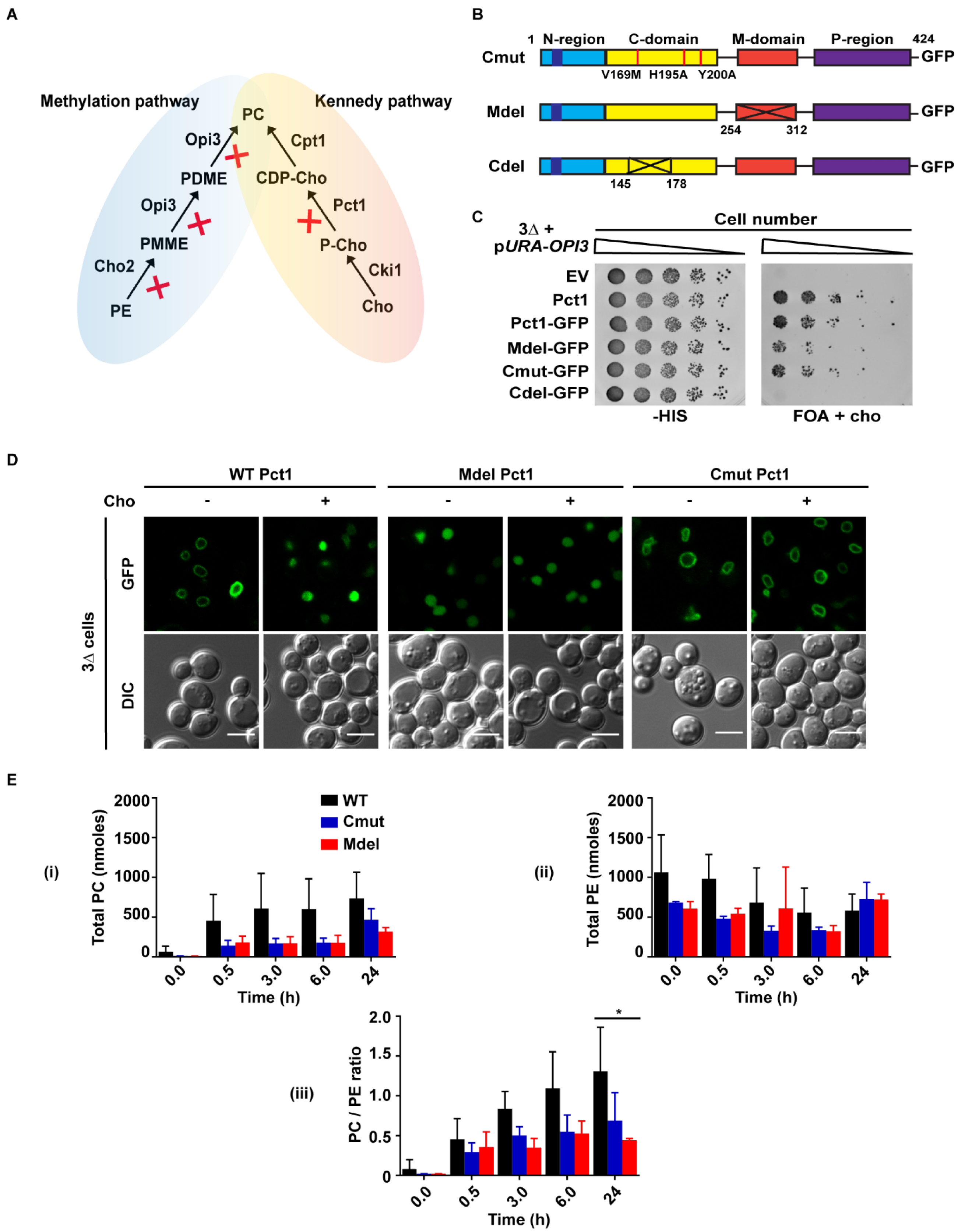
A
Methylation pathway
Kennedy pathway
PC
Opi3
Cpt1
PDME
CDP-Cho
Opi3
Pct1
PMME
P-Cho
Cho2
Cki1
PE
Cho
B
1 N-region C-domain M-domain P-region 424
Cmut
GFP
V169M H195A Y200A
Mdel
GFP
254 312
Cdel
GFP
145 178
C
3Δ + pURA-OPI3
Cell number
EV
Pct1
Pct1-GFP
Mdel-GFP
Cmut-GFP
Cdel-GFP
-HIS
FOA + cho
D
WT Pct1
Mdel Pct1
Cmut Pct1
Cho
3Δ cells
GFP
DIC
E
(i)
Total PC (nmoles)
WT
Cmut
Mdel
Time (h)
(ii)
Total PE (nmoles)
Time (h)
(iii)
PC / PE ratio
Time (h)

Figure S5. Related to figure 5. Validation of the 3Δ (*cho2Δ opi3Δ pct1Δ*) *Saccharomyces cerevisiae* model.

(A) Schematic of the enzymes involved in PC biosynthesis. Deletion of *CHO2* and *OPI3* blocks the methylation pathway while deletion of Pct1 blocks the CDP-choline branch of the Kennedy pathway. PC, phosphatidylcholine; PE, phosphatidylethanolamine; P-cho, Phosphocholine; CDP-Cho, CDP-choline, PMME, phosphatidyl-N-mono-methylethanolamine; PDME, phosphatidyl-N, N dimethylethanolamine.

(B) Schematic illustration of the Pct1 domain organisation indicating the mutations in the Cmut, Mdel and Cdel Pct1 mutants. C-domain, Catalytic domain; M-domain, Membrane binding domain. Substitution mutations are indicated with red bars and targeted deletions are indicated with crossed boxes.

(C) 3Δ cells carrying Ycplac33-*URA3-OPI3* were transformed with a *CEN/HIS3* plasmid expressing either wild-type Pct1, Pct1-GFP or mutant- Mdel-GFP, Cmut-GFP and Cdel-GFP. Empty *CEN/HIS3* plasmid (EV) was used as a negative control. Cells were grown to exponential phase and serial dilutions of liquid cultures were spotted onto SC plates lacking histidine (-HIS) or supplemented with FOA.

(D) 3Δ cells expressing GFP-tagged WT, Mdel or Cmut Pct1 were grown to exponential growth phase with (+) or without (-) choline and imaged as described in Methods. DIC images are shown for context. Scale bars, 5 µm.

(E) 3Δ cells expressing GFP-tagged WT, Mdel or Cmut Pct1 were grown for 24 hours (h) followed by addition of 1 mM choline. Cells were collected before (0.0 h) and after choline supplementation at the indicated time points for lipidomic analysis. (i) PC and (ii) PE levels at the indicated time points are shown in nmoles (per 50 mg yeast) relative to internal standards. (iii) PC/PE ratio at the indicated time points. Data are shown as mean ± SD from independent repeats (n=8 for WT; n=3 each for Cmut and Mdel Pct1). *P <0.05 at 24 h using one-way ANOVA.

Fig. S6

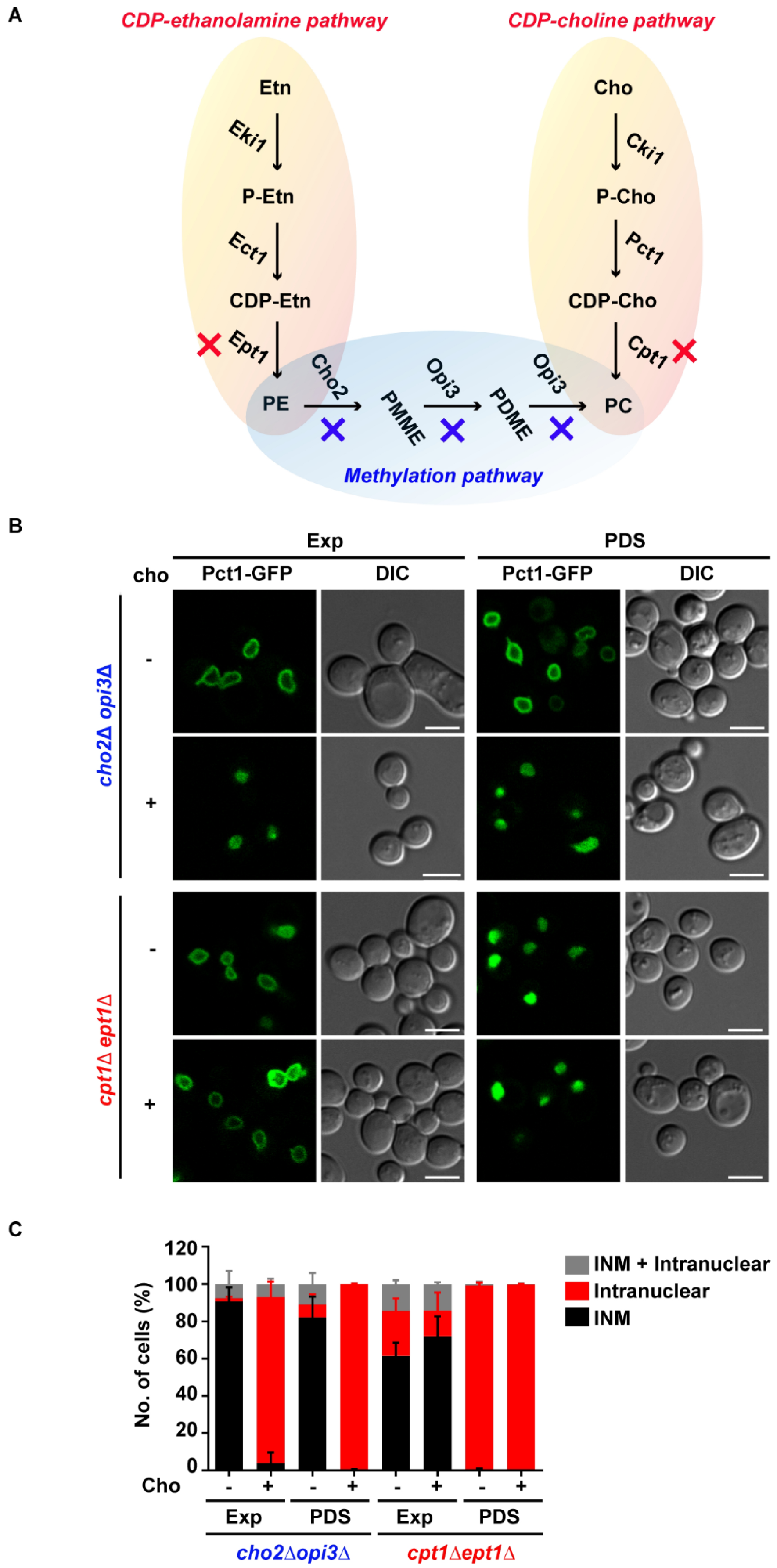

A
CDP-ethanolamine pathway
CDP-choline pathway
Etn
Eki1
P-Etn
Ect1
CDP-Etn
Ept1
PE
Cho2
PMME
Opi3
PDME
Opi3
PC
Cho
Cki1
P-Cho
Pct1
CDP-Cho
Cpt1
Methylation pathway
B
Exp
PDS
cho
Pct1-GFP
DIC
Pct1-GFP
DIC
cho2Δ opi3Δ
cpt1Δ ept1Δ
-
+
-
+
C
120
100
80
60
40
20
0
No. of cells (%)
INM + Intranuclear
Intranuclear
INM
Cho
- + - + - + - +
Exp
PDS
Exp
PDS
cho2Δopi3Δ
cpt1Δept1Δ

Figure S6. Related to figure 5. Pct1 localization in response to PC levels is independent of the source of PC.

(A) Schematic illustration of PC synthesis through the methylation and Kennedy pathway. The enzymes deleted in the *cho2*Δ*opi3*Δ mutant (blue cross) and in the *cpt1*Δ*ept1*Δ mutant (red cross) are indicated. Cho, choline; PC, phosphatidylcholine; PE, phosphatidylethanolamine; P-cho, Phosphocholine; CDP-Cho, CDP-choline; Etn, ethanolamine; P-Etn, Phosphoethanolamine; CDP-Etn, CDP-ethanolamine; PMME, phosphatidyl-N-mono- methylethanolamine; PDME, phosphatidyl-N,N dimethyl ethanolamine.

(B) Pct1-GFP was expressed in a *cho2*Δ*opi3*Δ mutant (defective in the methylation pathway) or a *cpt1*Δ*ept1*Δ mutant (defective in the CDP-choline pathway). Cells were grown to the indicated growth phase with (+) or without (-) excess choline and imaged as described in Methods. DIC images are shown for context. Scale bars, 5 µm.

(C) Quantification of the Pct1-GFP localization shown in (B). Data are mean ± SD based on random fields from three independent experiments. ~100 cells were counted at each time point in each independent experiment.

Fig. S7

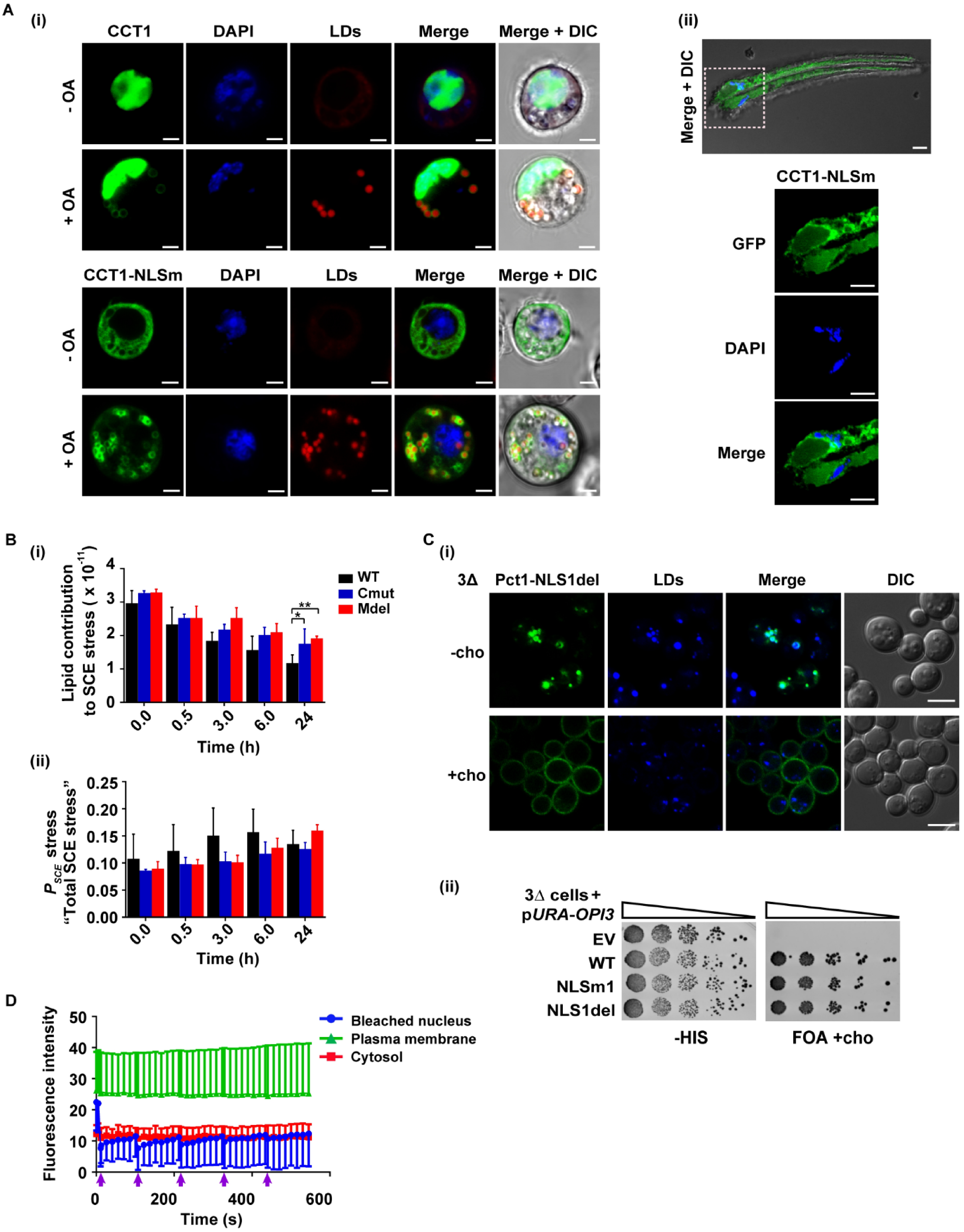
A
(i)
CCT1
DAPI
LDs
Merge
Merge + DIC
- OA
+ OA
CCT1-NLSm
DAPI
LDs
Merge
Merge + DIC
- OA
+ OA
(ii)
Merge + DIC
CCT1-NLSm
GFP
DAPI
Merge
B
(i)
Lipid contribution to SCE stress (x 10^-11)
WT
Cmut
Mdel
Time (h)
(ii)
P_SCE stress "Total SCE stress"
Time (h)
C
(i)
3Δ
Pct1-NLS1del
LDs
Merge
DIC
-cho
+cho
(ii)
3Δ cells + pURA-OPI3
EV
WT
NLSm1
NLS1del
-HIS
FOA +cho
D
Fluorescence intensity
Bleached nucleus
Plasma membrane
Cytosol
Time (s)

Figure S7. Related to figure 6 and 7. Abrogation of the predicted NLS of CCT1 and Pct1 prevents nuclear localization.

(A) The nuclear targeting signal in CCT1 was mutated to alanine residues (^{34}RKR^{35}→AAA) in the CCT1-NLSm mutant. (i) *Drosophila* S2 cells expressing C-terminal GFP-tagged wild type CCT1 (CCT1) or CCT1-NLS mutant (CCT1-NLSm) were grown with (+OA) or without (-OA) oleic acid for 24 hours. Live cell imaging in cells stained for lipid droplet (LDs) and nuclear (DAPI) visualization was performed as described in Methods. The merged fluorescent channels are shown with DIC (Merge + DIC) for context. Scale bars, 2.5 µm. (ii) GFP-tagged CCT1-NLSm was transgenically expressed in *D. melanogaster* eyes under the *Rhodopsin* (*Rh1*) promoter. Individual ommatidia were isolated and imaged as described in methods. All panels are zoomed in images from the boxed region in the 'merge + DIC' panel. Scale bars, 10 µm.

(B) 3Δ yeast cells expressing either WT Pct1-GFP or mutant Cmut- and Mdel-GFP were grown for 24 h followed by addition of 1 mM choline. Cells were collected before (0.0 h) and after choline supplementation at the indicated time points for lipidomics analyses and the (i) lipid-driven and (ii) total SCE stress in the cells was calculated as described in Methods. These estimates correspond to lipidomics data depicted in fig. S5E. Data are shown as mean ± SD from independent repeats (n=8 for WT; n=3 each for Cmut and Mdel Pct1). *P <0.05, **P <0.01 at 24 h using one-way ANOVA with Bonferroni multiple comparisons.

(C) The NLS1 region (amino acids 60-66) of Pct1 was deleted in the Pct1-NLS1del-GFP mutant and expressed in 3Δ cells. (i) Cells were grown with (+) or without (-) choline for 24 hours and stained to visualize lipid droplets (LDs), and imaged as described in Methods. DIC images are shown for context. Scale bar, 5 µm. (ii) The cells were grown to exponential phase and serial dilutions of liquid cultures were spotted onto plates lacking histidine (-HIS) or supplemented with FOA and choline (FOA + cho).

(D) *pct1*Δ cells expressing Pct1-NLSm1-GFP were repeatedly photobleached in the nucleus and there was no loss of GFP fluorescence at the plasma membrane, indicating little or no trafficking of Pct1-

NLSm1-GFP from the PM to the nucleus. Data are mean ± SD from three independent experiments (5-8 cells each). The GFP fluorescence intensities reported here were corrected for loss of fluorescence during imaging as described in Methods. Arrows on X-axis indicate time-points of each bleaching event.
