# Supplementary material for: PCYT1A Regulates Phosphatidylcholine Homeostasis from the Inner Nuclear Membrane in Response to Membrane Stored Curvature Elastic Stress
Source: Dev Cell. 2018 May 21;45(4):481–495.e8. doi: 10.1016/j.devcel.2018.04.012 (PMC5971203; doi:10.1016/j.devcel.2018.04.012)
Supplement: Document S2. Article plus Supplemental Information [file mmc2.pdf]

Article

Developmental Cell

PCYT1A Regulates Phosphatidylcholine Homeostasis from the Inner Nuclear Membrane in Response to Membrane Stored Curvature Elastic Stress

Graphical Abstract

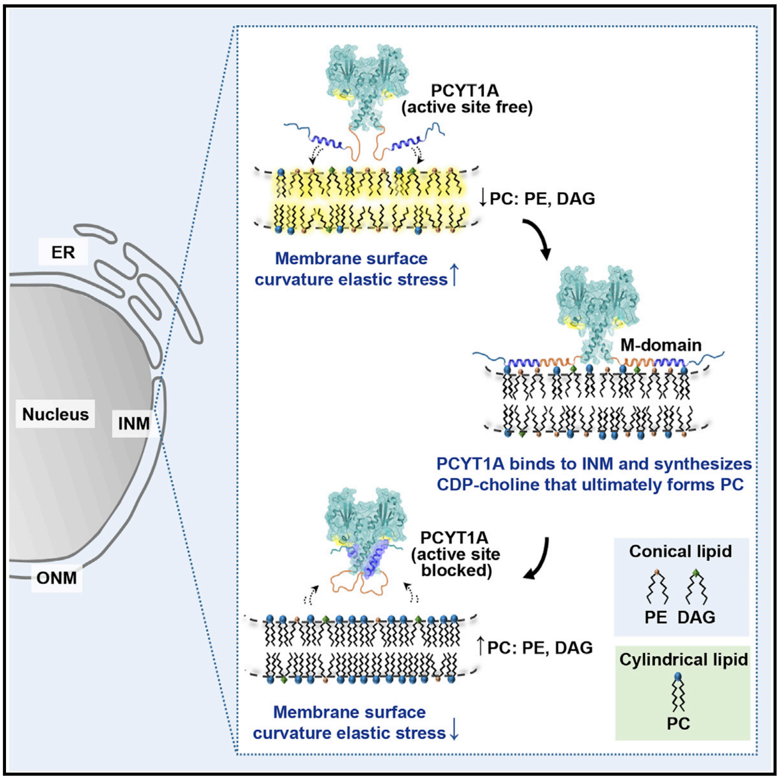

Highlights

- PCYT1A catalyzes the rate-limiting reaction in phosphatidylcholine (PC) synthesis
- PC deficiency increases membrane lipid stored curvature elastic (SCE) stress
- PCYT1A uses a nascent amphipathic helix to sense SCE in the nuclear membrane
- PCYT1A mutants lacking a nuclear localization signal rescue PCYT1A-deficient cells

Authors

Afreen Haider, Yu-Chen Wei, Koini Lim, ..., Roger C. Hardie, Symeon Siniossoglou, David B. Savage

Correspondence

ss560@cam.ac.uk (S.S.), dbs23@medschl.cam.ac.uk (D.B.S.)

In Brief

Phosphatidylcholine (PC) is the major constituent of cellular membranes. Haider et al. show that PCYT1A, the rate-limiting enzyme of PC synthesis, translocates onto the inner nuclear membrane in response to increased PC need in yeast, fly, and mammalian cells. This PCYT1A re-localization is governed by membrane stored curvature elastic stress.

Haider et al., 2018, Developmental Cell *45*, 481–495
May 21, 2018 © 2018 The Authors. Published by Elsevier Inc.
https://doi.org/10.1016/j.devcel.2018.04.012

CellPress

Developmental Cell
Article

CellPress

PCYT1A Regulates Phosphatidylcholine Homeostasis from the Inner Nuclear Membrane in Response to Membrane Stored Curvature Elastic Stress

Afreen Haider,[1,8] Yu-Chen Wei,[1,8] Koini Lim,[1,8] Antonio D. Barbosa,[2,8] Che-Hsiung Liu,[3] Ursula Weber,[4] Marek Mlodzik,[4] Kadri Oras,[5] Simon Collier,[5] M. Mahmood Hussain,[6] Liang Dong,[1] Satish Patel,[1] Anna Alvarez-Guaita,[1] Vladimir Saudek,[1] Benjamin J. Jenkins,[1] Albert Koulman,[1] Marcus K. Dymond,[7] Roger C. Hardie,[3] Symeon Siniossoglou,[2,9,*] and David B. Savage[1,9,10,*]

[1]Metabolic Research Laboratories, Wellcome Trust-Medical Research Council Institute of Metabolic Science, University of Cambridge, Cambridge CB2 0QQ, UK
[2]Cambridge Institute for Medical Research, University of Cambridge, Cambridge CB2 0XY, UK
[3]Department of Physiology, Development and Neuroscience, University of Cambridge, Cambridge CB2 3EG, UK
[4]Department of Cell, Developmental and Regenerative Biology, Icahn School of Medicine at Mount Sinai, New York City, NY 10029, USA
[5]Department of Genetics, University of Cambridge, Cambridge CB2 3EH, UK
[6]Departments of Cell Biology and Pediatrics, State University of New York Downstate Medical Center, Brooklyn, NY 11203, USA
[7]Division of Chemistry, School of Pharmacy and Biomolecular Sciences, University of Brighton, Brighton BN2 4GJ, UK
[8]These authors contributed equally
[9]Senior author
[10]Lead Contact
*Correspondence: ss560@cam.ac.uk (S.S.), dbs23@medschl.cam.ac.uk (D.B.S.)
https://doi.org/10.1016/j.devcel.2018.04.012

SUMMARY

Cell and organelle membranes consist of a complex mixture of phospholipids (PLs) that determine their size, shape, and function. Phosphatidylcholine (PC) is the most abundant phospholipid in eukaryotic membranes, yet how cells sense and regulate its levels *in vivo* remains unclear. Here we show that PCYT1A, the rate-limiting enzyme of PC synthesis, is intranuclear and re-locates to the nuclear membrane in response to the need for membrane PL synthesis in yeast, fly, and mammalian cells. By aligning imaging with lipidomic analysis and data-driven modeling, we demonstrate that yeast PCYT1A membrane association correlates with membrane stored curvature elastic stress estimates. Furthermore, this process occurs inside the nucleus, although nuclear localization signal mutants can compensate for the loss of endogenous PCYT1A in yeast and in fly photoreceptors. These data suggest an ancient mechanism by which nucleoplasmic PCYT1A senses surface PL packing defects on the inner nuclear membrane to control PC homeostasis.

INTRODUCTION

Eukaryotic cells maintain their membrane lipid composition within narrow limits. In poikilothermic organisms, changes in membrane composition are essential to preserve membrane fluidity in the cold, whereas in homoeothermic organisms membrane properties are critical for the maintenance of organelle identity and function (Ernst et al., 2016). This is particularly important for protein recognition of the specific exofacial membrane features of intracellular organelles. Recent progress in understanding how cells sense and regulate membrane composition includes characterization of the role of SREBP-2 in regulating endoplasmic reticulum (ER) cholesterol levels (Brown et al., 2017; Eberle et al., 2004) and of the role of Mga2 in sensing ER membrane acyl chain saturation (Covino et al., 2016; Ernst et al., 2016). In each of these cases, the "sensors" are transmembrane proteins suggested to be detecting primarily the hydrophobic interior milieu of phospholipid (PL) bilayer membranes. While the fatty acyl side chains of PLs contribute to the hydrophobic interior of bilayer membranes, PLs also have distinctive head groups which influence both membrane PL packing and the membrane surface topology. *In vitro* studies have previously suggested that peripheral proteins involved in PL metabolism may directly sense membrane properties in order to maintain membrane homeostasis, but exactly how this occurs *in vivo* remains uncertain (Cornell, 2016; Cornell and Ridgway, 2015).

PC is the most abundant PL of eukaryotic cell membranes comprising 30%–60% of total PL mass. Because PLs are the building blocks of membranes, bulk PC production must be tightly coordinated with cellular growth status: rapidly proliferating cells have a high demand for PC synthesis to support biomass production. PC synthesis is also required at key developmental stages in specialized cell types, such as cells that undergo extensive membrane proliferation as in photoreceptors (PRs) (Young, 1967) or extensive ER membrane remodeling and expansion for immunoglobulin or hormone secretion (Fagone et al., 2007). PC is also secreted in lipoproteins, bile and lung surfactant, as well as being a source of lipid second messengers such as diacylglycerol (DAG) (van der Veen et al., 2017; Cornell and Ridgway, 2015; Cole et al., 2012).

This is an open access article under the CC BY license (http://creativecommons.org/licenses/by/4.0/).

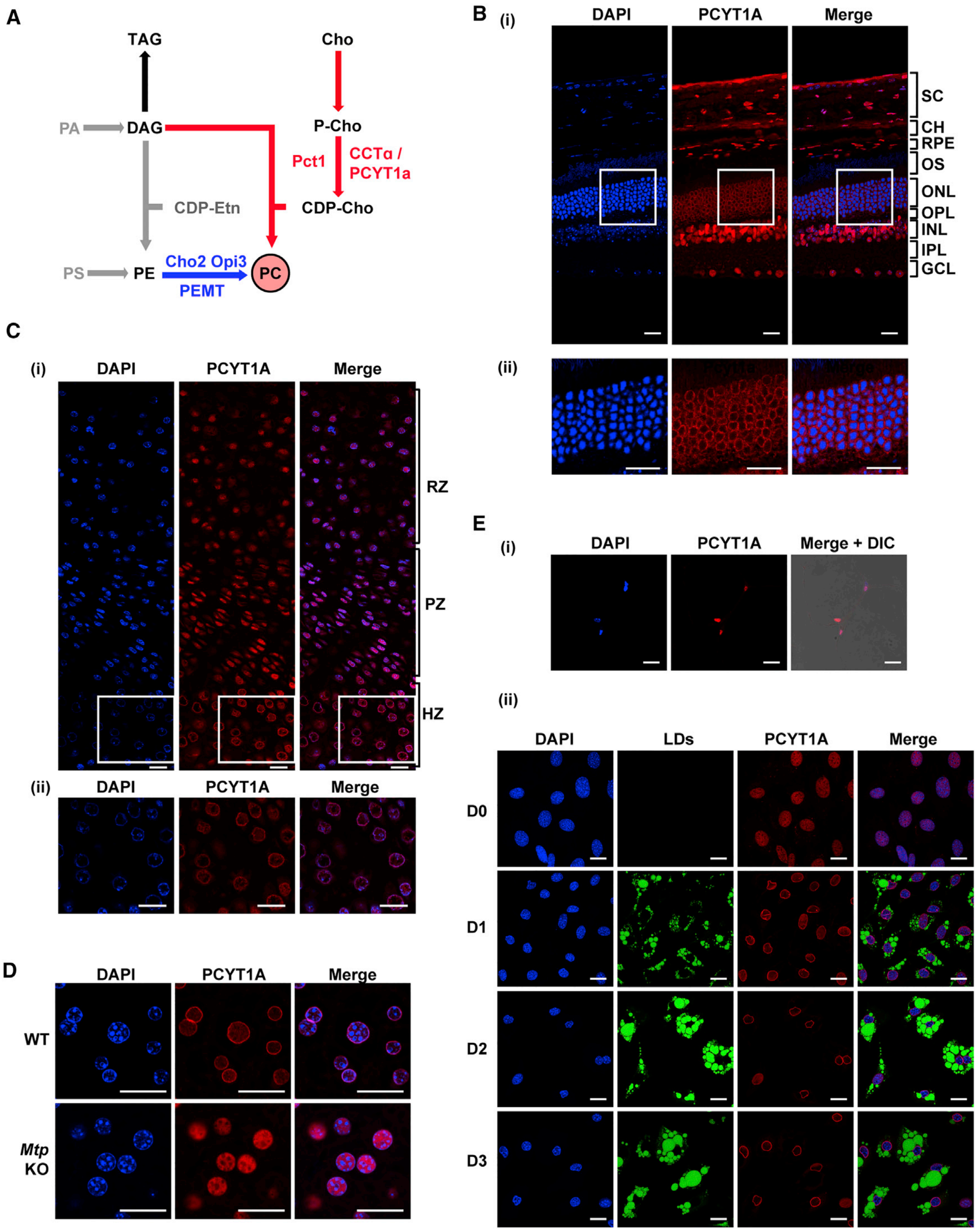

Figure 1. Immunostaining of Endogenous PCYT1A in Selected Mouse Tissues

(A) Simplified schematic of the two major PC biosynthetic pathways in eukaryotes. The CDP-choline (Kennedy) pathway is shown in red and the methylation pathway in blue. Key enzymes discussed in the text are shown in upper case (mammals) or lower case (yeast). Pct1/CCTα/PCYT1A, choline-phosphate cytidylyltransferase A; P-Cho, phosphocholine; CDP-Cho, cytidine diphosphate-choline; CDP-Etn, cytidine diphosphate-ethanolamine; PA, phosphatidic acid; DAG, diacylglycerol; TAG, triacylglycerol; PC, phosphatidylcholine; PE, phosphatidylethanolamine; PS, phosphatidylserine; Cho, choline.

(B) (i) Immunostaining of adult mouse retina indicates that PCYT1A localizes to the nuclear membrane in the outer nuclear layer. SC, sclera; CH, choroid; RPE, retinal pigmented epithelium; OS, outer segments of rods and cones; ONL, outer nuclear layer; OPL, outer plexiform layer; INL, inner nuclear layer; IPL, inner plexiform layer; GCL, ganglion cell layer. (ii) Zoomed-in images of the indicated field in (i).

(C) (i) In the femoral growth plate of 15-day-old mice, PCYT1A localizes to the nuclear membrane of chondrocytes in the hypertrophic (HZ) but not in the resting (RZ) or proliferative (PZ) zone of growth plates. (ii) Zoomed in images of the indicated field in (i).

(legend continued on next page)

Two pathways are responsible for the *de novo* synthesis of PC, namely the phosphatidylethanolamine (PE) methyltransferase and the Kennedy pathways. The latter constitutes the major route for PC synthesis in most eukaryotes and involves three sequential enzymatic reactions leading to condensation of choline and DAG into PC (Figure 1A). It is widely accepted that the rate-limiting step of the Kennedy pathway is the formation of CDP-choline, catalyzed by the choline phosphate cytidylyltransferase (CCT) (Figure 1A) (Sundler et al., 1972). CCT is highly conserved in eukaryotes (Cornell and Ridgway, 2015); budding yeast express one CCT enzyme, Pct1, while higher eukaryotes express two: PCYT1A (also known as CCTα in mammals; CCT1 in *Drosophila*), which is ubiquitously expressed, and PCYT1B (CCTβ in mammals; CCT2 in *Drosophila*), which is less widely expressed and lacks the nuclear localization signal (NLS) present in PCYT1A. The split into two paralogs must have occurred very early in evolution as even the most primitive metazoan *Trichoplax adherens* contains two CCT genes. However, a phylogenetic tree indicates that the two paralogs evolved together and remain closer to each other rather than to their orthologs (Figure S1A). The Pfam database (http://pfam.xfam.org/family/PF01467) lists many homologous proteins from *Bacteria* and *Archaea*, but none of them has been annotated as CCT thus far. A detailed biochemical and structural study (Kwak et al., 2002) identified an enzyme with CCT activity in *Streptococcus pneumoniae* that is evolutionarily unrelated to the eukaryotic ones and has close homologs in many *Bacteria* and *Archaea*.

Surprisingly, while both its substrate and product are water-soluble, PCYT1A partitions between soluble and membrane-associated forms. Structural studies suggested a model whereby membrane association rapidly facilitates PCYT1A catalytic activity by promoting an unstructured loop to fold into a helix causing removal of an adjoining helix, which otherwise prevents substrate access to the catalytic pocket of the dimeric enzyme (Lee et al., 2009). Several similarly unstructured motifs that fold into amphipathic helices upon encountering membranes with specific features have been reported in proteins with a range of functions (Cornell, 2016; Magdeleine et al., 2016; Antonny, 2011; Karanasios et al., 2010; Drin et al., 2007; Bigay et al., 2005). *In vitro* studies have shown that membrane association and catalytic activation of purified PCYT1A/B is induced by conically shaped lipids such as DAG or PE, or by negatively charged PLs such as phosphatidic acid, or phosphatidylserine (PS) (Taneva et al., 2005; Davies et al., 2001; Attard et al., 2000; Arnold and Cornell, 1996). This suggests a model in which PCYT1A/B would sense a relative paucity of PC relative to other lipids, such as PE or DAG, resulting in its membrane association, activation, and alleviation of the membrane stress evoked by conically shaped lipids.

Although the enzymology of PCYT1A/B and the biochemical pathways that generate PC have been well described, exactly how cells detect the levels of PC within their membranes to maintain homeostasis *in vivo* remains unclear (Cornell, 2016). Our interest in this question was stimulated by recent reports linking biallelic loss-of-function mutations in human *PCYT1A* to an intriguing spectrum of specific phenotypes including retinal dystrophy, spondylometaphyseal (growth plate) dysplasia, lipodystrophy, and non-alcoholic fatty liver disease (Testa et al., 2017; Hoover-Fong et al., 2014; Payne et al., 2014; Wong, 2014; Yamamoto et al., 2014). At least some of these mutations result in almost complete loss of PCYT1A expression, and significantly impair PC synthesis in primary skin fibroblasts (Payne et al., 2014). These data suggest that PCYT1A is particularly important in the affected cell types, so we began by determining the site of PCYT1A activation in these cells/tissues. We then established a genetically tractable system to monitor Pct1 in yeast and combined it with studies in fly models. We find that Pct1/CCT1/PCYT1A activity takes place predominantly (and perhaps exclusively) at the inner nuclear membrane and, in all cell types tested, is promoted by a cellular requirement for membrane biogenesis or remodeling. Our data suggest that *in vivo* Pct1 primarily senses hydrophobic cavities or "packing defects" within the membrane rather than PC itself. Membrane packing defects typically occur when conically shaped PLs, such as PE or DAG, accumulate in the membrane (McDonald et al., 2015; Vanni et al., 2014; Vamparys et al., 2013). Their presence in one of the constituent PL monolayers of a bilayered membrane induces a physical torque (or bending) stress known as stored curvature elastic (SCE) stress, which would ultimately result in phase transition and pore formation if left unchecked. By binding to the membrane, the amphipathic helical domain of PCYT1A temporarily alleviates this stress itself until sufficient PC is generated to restore membrane stability; thus, constituting an ancient biophysical mechanism to regulate membrane PL homeostasis.

RESULTS

PCYT1A Localizes to the Nuclear Envelope in Cells Adversely Affected by Biallelic PCYT1A Mutations

Given the compelling structural and *in vitro* evidence that PCYT1A is activated by membrane association, we hypothesized that PCYT1A was likely to be membrane-bound at key developmental stages that require organelle membrane biogenesis. To test this, we initially focused on tissues affected in *PCYT1A*-linked human diseases, namely retina, bone growth plates, liver, and adipose tissue (Testa et al., 2017; Hoover-Fong et al., 2014; Payne et al., 2014; Wong, 2014; Yamamoto et al., 2014), where, accordingly, PCYT1A activity is likely to be physiologically important. We validated an antibody that reliably detects endogenous PCYT1A (Figure S1B) and immunostained cells from each of the affected tissues in mice (Figures 1B–1E). In all cases, PCYT1A immunostaining was confined to the nucleus. In the eye, PCYT1A was intranuclear in all layers, but strikingly restricted to the nuclear rim in the outer nuclear layer where the nuclei of PRs are located (Figure 1B). PRs have a very distinctive morphology characterized by an extensive outer segment

(D) In *ad-libitum* chow-fed adult mice, PCYT1A localizes to the nuclear membrane in wild-type (WT) but not in *Mtp* knockout hepatocytes, which have impaired lipoprotein synthesis.
(E) (i) PCYT1A localizes to the intranuclear region of adult mouse inguinal white adipocytes but translocates to the nuclear membrane upon adipogenic induction in OP9 cells (ii). Lipid droplets (LDs) were stained with BODIPY (green) as described in the STAR Methods. D0–D3 indicate day after onset of differentiation. Scale bars, 20 μm. See Figure S1.

composed of multiple membrane layers housing the light-sensitive rhodopsin pigment. These membranes are very dynamic, as light-induced isomerization of rhodopsin ultimately leads to protein and membrane turnover (Ding et al., 2015; Molday and Moritz, 2015). Notably, we did not see any evidence of cell membrane- or ER-specific PCYT1A staining. In sections of femoral growth plates, nuclear rim staining was apparent in the hypertrophic zone but not in the resting or proliferative zones (Figure 1C). The abrupt morphological change from flattened chondrocytes in the proliferative zone to spherical chondrocytes in the hypertrophic zone is associated with a 5-fold enlargement in cell volume, which may account for PCYT1A activation (Brighton et al., 1973). In the liver, PCYT1A was localized to the nuclear envelope in both fed and fasting mice (Figures 1D and S1C), which may reflect the need for PC synthesis to sustain lipoprotein production. In keeping with this hypothesis, PCYT1A staining was largely intranuclear in liver sections from liver-specific *Mtp* (microsomal triglyceride transfer protein) knockout mice (Figure 1D), which do not secrete lipoproteins (Khatun et al., 2012). In murine adipose tissue we only detected intranuclear PCYT1A immunostaining (Figure 1Ei), but in cultured OP9 adipocytes nuclear envelope staining was observed during the course of differentiation in the presence (Figure 1Eii) or absence (Figure S1D) of oleic acid, in keeping with a report from Aitchison et al. (2015). PCYT1A staining was not detected on lipid droplets (LDs) in cultured or ''*in vivo*'' adipocytes. Taken together these data indicate that PCYT1A is associated with the nuclear envelope in tissues associated with *PCYT1A*-linked disease phenotypes, possibly reflecting their pronounced PC demands.

Drosophila CCT1 Associates with the Nuclear Envelope and Is Required for PR Development and Function

To date, the human phenotype most frequently associated with biallelic *PCYT1A* mutations is cone-rod retinal dystrophy (Testa et al., 2017; Hoover-Fong et al., 2014; Wong, 2014; Yamamoto et al., 2014). *Drosophila* PRs provide a tractable system in which to explore this phenotype in detail as, despite significant morphological and biochemical differences with mammalian PRs (e.g., microvillar as opposed to ciliary membrane expansion), they were used to first identify the mechanisms responsible for mammalian retinal diseases (Colley et al., 1995; Kurada and O'Tousa, 1995) and also undergo extensive membrane remodeling during development. We began by documenting the localization of transgenically expressed GFP-tagged fly CCT (CCT1 and CCT2) and human CCT (hPCYT1A) homologs in live isolated ommatidia, which constitute the basic units of the compound eye of *Drosophila* and consist of eight PRs surrounded by supporting cells. While CCT1-GFP and hPCYT1A-GFP were intranuclear, CCT2-GFP was cytoplasmic (Figure 2A). Furthermore, whereas CCT1-GFP appeared to localize primarily within the nucleoplasm at stage P11 of pupal development, it was more peripherally distributed from P12 onward (Figure S2A). The latter stage corresponds to the time when ER expansion peaks during formation of the microvilli, which collectively constitute the light-sensing apical membrane of each PR, the rhabdomere, which houses rhodopsin (Hardie and Juusola, 2015). In contrast, CCT2-GFP remained cytoplasmic throughout pupal development (Figure S2A).

To assess the requirement for CCT1 in *Drosophila* PRs we selectively reduced CCT1 expression in fly eyes using two independent strategies. Firstly, siRNA-mediated knockdown using two independent RNAi stocks modestly reduced light responses as assessed by electroretinograms (ERGs), whereas combined use of both resulted in near blindness (Figures 2B and 2C). These findings were corroborated by corresponding changes in rhodopsin expression (Figure 2D), and correlate with the failure of rhabdomere formation, which was documented using transmission electron microscopy (TEM) (Figure 2E). In contrast, eye-specific knockdown of CCT2 had little effect on light response, rhodopsin levels, or rhabdomere formation (Figures 2B–2E). Secondly, we employed a genetic *FLP-FRT*/GMR-*hid* method (Stowers and Schwarz, 1999) to generate mosaic flies that have eye tissue specifically homozygous for CCT-null mutant alleles $CCT1^{179}$ (*CCT1* deleted) or CCT^{299} (both *CCT1* and *CCT2* deleted) (Gupta and Schupbach, 2003). Complete loss of CCT1 or both CCT1 and 2 in the eye essentially eliminated all response to light in the ERG and drastically reduced rhodopsin expression, while TEM confirmed the dramatic impact of these deletions on rhabdomere development (Figure S2B). It is worth noting that this perturbation does not appear to affect PR survival, but it does highlight the evolutionarily conserved importance of intranuclear CCT1 in the development of this particularly membrane-rich cell type.

Yeast Pct1-GFP Associates with the Inner Nuclear Membrane

To understand how PCYT1A membrane localization and activation is triggered at the molecular level *in vivo*, we took advantage of the genetic plasticity of the yeast *Saccharomyces cerevisiae*. Similar to higher eukaryotes, GFP-tagged Pct1, the yeast ortholog of PCYT1A, resides on the nuclear membrane in rapidly proliferating cells but ''falls off'' during post-diauxic shift (PDS) phase, when glucose is exhausted and lipid metabolism is rewired toward storage (Figure 3A, Barbosa et al., 2015). In yeast, both the methylation and Kennedy pathways contribute to PC synthesis, but the contribution of the latter increases with the supplementation of choline in the medium (McMaster and Bell, 1994). Interestingly, Pct1-GFP is released from the nuclear membrane into the nucleoplasm on choline supplementation (Figure 3A), which increases PC levels and the PC/PE ratio (Figures 3B and 3C).

The specific perinuclear Pct1-GFP signal resembles the localization of yeast inner nuclear membrane proteins, which are excluded from the peripheral ER network (Laba et al., 2014). Using the anchor-away method to sequester a key importin, Kap60, which is known to facilitate Pct1 nuclear import (MacKinnon et al., 2009; Haruki et al., 2008), we showed that Pct1-GFP is released from the nuclear membrane to the nucleoplasm upon glucose starvation (Figure S3), confirming Pct1-GFP inner nuclear membrane localization. Also, we found that mutating a stretch of four basic residues (^{60}PR$\underline{\text{KRRR}}$L^{66}; henceforth called NLS mutant 1 [NLSm1]) to alanines leads to its re-localization to the cell periphery (Figures S4A and S4B). As Pct1-NLSm1 remains in the cell periphery in the Δtether strain, in which six proteins that tether the cortical ER (cER) to the plasma membrane (PM) are deleted causing the collapse of the cER and its accumulation in the cytoplasm (Manford et al., 2012), it is likely that Pct1-NLSm1 associates with the PM, and not the cER (Figure S4C).

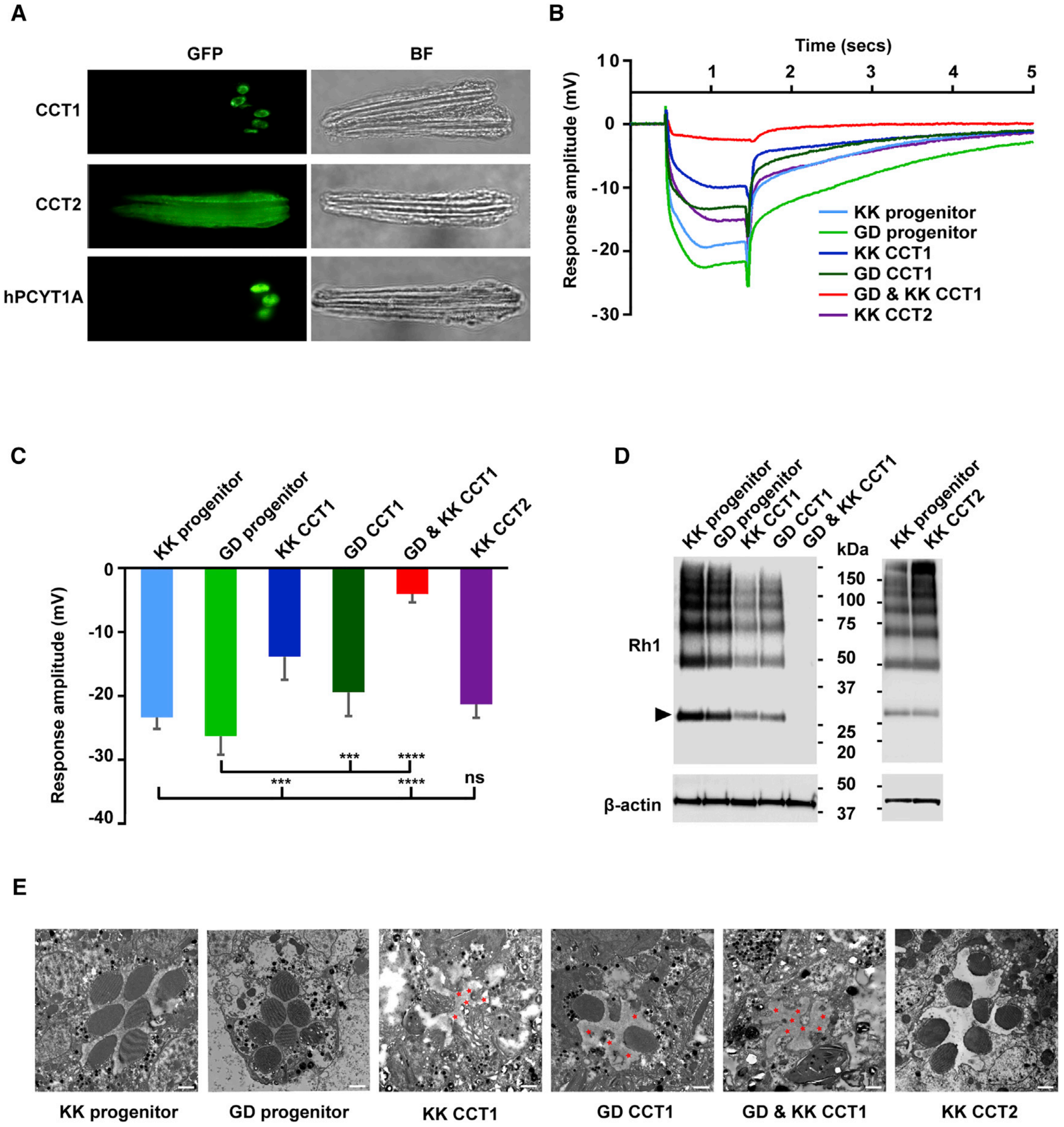

Figure 2. The Fly CCTα Homolog, CCT1, Is Localized within the Nucleus of PRs, and Eye-Specific Knockdown of CCT1 Inhibits Light Response in Flies

(A) Individual ommatidia from transgenic flies selectively expressing GFP-tagged fly CCT1, CCT2, or the human CCTα (hPCYT1A) homologs in the retina, under a *Rhodopsin* (*Rh1*) promoter, were imaged as described in the STAR Methods. BF, bright-field images. (B–E) Flies with eye-specific single (GD CCT1 or KK CCT1) and double knockdown of CCT1 (GD and KK CCT1) or knockdown of CCT2 (KK CCT2). Flies with no siRNA genes served as negative control (KK/GD progenitor).

(B) Response to light activation was assessed by ERG (electroretinogram) scans at increasing intensities of light. Representative traces obtained from a submaximal 1-s flash from a single fly of each genotype are shown.

(C) Response amplitude generated in ERG recordings at maximal intensity of light was severely reduced in eyes with CCT1 double knockdown. Data are means ± SD from four to eight flies of each genotype. One-way ANOVA with Bonferroni multiple comparison, ***$p < 0.001$; ****$p < 0.0001$.

(D) Immunoblotting of lysates from 50 heads of each genotype using rhodopsin antibody; β-actin was used as a loading control. The level of rhodopsin (arrowhead marks mature form) was negligible in flies with CCT1 double knockdown (GD and KK CCT1). Blot shown is representative from three independent batches.

(E) Rhabdomere ultrastructure was analyzed by transmission electron microscopy. Cross-sections show normal rhabdomere stacks in the control and CCT2 knockdown flies, while the rhabdomere stacks were loosely stacked or completely absent (red stars) in the CCT1 knockdown flies. Scale bars, 500 nm.

See Figure S2.

Pct1-GFP Is Confined to the Nucleus

We next addressed the dynamics of the nuclear membrane association of Pct1. Given that (1) mammalian cells express a cytoplasmic isoform, PCYT1B, (2) Pct1-NLSm1 associates with the PM, and (3) PCYT1A was reportedly found on LDs in some cell types (Krahmer et al., 2011), we tested whether Pct1 can transiently exit the nucleus and associate with other membranes. To address this, we performed FLIP (fluorescence loss in

photobleaching) analyses with repeated bleaching of either an intranuclear or cytoplasmic region of cells expressing either GFP or Pct1-GFP. Repeated intranuclear photobleaching of cells expressing GFP alone reduced the cytosolic GFP signal (Figure 4A), and photobleaching of a similar size region of the cytoplasm led to a more gradual reduction in the nuclear GFP signal (Figure 4B). In contrast, in cells expressing Pct1-GFP, repeated photobleaching of the cytosol did not alter the nuclear GFP signal (Figure 4C), suggesting that, in the conditions tested, wild-type (WT) Pct1 remains inside the nucleus.

Pct1-GFP Membrane Localization Does Not Depend on the Source of PC Synthesis

PC can be exclusively synthesized from the CDP-choline pathway in a *cho2Δopi3Δ* double mutant supplemented with choline (Boumann et al., 2006, also see Figure 1A). To study *in vivo* regulation of Pct1 as the sole enzymatic source for PC, we generated a triple deletion mutant *cho2Δopi3Δpct1Δ* (henceforth called 3Δ mutant; Figure S5A), complemented by Opi3, which can partially compensate for the loss of Cho2 (Thibault et al., 2012). The Ura3 marker on the Opi3 expression plasmid converts 5-fluoroorotic acid (5-FOA) into a toxic product, preventing cell growth unless the cells are able to lose the *URA3* plasmid in the presence of another source of PC, such as a *PCT1* expression plasmid in the presence of choline. Indeed, both Pct1 and Pct1-GFP can rescue the growth of the 3Δ cells in the presence of 5-FOA (Figures S5B and S5C). We found that 3Δ cells show reduced viability when expressing Pct1 mutants designed to significantly impair membrane binding (Mdel) or catalytic activity (Cmut) (Figures S5B and S5C). As expected, the hypomorph Pct1-Mdel-GFP shows reduced association with the nuclear membrane under conditions that promote membrane association of the WT enzyme, i.e., without choline, whereas the Pct1-Cmut-GFP mutant remains membrane associated in conditions that usually promote membrane release, i.e., on choline supplementation (Figure S5D). In keeping with their perturbed nuclear envelope association, lipid analysis suggests that neither mutant was able to restore PC levels to the same extent as WT Pct1, although both appeared to retain some catalytic activity (Figure S5E). We also generated a mutant in which 34 amino acids within the core of the catalytic domain were deleted (Cdel mutant). As expected this mutant failed to rescue the 3Δ strain regardless of the presence of choline (Figure S5C).

Next, we sought to determine how membrane PL composition controls Pct1 localization *in vivo*. Previous studies have suggested that the fatty acid composition of the PC derived from the Kennedy pathway is different from the PC produced through methylation of PE (Boumann et al., 2003). Therefore, we first asked whether Pct1 membrane association depends on the source of PC. In the *cho2Δopi3Δ* cells expressing Pct1, PC is produced exclusively from the Kennedy pathway and Pct1-GFP

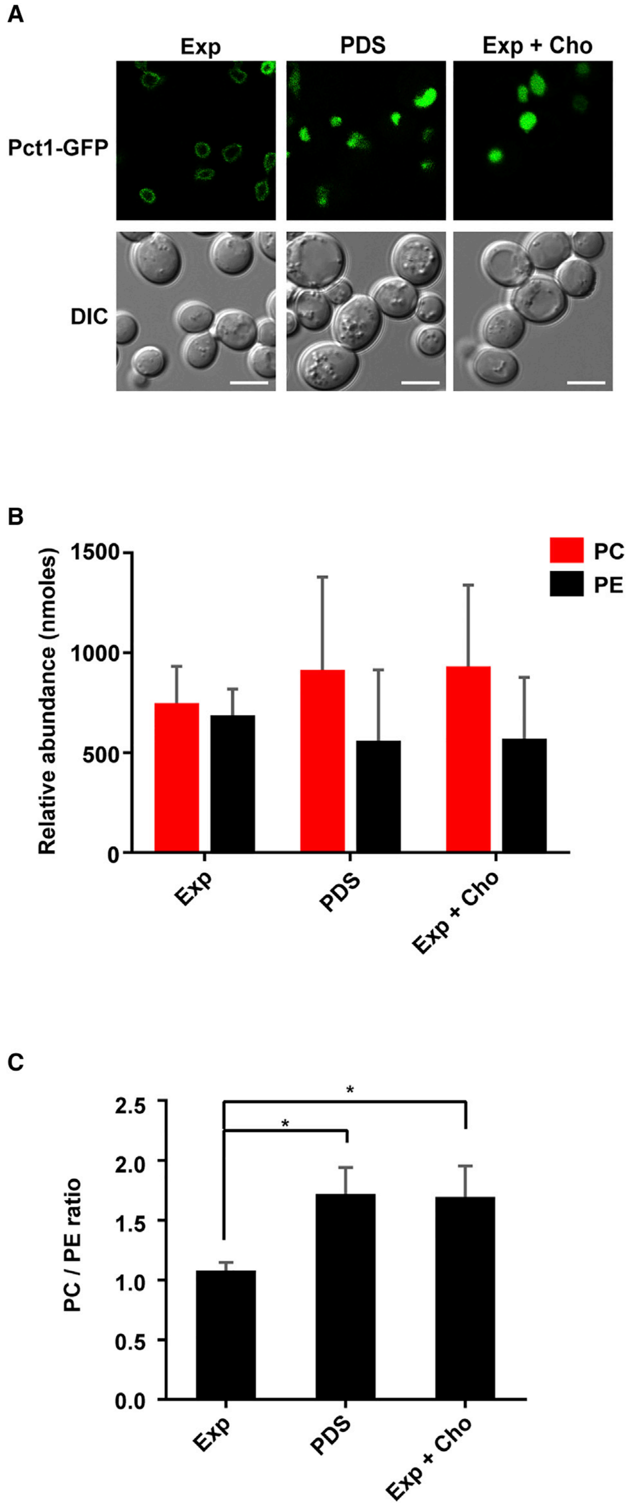

Figure 3. Binding of Pct1-GFP to the Nuclear Membrane Is Associated with PC Levels in Yeast

(A) *pct1Δ* cells expressing C-terminal GFP-tagged Pct1 were grown to exponential phase (Exp), post-diauxic shift (PDS) phase, or to exponential phase supplemented with 1 mM choline (Exp + Cho), and imaged as described in the STAR Methods. Differential interference contrast (DIC) images are shown for context. Scale bars, 5 μm.

(B) Lipidomic analysis of PC and PE levels at the indicated growth phase. The PE and PC levels shown in nmol (per 50 mg yeast) are relative to internal standards.

(C) PC/PE ratio of WT cells at the indicated growth phase.

Data are means ± SD from three independent experiments. One-way ANOVA with Bonferroni multiple comparison *$p < 0.05$. See Figures S3 and S4.

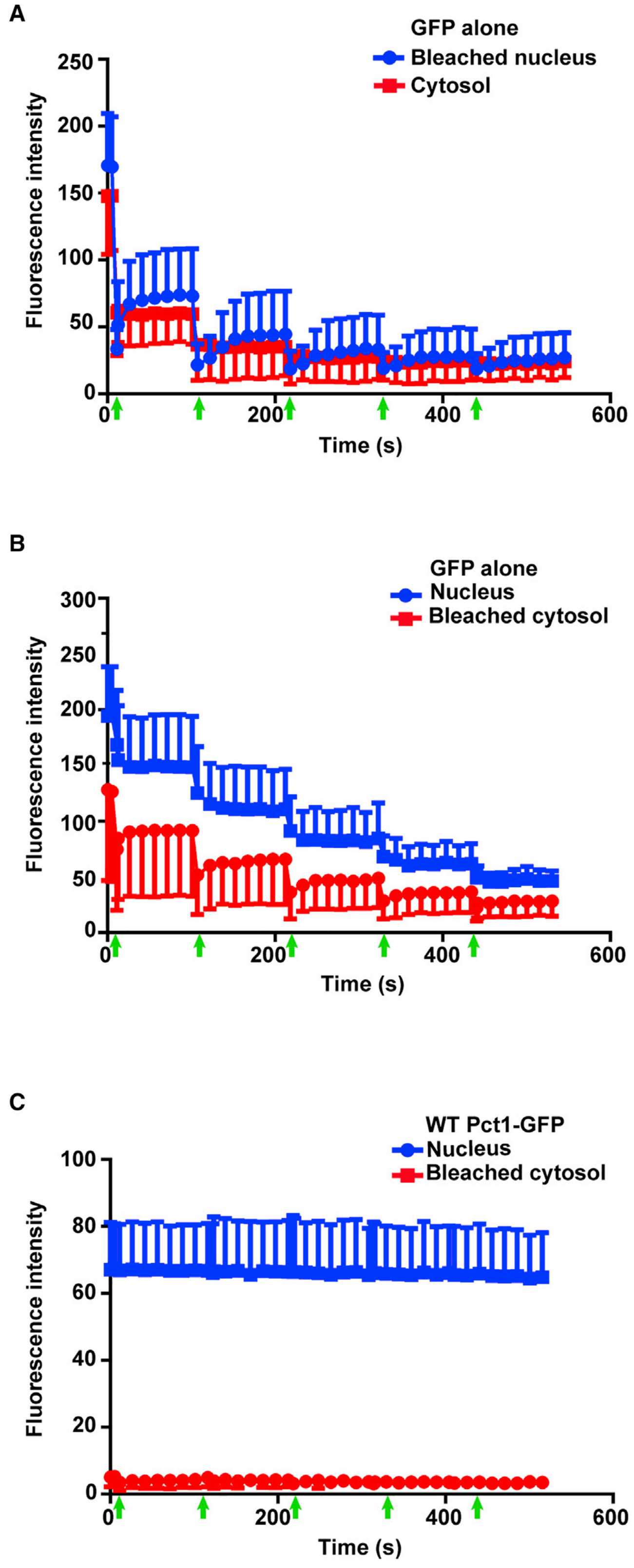

Figure 4. Pct1-GFP Is Confined to the Nucleus in Yeast Cells

(A and B) In WT (BY4742) yeast cells expressing GFP alone, repeated photobleaching of GFP in the nucleus (A) or cytosol (B) is rapidly followed by partial recovery of the fluorescence in the photobleached regions, with a concomitant progressive loss of fluorescence in the unbleached regions, indicating rapid movement of GFP between these two cellular compartments.

(C) In *pct1Δ* cells expressing WT Pct1-GFP, repeated photobleaching of the signal in the cytosol does not result in a loss of fluorescence of WT Pct1-GFP within the nucleus, indicating little or no rapid shuttling of Pct1-GFP from the nucleus to the cytosol. Data are means ± SD from three independent experiments (five to eight cells each). Arrows on the x axes indicate each bleaching event.

associates with the nuclear membrane in the absence of choline, reflecting cellular deficiency of PC, but shows a gradual nucleoplasmic re-localization when the cells are supplemented with choline (Figure S6). To force the cells to synthesize PC exclusively through the methylation of PE, we used a double mutant deleted for *CPT1* and *EPT1* (Figure S6A), which encode terminal enzymes of the two branches of the Kennedy pathway. Like in WT cells without choline supplementation, Pct1-GFP associates with the nuclear membrane during the exponential phase and falls off into the nucleoplasm during the PDS phase in the *cpt1Δept1Δ* double mutant (Figure S6). Therefore, Pct1 membrane association/dissociation occurs regardless of the source of PC synthesis.

Pct1-GFP Nuclear Membrane Association Correlates with the PC/PE Ratio

If Pct1 "senses" PC levels, its membrane binding must respond to specific changes in membrane properties *in vivo*. To address this hypothesis, we monitored how Pct1-GFP responds to choline addition, and the resulting PL changes in 3Δ cells in time course experiments. Although 3Δ cells expressing Pct1-GFP are viable, cell growth is arrested if the cells are deprived of choline (Boumann et al., 2006). However, cell growth can be restored by adding back choline after 24 hr of deprivation. Under choline starvation, Pct1-GFP is mostly bound to the nuclear membrane and PC levels are very low (Figure 5; 0 hr). Addition of choline to these cells induces a decrease of membrane-associated Pct1-GFP (Figures 5A and 5B) that correlates with rising PC levels and a significant increase in the PC/PE ratio (Figures 5C and 5D). We also observed an increase in LD size in these cells when deprived of choline (Figure 5E), which is consistent with previous data showing that PC deficiency induces super-sized LDs (Fei et al., 2011). As the cells generate PC, after choline supplementation, the LDs shrink in size (Figure 5E). Note that even in the choline-deprived state we do not detect Pct1-GFP around LDs, again suggesting that it is not trafficking in and out of the nucleus as if it was doing so, it would be expected to bind to the LDs as is the case with the Pct1-NLSm1 mutant (as discussed in the next section).

NLS Mutants of Pct1 and CCT1 Can Compensate for the Loss of Nuclear Pct1/CCT1 *In Vivo*

Why Pct1, CCT1, or PCYT1A sense and regulate PL levels within the nucleus remains unclear. To determine if this is an essential element in the activity of this pathway, we performed choline rescue experiments in 3Δ cells expressing Pct1-NLSm1-GFP. Notably, prolonged absence of choline leads to translocation of almost all Pct1-NLSm1-GFP to LDs (Figures 6A and 6B; 0 hr). Presumably, exclusion from the nucleus facilitates the LD

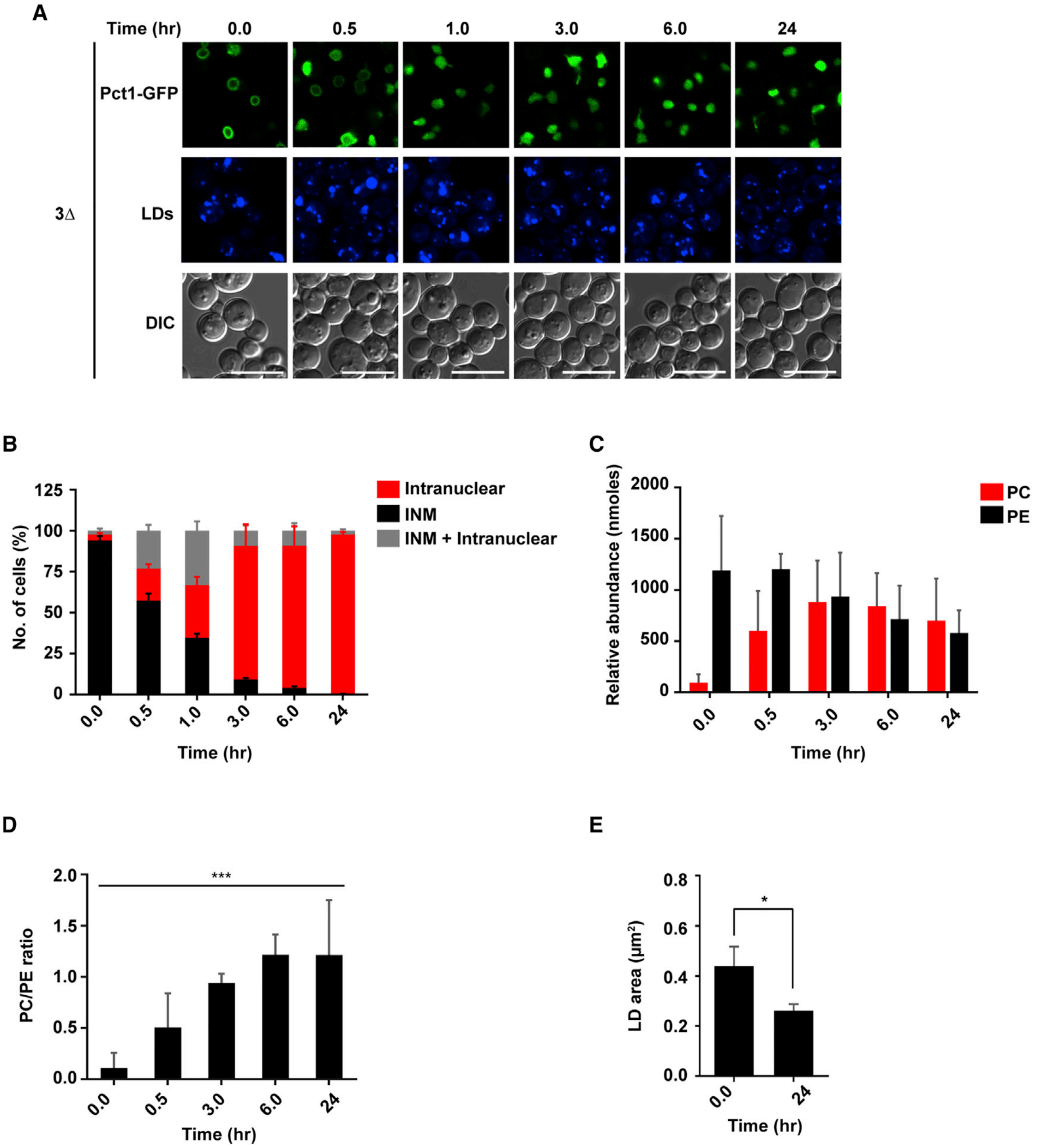

Figure 5. Changes in the PC/PE Ratio Correspond to Choline-Induced Pct1-GFP Translocation off the Nuclear Membrane

3Δ yeast cells expressing Pct1-GFP were grown for 24 hr followed by addition of 1 mM choline. Cells were collected before (0.0 hr) and after choline supplementation at the indicated time points for imaging or lipidomics analysis.

(A) Representative confocal microscopy images of Pct1-GFP localization. LDs were stained as described in the STAR Methods. DIC images are shown for context. Scale bars, 10 μm.

(B) Quantification of the Pct1-GFP localization shown in (A). INM, Inner nuclear membrane. Data are means ± SD from three independent experiments; ~300 cells were counted from random fields at each time point per experiment.

(C) PC and PE levels at the indicated time points are shown in nmol (per 50 mg yeast) relative to internal standards. Data represent the means ± SD from three independent experiments.

(D) PC/PE ratio at the indicated time points. Data are means ± SD. One-way ANOVA, ***$p < 0.001$.

(E) Measurement of LD area as described in the STAR Methods section from cells imaged in (A). Data are means ± SD from three independent experiments; ~ 600 LDs were counted in each condition. Two-tailed Student's t test, *$p < 0.05$.

See Figures S5 and S6.

localization of this mutant under PC-deficient conditions. Choline supplementation leads to a gradual increase of PC levels and the PC/PE ratio, and decrease of LD size in this mutant (Figures 6C–6E), with similar kinetics to the WT. Pct1-NLSm1-GFP also restores growth of the 3Δ cells to a similar extent to WT Pct1-GFP (Figure 6F).

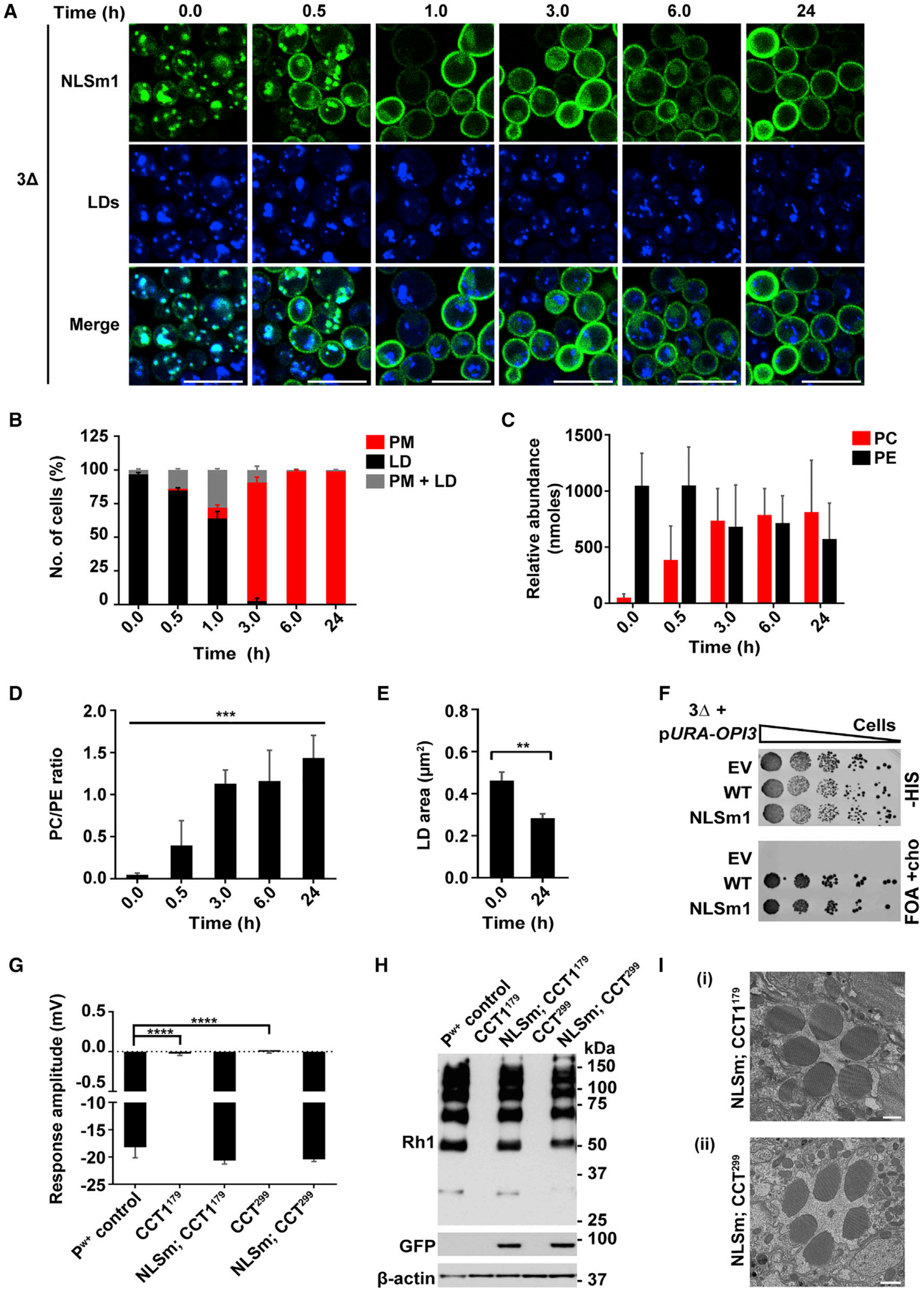

Figure 6. CCT NLS Mutants Can Compensate for the Loss of WT CCT in Yeast and in *Drosophila* PRs

(A–F) 3Δ yeast cells expressing Pct1-NLSm1-GFP were grown for 24 hr followed by addition of 1 mM choline. Cells were collected before (0.0 hr) and after choline supplementation at the indicated time points for imaging or lipidomics analysis.

(legend continued on next page)

These data suggest that Pct1 can regulate PC synthesis from within the nucleus or in the cytoplasm. To evaluate this in a more complex metazoan cell type, we generated a NLS mutant of the *Drosophila* CCT1 (CCT1-NLSm-GFP) (Figure S7A). In *Drosophila* S2 cells, while the WT CCT1 is nuclear, CCT1-NLSm is cytoplasmic and unlike Pct1-NLSm1 does not localize to the PM (Figure S7Ai). CCT1-NLSm also localizes to the periphery of LDs in oleate-loaded S2 cells (Figure S7Ai). When expressed in fly eyes under the *Rhodopsin 1 (Rh1)* promoter, this mutant protein was predominantly cytoplasmic, although we cannot entirely exclude the possibility that some of the protein was intranuclear (Figure S7Aii). The phenotype of *CCT1* null eyes in mosaic flies was rescued by transgenic expression of CCT1-NLSm-GFP (Figures 6G and 6H). Transmission electron micrographs of retinal cross-sections from these rescued flies showed normal rhabdomere morphology, notably only in six of the eight PRs within each ommatidium as the expression of the transgenic rescue construct is restricted to PRs 1–6 by the *Rh1* promoter (Figure 6I). These results corroborate our findings from the yeast model suggesting that nuclear compartmentalization of CCT1 is not essential for its function. Nevertheless, they do not entirely preclude the possibility that Pct1/CCT1/PCYT1A has other important functions which require it to be compartmentalized inside the nucleus.

Evidence of SCE Stress Regulation

The biophysical properties of membranes are influenced by both fatty acid (FA) and head group changes in the PLs. Thus, mass spectrometry analysis was used to comprehensively characterize the yeast lipidome in 3Δ cells expressing WT-Pct1-GFP or NLSm1-Pct1-GFP in choline-deprived conditions (at 0 hr), and at different time points following choline supplementation, aligned with the data in Figures 5 and 6. These data show that cells compensate for PC depletion (at 0 hr) by significantly increasing PE and also by small but significant decreases in PS lipid classes, whereas the amounts of other lipid classes present in biological membranes are unchanged (Figure 7A). As indicated in Figure 1A, PE and PC can be synthesized in WT yeast by both the Kennedy pathway and from PS via the CDP-DAG pathway, with the latter pathway becoming increasingly important in choline-deprived cells. This is driven mainly by the ER-localized enzyme PS synthase, Cho1 in yeast, which converts CDP-DAG into PS. The PS is then decarboxylated by PS decarboxylases, Psd1 and Psd2, to form PE (Han and Carman, 2017). PE in turn can be converted to PC via the methylation pathway. In the choline starved state, we hypothesize that cells may increase decarboxylation of PS to PE to drive the CDP-DAG pathway, thus reducing PS levels (Figure 7A); however, with the methylation pathway blocked by the deletion of Cho2 Opi3 enzymes, PE levels will tend to rise as seen in Figure 7A. TAG levels were higher without choline and fell on choline supplementation (Figure 7A) concomitant with the reduction in LD size observed in Figures 5 and 6. Changes in PC, PE, and PS lipid fractions coincide with adaptive changes in the FA acylation patterns of these lipids (Figures 7B–7D). Notably, PE 32:1 and PS 32:1 were increased to ~30 mol% of total PE or PS (Figures 7C and 7D) at time 0 hr. After choline supplementation (at 24 hr), these elevated FA species decreased and PE 34:2 and PS 34:2 rose to compensate. Thus, 3Δ cells expressing WT Pct1 compensate for the low PC and elevated PE by replacing monounsaturated FAs with saturated FAs predominantly in the PE lipids.

As PC and PE are the dominant PLs in yeast and most other cell membranes, the sizable changes in PC/PE ratios that we observed (Figures 5D and 6D) are likely to significantly impact membrane SCE stress. Accumulation of PE, a conically shaped lipid, among other more cylindrically shaped PLs such as PC, will increase the number of membrane-packing defects and in turn increase membrane SCE stress. To explore this in more detail we estimated the metric P_{SCE} for SCE stress regulation *in vivo*, derived from data-driven modeling studies (Dymond, 2016). This provides a metric for total cellular SCE stress including both protein and lipid contributions, by using the lipidomic data alongside parameters (w values, Equation 3; STAR methods) that are derived from previous studies of cells regulating their SCE under different culture conditions. In 3Δ cells expressing either WT-Pct1-GFP or Pct1-NSLm1-GFP, we found a narrow range of P_{SCE} values from 0.1 to 0.2 (Figure 7E), which is evidence of preservation of membrane SCE stress throughout.

(A) Representative confocal microscopy images of Pct1-NLSm1 localization. LDs were stained as described in the STAR Methods. Merged (Merge) fluorescent channels show co-localization. Scale bars, 10 μm.

(B) Quantification of the Pct1-NLSm1-GFP localization shown in (A); PM, plasma membrane; LD, Lipid droplet. Data are means ± SD based on random fields from three independent experiments; ~300 cells were counted at each time point per experiment.

(C) PC and PE levels at the indicated time points shown in nmol (per 50 mg yeast) are relative to internal standards. Data represent the means ± SD based on measurement from three independent experiments.

(D) PC/PE ratio at the indicated time points from data in (C). Data are means ± SD. One-way ANOVA, ***$p < 0.001$.

(E) Measurement of LD area from cells imaged in (A) as described in the STAR Methods. Data are means ± SD from three independent experiments; ~600 LDs were counted per condition. Two-tailed Student's t test, **$p < 0.01$.

(F) 3Δ cells carrying Ycplac33-*URA3-OPI3* were transformed with *CEN/HIS3* plasmids expressing either WT Pct1, NLSm1, or the corresponding empty plasmid (EV). Cells were grown to exponential phase and serial dilution of liquid cultures were spotted onto plates lacking histidine (-HIS) or plates supplemented with 5-FOA.

(G–I) The "blind" phenotype of the $CCT1^{179}$ and CCT^{299} homozygous eyes was rescued by transgenic retinal expression of a CCT1-NLSm-GFP mutant, CCT1-NLSm; $CCT1^{179}$ and CCT1-NLSm; CCT^{299}.(G) Response to light activation was assessed by ERG scans and the mean response generated at the maximal intensity was plotted for four to six flies of each genotype. Flies with *CCT1* null eyes are completely blind even at the maximum intensity, while the CCT1-NLSm-GFP expression restores the response to WT levels. Data are reported as means ± SD. One-way ANOVA with Bonferroni multiple comparison, ****$p < 0.0001$. (H) The CCT1-NLSm-GFP mutant restores the rhodopsin level in flies with *CCT1* null eyes ($CCT1^{179}$ or CCT^{299}) back to the control levels. (I) Transmission electron micrographs of retinal tissue sections from flies with *CCT1* null eyes rescued by transgenic expression of CCT1-NLSm-GFP mutant, CCT1-NLSm; $CCT1^{179}$ (i) and CCT1-NLSm; CCT^{299} (ii). *Rhodopsin* (*Rh1*) promoter-driven expression of CCT1-NLSm allows normal development of rhabdomere stacks in the PRs 1–6. Scale bars, 500 nm.

See Figure S7.

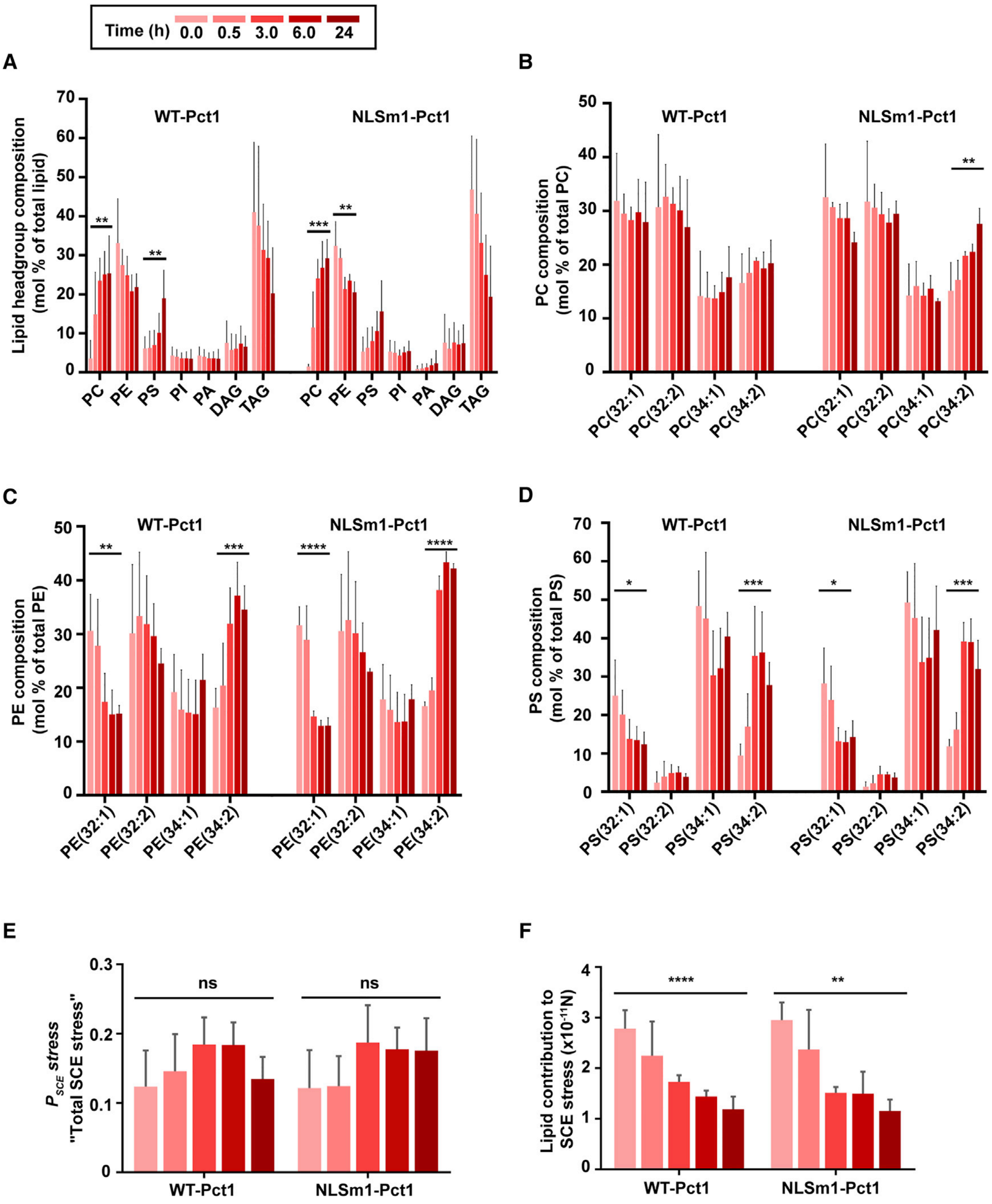

Figure 7. Data-Driven Modeling of SCE Stress Regulation from the Lipidomes of 3Δ Cells Expressing WT Pct1-GFP or NLSm1-Pct1-GFP and Undergoing PC Recovery

(A–F) 3Δ yeast cells expressing either WT Pct1-GFP or Pct1-NLSm1-GFP were grown for 24 hr followed by addition of 1 mM choline. Cells were collected before (0.0 hr) and after choline supplementation at the indicated time points (see top left inset for time point color codes) for lipidomics analyses and the total (P_{SCE}) and lipid-driven SCE stress in the cells was calculated as described in the STAR Methods. These data correlate with the imaging slides shown in Figures 5 and 6.

(A) Lipid composition by head group (mol% total lipid). PC, phosphatidylcholine; PE, phospatidylethanolamine; PS, phosphatidylserine; PI, phosphatidylinositol; PA, phosphatidic acid; DAG, diacylglycerol; TAG, triacylglycerol.

(B–D) FA composition of the PC (A), PE (B), and PS (C) fraction expressed as mol% of the respective total PL. The PL (32:1) or (34:1) represent species where the acyl groups at C-1 and C-2 contain a total of 32 or 34 carbon atoms with one double bond, while the PL species (32:2) or (34:2) represent species with two double bonds.

(E) Estimates of the total SCE stress (*P_{SCE} stress*).

(F) Estimates of the lipid contribution to SCE stress.

All data are means ± SD of three to four experimental repeats. One-way ANOVA, *$p < 0.05$, **$p < 0.01$, ***$p < 0.001$, ****$p < 0.0001$. See Figure S7.

Mechanistically, cells can control SCE stress in their membranes by changing lipid composition or by partitioning proteins, such as Pct1, which relieve SCE stress to the membrane. So, to evaluate the lipid-specific contribution to SCE stress in membranes we next calculated a purely lipid-based metric of SCE stress (Figure 7). This is different to P_{SCE}, which, albeit in a coarse-grained way, is influenced by both membrane protein and lipid contributions to overall membrane SCE stress. In 3Δ cells expressing WT Pct1, the lipid contribution to membrane SCE stress is high, in the order of 2.8×10^{-11} N, when PC levels are depleted which correlates well with the localization of Pct1 on the nuclear envelope (Figure 7F). These estimates suggest that changes in lipid FA acylation do not fully compensate for the increase in PE that results from PC deficiency (at earlier time points), and that Pct1 is recruited to the nuclear membrane to alleviate the remaining SCE stress. The fact that the Pct1 catalytic domain mutant (Cmut) remains on the NE following choline supplementation (Figure S5D), but is still unable to fully restore lipid-based estimates of SCE stress (Figure S7B) is consistent with this suggestion. The Mdel Pct1 mutant was also unable to fully normalize lipid-derived SCE stress estimates (Figure S7B).

The data suggest that equivalent processes regulate SCE stress in 3Δ cells expressing NSLm1-Pct1-GFP (Figures 7A–7F), since similar behavior is observed in terms of lipidomic changes. When PC levels fall significantly, the surface tension on LD is expected to rise concomitantly, resulting in LD fusion and growth (Guo et al., 2008), as well as Pct1-GFP localization to the LD. Restoration of PC levels presumably restores LD surface tension allowing Pct1-NLSm1-GFP to fall off the droplet. Interestingly, in this setting Pct1-NLSm1 relocates to the PM as well as the cytosol (Figure 6). This result may be due to the fact that the membrane binding domain of Pct1 is enriched with positively charged amino acids, and this may facilitate its interaction with PS, a PL with a net negative charge that is enriched at the inner leaflet of the PM (Daleke, 2003) and the levels of which rise following ‘‘choline rescue’’ (Figure 7A).

DISCUSSION

Although PC synthesis and turnover are critical for cell growth and organelle function, how cells sense and adjust their membrane PC content *in vivo* remains poorly understood. Our data suggest that PCYT1A is largely (and probably entirely in most cell types) confined to the nucleus where it uses a nascent amphipathic helix to detect membrane packing defects generated mainly by the presence of glycerolipids with small uncharged headgroups, primarily PE and DAG, rather than PC itself. The membrane binding domain of PCYT1A then folds into an amphipathic helix on the membrane, thereby activating it and thus catalyzing the synthesis of CDP-choline, a soluble product, which is small enough to diffuse throughout or even out of the nucleus, where it can be used by the terminal enzyme responsible for catalyzing the addition of a phosphocholine head group to DAG. By associating with the membrane, the amphipathic helix of PCYT1A temporarily alleviates membrane SCE until sufficient PC is generated to stabilize the membrane. Under normal circumstances, DAG levels in membranes are very low, presumably at least in part as their highly conical shape has a pronounced effect on membrane SCE stress, and the model we are proposing would also provide a defense mechanism against excessive DAG accumulation by triggering its conversion into PC instead. This process is rate-limiting for Kennedy pathway PC synthesis and provides an exquisite membrane topological sensing mechanism which operates co-operatively with transmembrane sensors such as Mga2 and Sre1, primarily involved in detecting the intramembranous fatty acyl chain environment. By sensing membrane properties like fluidity and SCE stress, which impact the activities of some membrane proteins and feedback into transcriptional regulation, cells can maintain these dynamic processes within relatively tight boundaries under conditions when PL composition is outside ‘‘normal’’ values.

Using three evolutionarily disparate model systems, we show that PCYT1A associates with the nuclear envelope in response to physiological signals for membrane biogenesis in several tissues. To explain why PCYT1A is an intranuclear enzyme, we envision two possibilities; first, Kennedy-derived PC may be compartmentalized if the terminal enzyme of the pathway, choline phosphotransferase (CPT), is also active at the inner nuclear membrane. In mammals, two enzymes can generate PC from CDP-choline: CPT1, which has been localized to the Golgi; and choline/ethanolamine phosphotransferase 1 (CEPT1), which localizes to the ER and nuclear envelope; a pool of CEPT1 translocates to the nuclear membrane and co-localizes with PCYT1A in response to exogenous activation of PC synthesis by FA supplementation (Henneberry et al., 2002). Notably, lipins, which generate DAG required for the terminal step of PC synthesis, also display a nuclear pool from yeast cells to mammals (Siniossoglou, 2013). Since the nuclear membrane is continuous with the ER, PC could then be distributed to the endomembrane system through both vesicular and non-vesicular pathways. Alternatively, since CDP-choline is water soluble, PC synthesis could also occur in the cytoplasmic ER network. Accordingly, ‘‘sensing’’ of membrane-packing defects would be spatially separated from the site of PC synthesis that restores them. In this scenario, the presence of PCYT1A in the nucleus could couple its sensing function to another nuclear role, such as transcriptional regulation of genes encoding PC biosynthetic enzymes or nuclear envelope remodeling activity, which leads to the formation of the nucleoplasmic reticulum (Lagace and Ridgway, 2005).

In most cell types, there is a second cytosolic PCYT1B isoform that presumably senses changes in the ER and possibly other membrane bound organelles. However, its expression appears to be very low in many cell types (Marcucci et al., 2008; Karim et al., 2003), and *Pcyt1b* null mice manifest subtle phenotypes including axonal branching defects in sympathetic neurons and reproductive insufficiency mainly resulting due to defective ovarian follicles and spermatogenesis (Strakova et al., 2011; Jackowski et al., 2004), whereas *Pcyt1a* null mice die *in utero* (Wang et al., 2005). The fact that the nuclear envelope is continuous with the ER could presumably facilitate PL diffusion from within the nucleus to the peripheral ER and vice versa, which might also account for our observations suggesting that NLS mutants of Pct1 in yeast and CCT1 in flies can compensate for the absence of WT enzyme. These findings are also supported by the ability of CCT2, at least when overexpressed, to rescue rhabdomere formation in *CCT1/2* knockout PRs (data not shown). However, since a pool of the Pct1-NLSm1 mutant can still be detected in the nucleus (Figures 6A, S4B, and S4C), it is

possible that lower levels of nuclear Pct1 can still rescue the 3Δ cells, but a mutant in which most of the amino acids comprising the NLS of Pct1 has been deleted, Pct1-NLS1del, was still able to rescue the 3Δ phenotype (Figure S7C), and FLIP analysis does not suggest that significant amounts of Pct1-NLSm1 traffic into the nucleus anyway (Figure S7D). A better understanding of the signals that govern nucleocytoplasmic trafficking of Pct1 will be needed to fully elucidate this issue.

In summary, we propose a model in which PC synthesis is primarily regulated by intranuclear PCYT1A that senses changes in the surface topology of the inner nuclear membrane. This model (1) is broadly consistent with a substantial body of *in vitro* work characterizing the structural and biochemical properties of this enzyme (Cornell, 2016), and (2) augments recent progress in understanding how cells sense and regulate the internal membrane environment.

STAR★METHODS

Detailed methods are provided in the online version of this paper and include the following:

- KEY RESOURCES TABLE
- CONTACT FOR REAGENT AND RESOURCE SHARING
- EXPERIMENTAL MODEL AND SUBJECT DETAILS
  - Animal Models
  - Eukaryotic Cell Lines and Primary Culture
  - Fly Stocks
  - Yeast Strains, Plasmids and Culture
- METHOD DETAILS
  - Immunohistochemistry
  - Immunostaining
  - Confocal Microscopy of Dissociated Ommatidia and S2 Cells
  - Electroretinograms (ERGs)
  - Immunoblotting
  - Electron Micrographs
  - Yeast Confocal Microscopy, Image Analysis and Quantifications
  - FLIP Assay
  - Yeast Lipid Profiling
  - Estimates of the Lipid Contribution to Membrane Stored Curvature Elastic (SCE) Stress
  - Estimate of the Total Membrane Stored Curvature Elastic (SCE) Stress of Cells (Referred to Herein as P_{SCE})
- QUANTIFICATION AND STATISTICAL ANALYSIS

SUPPLEMENTAL INFORMATION

Supplemental Information includes seven figures and can be found with this article online at https://doi.org/10.1016/j.devcel.2018.04.012.

ACKNOWLEDGMENTS

The authors thank Prof. Paul Luzio for helpful discussions and Prof. Graham R. Williams and Prof. J.H. Duncan Bassett (Imperial College London, UK) for providing mouse cartilage sections. We also thank Prof. Tobias Walther (Broad Institute of MIT and Harvard) for sharing *CCT1* and *CCT2* cDNA clones. The Department of Genetics Fly Facility and the Cambridge Advanced Imaging Center, at the University of Cambridge, are acknowledged for providing fly breeding/microinjection and TEM services, respectively. S.S is supported by a Wellcome Trust Seed award (108042) and the Wellcome Trust Strategic award (100140) and equipment grant (093026) to the Cambridge Institute for Medical Research. D.B.S is supported by the Wellcome Trust (WT 107064), the MRC Metabolic Disease Unit, and the National Institute for Health Research (NIHR) Cambridge Biomedical Research Center and NIHR Rare Disease Translational Research Collaboration.

AUTHOR CONTRIBUTIONS

Conceptualization, D.B.S. and S.S., with help from V.S., M.K.D., and R.C.H. Experimental investigation, A.H., Y.-C.W., K.L., A.D.B., and L.D. M.M.H. provided liver samples from *Mtp* knockout mice. S.P. and A.A.-G. provided support in experimental design and setup. K.O., S.C., M.M., and U.W. generated and maintained fly stocks, and R.C.H. did all ERG recordings. R.C.H., U.W., M.M., S.C., and C.-H.L. helped design fly experiments. B.J.J. and A.K. performed the lipidomic analysis, and M.K.D. carried out mathematical modeling of the lipidomic data. A.H., Y.-C.W., K.L., L.D., A.D.B., C.-H.L., U.W., M.K.D., R.C.H., S.S., and D.B.S. analyzed data. A.H., Y.-C.W., K.L., A.D.B., C.-H.L., U.W., M.K.D., S.S., and D.B.S. wrote the paper, which was reviewed and edited by all the authors.

DECLARATION OF INTERESTS

The authors declare no competing interests.

Received: October 30, 2017
Revised: February 27, 2018
Accepted: April 11, 2018
Published: May 10, 2018

REFERENCES

Aitchison, A.J., Arsenault, D.J., and Ridgway, N.D. (2015). Nuclear-localized CTP:phosphocholine cytidylyltransferase alpha regulates phosphatidylcholine synthesis required for lipid droplet biogenesis. Mol. Biol. Cell *26*, 2927–2938.

Antonny, B. (2011). Mechanisms of membrane curvature sensing. Annu. Rev. Biochem. *80*, 101–123.

Arnold, R.S., and Cornell, R.B. (1996). Lipid regulation of CTP: phosphocholine cytidylyltransferase: electrostatic, hydrophobic, and synergistic interactions of anionic phospholipids and diacylglycerol. Biochemistry *35*, 9917–9924.

Attard, G.S., Templer, R.H., Smith, W.S., Hunt, A.N., and Jackowski, S. (2000). Modulation of CTP:phosphocholine cytidylyltransferase by membrane curvature elastic stress. Proc. Natl. Acad. Sci. USA *97*, 9032–9036.

Barbosa, A.D., Sembongi, H., Su, W.M., Abreu, S., Reggiori, F., Carman, G.M., and Siniossoglou, S. (2015). Lipid partitioning at the nuclear envelope controls membrane biogenesis. Mol. Biol. Cell *26*, 3641–3657.

Bigay, J., Casella, J.F., Drin, G., Mesmin, B., and Antonny, B. (2005). ArfGAP1 responds to membrane curvature through the folding of a lipid packing sensor motif. EMBO J. *24*, 2244–2253.

Boumann, H.A., Damen, M.J., Versluis, C., Heck, A.J., de Kruijff, B., and de Kroon, A.I. (2003). The two biosynthetic routes leading to phosphatidylcholine in yeast produce different sets of molecular species. Evidence for lipid remodeling. Biochemistry *42*, 3054–3059.

Boumann, H.A., Gubbens, J., Koorengevel, M.C., Oh, C.S., Martin, C.E., Heck, A.J., Patton-Vogt, J., Henry, S.A., de Kruijff, B., and de Kroon, A.I. (2006). Depletion of phosphatidylcholine in yeast induces shortening and increased saturation of the lipid acyl chains: evidence for regulation of intrinsic membrane curvature in a eukaryote. Mol. Biol. Cell *17*, 1006–1017.

Brighton, C.T., Sugioka, Y., and Hunt, R.M. (1973). Cytoplasmic structures of epiphyseal plate chondrocytes. Quantitative evaluation using electron micrographs of rat costochondral junctions with special reference to the fate of hypertrophic cells. J. Bone Joint Surg. Am. *55*, 771–784.

Brown, M.S., Radhakrishnan, A., and Goldstein, J.L. (2017). Retrospective on cholesterol homeostasis: the central role of scap. Annu. Rev. Biochem. https://doi.org/10.1146/annurev-biochem-062917-011852.

Cole, L.K., Vance, J.E., and Vance, D.E. (2012). Phosphatidylcholine biosynthesis and lipoprotein metabolism. Biochim. Biophys. Acta *1821*, 754–761.

Colley, N.J., Cassill, J.A., Baker, E.K., and Zuker, C.S. (1995). Defective intracellular transport is the molecular basis of rhodopsin-dependent dominant retinal degeneration. Proc. Natl. Acad. Sci. USA *92*, 3070–3074.

Cornell, R.B. (2016). Membrane lipid compositional sensing by the inducible amphipathic helix of CCT. Biochim. Biophys. Acta *1861*, 847–861.

Cornell, R.B., and Ridgway, N.D. (2015). CTP:phosphocholine cytidylyltransferase: function, regulation, and structure of an amphitropic enzyme required for membrane biogenesis. Prog. Lipid Res. *59*, 147–171.

Covino, R., Ballweg, S., Stordeur, C., Michaelis, J.B., Puth, K., Wernig, F., Bahrami, A., Ernst, A.M., Hummer, G., and Ernst, R. (2016). A eukaryotic sensor for membrane lipid saturation. Mol. Cell *63*, 49–59.

Daleke, D.L. (2003). Regulation of transbilayer plasma membrane phospholipid asymmetry. J. Lipid Res. *44*, 233–242.

Davies, S.M., Epand, R.M., Kraayenhof, R., and Cornell, R.B. (2001). Regulation of CTP: phosphocholine cytidylyltransferase activity by the physical properties of lipid membranes: an important role for stored curvature strain energy. Biochemistry *40*, 10522–10531.

Ding, J.D., Salinas, R.Y., and Arshavsky, V.Y. (2015). Discs of mammalian rod photoreceptors form through the membrane evagination mechanism. J. Cell Biol. *211*, 495–502.

Drin, G., Casella, J.F., Gautier, R., Boehmer, T., Schwartz, T.U., and Antonny, B. (2007). A general amphipathic alpha-helical motif for sensing membrane curvature. Nat. Struct. Mol. Biol. *14*, 138–146.

Dymond, M.K. (2016). Mammalian phospholipid homeostasis: evidence that membrane curvature elastic stress drives homeoviscous adaptation in vivo. J. R. Soc. Interface *13*, https://doi.org/10.1098/rsif.2016.0228.

Eberle, D., Hegarty, B., Bossard, P., Ferre, P., and Foufelle, F. (2004). SREBP transcription factors: master regulators of lipid homeostasis. Biochimie *86*, 839–848.

Ernst, R., Ejsing, C.S., and Antonny, B. (2016). Homeoviscous adaptation and the regulation of membrane lipids. J. Mol. Biol. *428*, 4776–4791.

Fagone, P., Sriburi, R., Ward-Chapman, C., Frank, M., Wang, J., Gunter, C., Brewer, J.W., and Jackowski, S. (2007). Phospholipid biosynthesis program underlying membrane expansion during B-lymphocyte differentiation. J. Biol. Chem. *282*, 7591–7605.

Fei, W., Zhong, L., Ta, M.T., Shui, G., Wenk, M.R., and Yang, H. (2011). The size and phospholipid composition of lipid droplets can influence their proteome. Biochem. Biophys. Res. Commun. *415*, 455–462.

Folch, J., Lees, M., and Sloane Stanley, G.H. (1957). A simple method for the isolation and purification of total lipides from animal tissues. J. Biol. Chem. *226*, 497–509.

Guo, Y., Walther, T.C., Rao, M., Stuurman, N., Goshima, G., Terayama, K., Wong, J.S., Vale, R.D., Walter, P., and Farese, R.V. (2008). Functional genomic screen reveals genes involved in lipid-droplet formation and utilization. Nature *453*, 657–661.

Gupta, T., and Schupbach, T. (2003). Cct1, a phosphatidylcholine biosynthesis enzyme, is required for *Drosophila* oogenesis and ovarian morphogenesis. Development *130*, 6075–6087.

Han, G., and Carman, G.M. (2017). Yeast PAH1-encoded phosphatidate phosphatase controls the expression of CHO1-encoded phosphatidylserine synthase for membrane phospholipid synthesis. J. Biol. Chem. *292*, 13230–13242.

Hardie, R.C. (1991). Voltage-sensitive potassium channels in *Drosophila* photoreceptors. J. Neurosci. *11*, 3079–3095.

Hardie, R.C., and Juusola, M. (2015). Phototransduction in *Drosophila*. Curr. Opin. Neurobiol. *34*, 37–45.

Hardie, R.C., Liu, C.H., Randall, A.S., and Sengupta, S. (2015). In vivo tracking of phosphoinositides in *Drosophila* photoreceptors. J. Cell Sci. *128*, 4328–4340.

Haruki, H., Nishikawa, J., and Laemmli, U.K. (2008). The anchor-away technique: rapid, conditional establishment of yeast mutant phenotypes. Mol. Cell *31*, 925–932.

Henneberry, A.L., Wright, M.M., and McMaster, C.R. (2002). The major sites of cellular phospholipid synthesis and molecular determinants of fatty acid and lipid head group specificity. Mol. Biol. Cell *13*, 3148–3161.

Hoover-Fong, J., Sobreira, N., Jurgens, J., Modaff, P., Blout, C., Moser, A., Kim, O.H., Cho, T.J., Cho, S.Y., Kim, S.J., et al. (2014). Mutations in PCYT1A, encoding a key regulator of phosphatidylcholine metabolism, cause spondylometaphyseal dysplasia with cone-rod dystrophy. Am. J. Hum. Genet. *94*, 105–112.

Jackowski, S., Rehg, J.E., Zhang, Y.M., Wang, J., Miller, K., Jackson, P., and Karim, M.A. (2004). Disruption of CCTbeta2 expression leads to gonadal dysfunction. Mol. Cell. Biol. *24*, 4720–4733.

Janke, C., Magiera, M.M., Rathfelder, N., Taxis, C., Reber, S., Maekawa, H., Moreno-Borchart, A., Doenges, G., Schwob, E., Schiebel, E., et al. (2004). A versatile toolbox for PCR-based tagging of yeast genes: new fluorescent proteins, more markers and promoter substitution cassettes. Yeast *21*, 947–962.

Karanasios, E., Han, G.S., Xu, Z., Carman, G.M., and Siniossoglou, S. (2010). A phosphorylation-regulated amphipathic helix controls the membrane translocation and function of the yeast phosphatidate phosphatase. Proc. Natl. Acad. Sci. USA *107*, 17539–17544.

Karim, M., Jackson, P., and Jackowski, S. (2003). Gene structure, expression and identification of a new CTP:phosphocholine cytidylyltransferase beta isoform. Biochim. Biophys. Acta *1633*, 1–12.

Khatun, I., Zeissig, S., Iqbal, J., Wang, M., Curiel, D., Shelness, G.S., Blumberg, R.S., and Hussain, M.M. (2012). Phospholipid transfer activity of microsomal triglyceride transfer protein produces apolipoprotein B and reduces hepatosteatosis while maintaining low plasma lipids in mice. Hepatology *55*, 1356–1368.

Koulman, A., Woffendin, G., Narayana, V.K., Welchman, H., Crone, C., and Volmer, D.A. (2009). High-resolution extracted ion chromatography, a new tool for metabolomics and lipidomics using a second-generation orbitrap mass spectrometer. Rapid Commun. Mass Spectrom. *23*, 1411–1418.

Krahmer, N., Guo, Y., Wilfling, F., Hilger, M., Lingrell, S., Heger, K., Newman, H.W., Schmidt-Supprian, M., Vance, D.E., Mann, M., et al. (2011). Phosphatidylcholine synthesis for lipid droplet expansion is mediated by localized activation of CTP:phosphocholine cytidylyltransferase. Cell Metab. *14*, 504–515.

Kuerschner, L., Moessinger, C., and Thiele, C. (2008). Imaging of lipid biosynthesis: how a neutral lipid enters lipid droplets. Traffic *9*, 338–352.

Kurada, P., and O'Tousa, J.E. (1995). Retinal degeneration caused by dominant rhodopsin mutations in *Drosophila*. Neuron *14*, 571–579.

Kwak, B.Y., Zhang, Y.M., Yun, M., Heath, R.J., Rock, C.O., Jackowski, S., and Park, H.W. (2002). Structure and mechanism of CTP:phosphocholine cytidylyltransferase (LicC) from *Streptococcus pneumoniae*. J. Biol. Chem. *277*, 4343–4350.

Laba, J.K., Steen, A., and Veenhoff, L.M. (2014). Traffic to the inner membrane of the nuclear envelope. Curr. Opin. Cell Biol. *28*, 36–45.

Lagace, T.A., and Ridgway, N.D. (2005). The rate-limiting enzyme in phosphatidylcholine synthesis regulates proliferation of the nucleoplasmic reticulum. Mol. Biol. Cell *16*, 1120–1130.

Lee, J., Johnson, J., Ding, Z., Paetzel, M., and Cornell, R.B. (2009). Crystal structure of a mammalian CTP: phosphocholine cytidylyltransferase catalytic domain reveals novel active site residues within a highly conserved nucleotidyltransferase fold. J. Biol. Chem. *284*, 33535–33548.

Longtine, M.S., McKenzie, A., 3rd, Demarini, D.J., Shah, N.G., Wach, A., Brachat, A., Philippsen, P., and Pringle, J.R. (1998). Additional modules for versatile and economical PCR-based gene deletion and modification in *Saccharomyces cerevisiae*. Yeast *14*, 953–961.

Lu, L., Koulman, A., Petry, C.J., Jenkins, B., Matthews, L., Hughes, I.A., Acerini, C.L., Ong, K.K., and Dunger, D.B. (2016). An unbiased lipidomics approach identifies early second trimester lipids predictive of maternal glycemic traits and gestational diabetes mellitus. Diabetes Care *39*, 2232–2239.

MacKinnon, M.A., Curwin, A.J., Gaspard, G.J., Suraci, A.B., Fernandez-Murray, J.P., and McMaster, C.R. (2009). The Kap60-Kap95 karyopherin complex directly regulates phosphatidylcholine synthesis. J. Biol. Chem. *284*, 7376–7384.

Magdeleine, M., Gautier, R., Gounon, P., Barelli, H., Vanni, S., and Antonny, B. (2016). A filter at the entrance of the Golgi that selects vesicles according to size and bulk lipid composition. Elife *5*, https://doi.org/10.7554/eLife.16988.

Manford, A.G., Stefan, C.J., Yuan, H.L., Macgurn, J.A., and Emr, S.D. (2012). ER-to-plasma membrane tethering proteins regulate cell signaling and ER morphology. Dev. Cell *23*, 1129–1140.

Marcucci, H., Elena, C., Gilardoni, P., and Banchio, C. (2008). Characterization of the murine CTP:phosphocholine cytidylyltransferase beta gene promoter. Biochim. Biophys. Acta *1781*, 254–262.

McDonald, C., Jovanovic, G., Ces, O., and Buck, M. (2015). Membrane stored curvature elastic stress modulates recruitment of maintenance proteins PspA and Vipp1. MBio *6*, e01188–15.

McMaster, C.R., and Bell, R.M. (1994). Phosphatidylcholine biosynthesis in *Saccharomyces cerevisiae*. Regulatory insights from studies employing null and chimeric sn-1,2-diacylglycerol choline- and ethanolaminephosphotransferases. J. Biol. Chem. *269*, 28010–28016.

Molday, R.S., and Moritz, O.L. (2015). Photoreceptors at a glance. J. Cell Sci. *128*, 4039–4045.

Payne, F., Lim, K., Girousse, A., Brown, R.J., Kory, N., Robbins, A., Xue, Y., Sleigh, A., Cochran, E., Adams, C., et al. (2014). Mutations disrupting the Kennedy phosphatidylcholine pathway in humans with congenital lipodystrophy and fatty liver disease. Proc. Natl. Acad. Sci. USA *111*, 8901–8906.

Robinson, J.S., Klionsky, D.J., Banta, L.M., and Emr, S.D. (1988). Protein sorting in *Saccharomyces cerevisiae*: isolation of mutants defective in the delivery and processing of multiple vacuolar hydrolases. Mol. Cell. Biol. *8*, 4936–4948.

Siniossoglou, S. (2013). Phospholipid metabolism and nuclear function: roles of the lipin family of phosphatidic acid phosphatases. Biochim. Biophys. Acta *1831*, 575–581.

Stowers, R.S., and Schwarz, T.L. (1999). A genetic method for generating *Drosophila* eyes composed exclusively of mitotic clones of a single genotype. Genetics *152*, 1631–1639.

Strakova, J., Demizieux, L., Campenot, R.B., Vance, D.E., and Vance, J.E. (2011). Involvement of CTP:phosphocholine cytidylyltransferase-beta2 in axonal phosphatidylcholine synthesis and branching of neurons. Biochim. Biophys. Acta *1811*, 617–625.

Sundler, R., Arvidson, G., and Akesson, B. (1972). Pathways for the incorporation of choline into rat liver phosphatidylcholines in vivo. Biochim. Biophys. Acta *280*, 559–568.

Taneva, S.G., Patty, P.J., Frisken, B.J., and Cornell, R.B. (2005). CTP:phosphocholine cytidylyltransferase binds anionic phospholipid vesicles in a cross-bridging mode. Biochemistry *44*, 9382–9393.

Testa, F., Filippelli, M., Brunetti-Pierri, R., Di Fruscio, G., Di Iorio, V., Pizzo, M., Torella, A., Barillari, M.R., Nigro, V., Brunetti-Pierri, N., et al. (2017). Mutations in the PCYT1A gene are responsible for isolated forms of retinal dystrophy. Eur. J. Hum. Genet. *25*, 651–655.

Thibault, G., Shui, G., Kim, W., McAlister, G.C., Ismail, N., Gygi, S.P., Wenk, M.R., and Ng, D.T. (2012). The membrane stress response buffers lethal effects of lipid disequilibrium by reprogramming the protein homeostasis network. Mol. Cell *48*, 16–27.

Vamparys, L., Gautier, R., Vanni, S., Bennett, W.F., Tieleman, D.P., Antonny, B., Etchebest, C., and Fuchs, P.F. (2013). Conical lipids in flat bilayers induce packing defects similar to that induced by positive curvature. Biophys. J. *104*, 585–593.

van der Veen, J.N., Kennelly, J.P., Wan, S., Vance, J.E., Vance, D.E., and Jacobs, R.L. (2017). The critical role of phosphatidylcholine and phosphatidylethanolamine metabolism in health and disease. Biochim. Biophys. Acta *1859*, 1558–1572.

Vanni, S., Hirose, H., Barelli, H., Antonny, B., and Gautier, R. (2014). A subnanometre view of how membrane curvature and composition modulate lipid packing and protein recruitment. Nat. Commun. *5*, 4916.

Wang, L., Magdaleno, S., Tabas, I., and Jackowski, S. (2005). Early embryonic lethality in mice with targeted deletion of the CTP:phosphocholine cytidylyltransferase alpha gene (PCYT1A). Mol. Cell. Biol. *25*, 3357–3363.

Weber, U., Eroglu, C., and Mlodzik, M. (2003). Phospholipid membrane composition affects EGF receptor and Notch signaling through effects on endocytosis during Drosophila development. Dev. Cell *5*, 559–570.

Wong, C.K. (2014). Novel mutations in PCYT1A are responsible for spondylometaphyseal dysplasia with cone-rod dystrophy. Clin. Genet. *85*, 532–533.

Yamamoto, G.L., Baratela, W.A., Almeida, T.F., Lazar, M., Afonso, C.L., Oyamada, M.K., Suzuki, L., Oliveira, L.A., Ramos, E.S., Kim, C.A., et al. (2014). Mutations in PCYT1A cause spondylometaphyseal dysplasia with cone-rod dystrophy. Am. J. Hum. Genet. *94*, 113–119.

Young, R.W. (1967). The renewal of photoreceptor cell outer segments. J. Cell Biol. *33*, 61–72.

STAR★METHODS

KEY RESOURCES TABLE

REAGENT or RESOURCE	SOURCE	IDENTIFIER
Antibodies		
Rabbit monoclonal anti-Pcyt1a	Abcam	Cat # Ab109263; RRID:AB_10859965
Goat anti-Rabbit IgG (H+L) Secondary Antibody, Alexa Fluor 594 conjugate	Thermo Fisher Scientific	Cat # A11037; RRID:AB_2534095
Goat anti-Rabbit IgG (H+L) Secondary Antibody, Alexa Fluor 647 conjugate	Thermo Fisher Scientific	Cat # A21245; RRID:AB_2535813
Mouse monoclonal anti-Rh1	DSHB	Cat # 4C5; RRID:AB_528451
Rabbit polyclonal anti-βactin	Abcam	Cat # Ab8227; RRID:AB_2305186
Rabbit anti-Calnexin	Abcam	Cat # Ab75801; RRID:AB_1310022
Anti-rabbit IgG, HRP-linked Antibody	Cell Signaling Technology	Cat# 7074; RRID:AB_2099233
Anti-mouse IgG, HRP-linked Antibody	Cell Signaling Technology	Cat# 7076; RRID:AB_330924
Chemicals, Peptides, and Recombinant Proteins		
Paraformaldehyde	Sigma Aldrich	Cat # F8775
Prolong Gold Antifade Mountant	Thermo Fisher Scientific	Cat # P36934
Prolong Gold Antifade Mountant with DAPI	Thermo Fisher Scientific	Cat #P36931
BODIPY 493/503	Thermo Fisher Scientific	Cat # D3922
DAPI	Sigma Aldrich	Cat # D9542
Monodansylpentane (MDH)	Abgent	Cat # SM1000a; MDH
Experimental Models: Cell Lines		
Mouse: OP9 cells (OP9-K clone)	José M. Ordovás laboratory, Tufts University, Boston	RRID:CVCL_KB57
Mouse: 3T3-L1	ZenBio	RRID:CVCL_0123
D. melanogaster: S2 cells	Life Technologies	Cat # R69007 RRID:CVCL_Z232
Experimental Models: Organisms/Strains		
Mus musculus: C57BL/6J (only femoral and tibial sections obtained for this study)	Laboratory of Prof. Graham R. Williams (Imperial College London, UK)	RRID:IMSR_JAX:000664
Mus musculus: *Mtp* knockout (Only liver tissue obtained for this study)	Laboratory of Prof. Mahmood Hussain (SUNY Downstate Medical Center, USA)	Khatun et al., 2012
Mus musculus: C57BL/6 (Retinal, liver and white adipose tissue used in this study)		RRID:MGI:5656552
D. melanogaster: w^{+}*; P{w*$^{+mC}$*=GAL4-ninaE.GMR*}12	Bloomington	1104; GMR-*GAL4*
D. melanogaster: w^{1118}*;;p{*GD6991}v18628/*TM6B*	VDRC	GD-CCT1 RNAi stock; VDRC ID 18628
D. melanogaster: w^{+}; P{KK108281}VIE-260B	VDRC	KK-CCT1 RNAi stock VDRC ID 100575
D. melanogaster: w^{+}; P{KK108281}VIE-260B/*CyO*; p{GD6991}v18628/*TM6B*	This study	GD and KK-CCT1 RNAi stock
D. melanogaster: w^{1118}	VDRC	GD progenitor; VDRC ID 60000
D. melanogaster: y w^{1118};P{attP, *y*$^{+}$,*w*$^{3'}$	VDRC	KK progenitor; VDRC ID 60100
D. melanogaster: w^{+}; P{KK110819}VIE-260B	VDRC	KK-CCT2 RNAi stock VDRC ID 105794
D. melanogaster: y w eyFLP;;GMR-*hid* w^{+} *FRT80B/TM6B* rec40	Ursula Weber	N/A

(*Continued on next page*)

Continued

REAGENT or RESOURCE	SOURCE	IDENTIFIER
D. melanogaster: w;;CCT1^{179} FRT80B e/TM6B e	(Gupta and Schupbach, 2003; Weber et al., 2003)	CCT1^{179}
D. melanogaster: w;;CCT^{299} FRT80B e/TM6B e	(Gupta and Schupbach, 2003; Weber et al., 2003)	CCT^{299}
D. melanogaster: eyFLP;;P^{w+} FRT80B	Ursula Weber	P^{w+} control
D. melanogaster: w; P{*Rh1-CCT1*-GFP}attP40/ *SM6a* (*CCT1* gene ID CG1049)	This study	CCT1-GFP
D. melanogaster: w; P{*Rh1-CCT2*-GFP}attP40/ *SM6a* (*CCT2* gene ID CG18330)	This study	CCT2-GFP
D. melanogaster: w; P{*Rh1-hPCYT1A*-GFP}attP40/ *SM6a*	This study	hPCYT1A-GFP
D. melanogaster: w; P{*Rh1-CCT1*-NLSm-GFP}attP40/ *SM6a*	This study	CCT1-NLSm
D. melanogaster: w; P{*Rh1-CCT1*-NLSm-GFP}attP40/ *CyO; CCT^{299} e/TM6B e*	This study	CCT1-NLSm/CCT^{299}
D. melanogaster: w; P{*Rh1-CCT1*-NLSm-GFP}attP40/ *CyO; CCT1^{179} e/TM6B e*	This study	CCT1-NLSm/CCT1^{179}
S. cerevisiae: MATα his3Δ1 leu2Δ0 lys2Δ0 ura3Δ0	Open Biosystems	BY4742
S. cerevisiae: BY4742 *pct1::NatMX6*	This study	*pct1Ä*
S. cerevisiae: BY4742 *cho2::KanMX opi3::LEU2*	Laboratory of George M. Carmen, Rutgers University, New Brunswick, New Jersey USA.	*cho2Δopi3Δ*
S. cerevisiae: BY4742 *cho2::KanMX opi3::LEU2pct1::NatMX6* YCplac33-*OPI3*	This study	*cho2Δopi3Δpct1Δ pURA-OPI3*
S. cerevisiae: BY4742 *cpt1::KanMX*	Open Biosystems	*cpt1Δ*
S. cerevisiae: BY4742 *cpt1*::KanMX *ept1::hphNT1*	This study	*cpt1Δept1Δ*
S. cerevisiae: MATα leu2-3,112 ura3-52 his3-Δ200 trp1-Δ901 suc2-Δ9 lys2-801	(Robinson et al., 1988)	SEY6210.1
S. cerevisiae: SEY6210.1 *ist2::HISMX6 scs2::TRP1 scs22::HISMX6 tcb1::KanMX6 tcb2::KanMX6 tcb3::HISMX6*	(Manford et al., 2012)	*ANDY198 (ΔTether)*
S. cerevisiae: W303 MATα tor1-1 fpr1::NAT RPL13A-2×FKBP12::TRP1	EUROSCARF	*HHY168*
S. cerevisiae: HHY168 *URA3*::YIplac211-*NUP84-mCherry KAP60-FRB::KanMX*	This study	SS1725
S. cerevisiae: SS1725 *PCT1-GFP::HISMX6*	This study	SS2473
Oligonucleotides		
Silencer™ Select Pcyt1a siRNA	Life Technologies	Cat # 4457298 Assay s64594
Silencer™ Select Negative Control No. 1 siRNA	Life Technologies	Cat # 4390843
Recombinant DNA		
eGFP-CCT1 under control of *Rh1* promoter in pCaSpeR4 plasmid	This study	pCaSpeR4-Rh1prom-*CCT1-GFP*
eGFP-CCT2 under control of *Rh1* promoter in pCaSpeR4 plasmid	This study	pCaSpeR4-Rh1prom-*CCT2-GFP*
eGFP-hPCYT1A under control of *Rh1* promoter in pCaSpeR4 plasmid	This study	pCaSpeR4-Rh1prom-*hPCYT1A-GFP*
eGFP-CCT1-NLSm (K34A,R35A,K36A) under control of *Rh1* promoter in pCaSpeR4 plasmid	This study	pCaSpeR4-Rh1prom-*CCT1*-NLSm-*GFP*
CCT1-GFP under control of *pMT* promoter in pMT/V5-HisA plasmid	This study	pMT-*CCT1-GFP*
CCT1 NLSm-GFP under control of *pMT* promoter in pMT/V5-HisA plasmid	This study	pMT-*CCT1* NLSm-*GFP*
PCT1-GFP under the control of *PCT1* promoter in *CEN/LEU2* vector	This study	YCplac111-*PCT1-GFP*
PCT1-GFP under control of *PCT1* promoter in *CEN/HIS3* vector	This study	pRS313-*PCT1-GFP*
NLS-GFP under control of *ADH1* promoter in *CEN/LEU2* vector	This study	YCplac111-*ADH-NLS-GFP*
NLS-GFP under control of *ADH1* promoter in *CEN/ADE2* vector	This study	pA5211-*ADH-NLS-GFP*

(*Continued on next page*)

Continued

REAGENT or RESOURCE	SOURCE	IDENTIFIER
PCT1-K62A/R63A/R64A/R65A-GFP under the control of *PCT1* promoter in *CEN/HIS3* vector	This study	pRS313-*PCT1*NLSm1-*GFP*
PCT1-V169M/H195A/Y200A-GFP under control of *PCT1* promoter in *CEN/HIS3* vector	This study	pRS313-*PCT1*Cmut-*GFP*
PCT1-[145-178]Δ-GFP under control of *PCT1* promoter in *CEN/HIS3* vector	This study	pRS313-*PCT1*Cdel-*GFP*
PCT1-[254-312]Δ-GFP under control of *PCT1* promoter in *CEN/HIS3* vector	This study	pRS313-*PCT1*Mdel-*GFP*
SEC63-mCherry under control of *SEC63* promoter in *CEN/URA3* vector	This study	YCplac33-*SEC63-mCherry*
PCT1-[60-66]Δ-GFP under control of *PCT1* promoter in *CEN/HIS3* vector	This study	pRS313-*PCT1*NLS1del-*GFP*
Software and Algorithms		
ImageJ/Fiji	Fiji	http://fiji.sc/
Photoshop	Adobe	http://www.adobe.com/uk/products/photoshop.html
Illustrator	Adobe	http://www.adobe.com/uk/products/illustrator.html
Prism	GraphPad	https://www.graphpad.com/scientific-software/prism/

CONTACT FOR REAGENT AND RESOURCE SHARING

Further information and requests for resources and reagents should be directed to and will be fulfilled by the Lead Contact, David B. Savage (dbs23@medschl.cam.ac.uk).

EXPERIMENTAL MODEL AND SUBJECT DETAILS

Animal Models

The mice from which specific tissue sections for immunohistochemistry were used in this study are listed in the Key Resources table. The bone and *Mtp* knockout liver tissues were obtained from C57BL/6J (male; 15 days old) and *Mtp* knockout (male; 8 weeks old) mice, respectively.

The C57BL/6 mice (male; 10 weeks old) used in this study for obtaining the wild type liver, retinal and adipose tissues were group-housed and maintained in ventilated cages with standard bedding and enrichment. Mice were maintained in a temperature and humidity-controlled room on a 12 hr light/dark cycle with *ad libitum* access to water and standard laboratory chow diet. For the fasting-refeeding paradigm, mice were fasted 16 h and refed for 6 h. All experiments were performed in accordance with the Animals (Scientific Procedures) Act 1986 and approved by the local animal ethic committees.

Eukaryotic Cell Lines and Primary Culture

The OP9-K cells (RRID: CVCL KB57) used in this study are clones derived from the parental mouse embryonic OP9 cells of unknown sex (ATCC CRL-2749). The OP9-K cells were maintained in Minimum Essential Medium α (Sigma) supplemented with 20% (v/v) fetal bovine serum (FBS), 2 mM L-glutamine, penicillin and streptomycin. They were differentiated into adipocytes in differentiation medium containing 0.2% FBS (v/v), penicillin and streptomycin using two approaches; either by addition of 900 μM oleic acid and 1 μM insulin or by addition of a hormonal mixture (1 μM dexamethasone, 0.5 mM 3-isobutyl-1- methylxanthine (IBMX) and 1 μM insulin). With the first approach, differentiation was induced when the cells reached confluence and development into mature adipocytes was achieved within 3-4 days. In the latter case, cells were grown to 2 days post-confluence and were cultured in differentiation medium supplemented with the hormonal mixture. Three days thereafter, medium was replaced with differentiation medium supplemented with insulin for a further 3 days. Development to mature adipocytes (days 8-10) was achieved by maintaining the cells in differentiation medium.

Drosophila S2 cells (ThermoFisher Scientific) used in this study were derived from a primary culture of late stage (20-24 hours old) male *Drosophila melanogaster* embryos. S2 cells were grown in Schneider's *drosophila* medium (ThermoFisher Scientific) supplemented with 10% Heat inactivated FBS, Penicillin (50 units/ml) and Streptomycin (50 ug/ml) in a CO_2 free incubator at 28°C.

Fly Stocks

For transgenic eye-specific expression in flies, the cDNA encoding CCT1, CCT2 or hPCYT1A was cloned using PCR-based cloning in BamHI and XbaI sites downstream of *Rhodopsin* (*Rh1*) promoter in a modified pCasPeR4 plasmid backbone; eGFP was also cloned N-terminal to the transgene to aid visualization of the proteins. These plasmid constructs were microinjected in embryos of w^{1118} genotype and were selected by the w^{+} marker. Fly stocks with second chromosome P-element mediated transgene insertion were identified and balanced with *SM6a*. For transgenic expression of CCT1-NLSm-GFP mutant, the predicted NLS residues ^{34}KRK^{36} were mutated to alanine residues in the WT CCT1-GFP construct by using Agilent QuickChange II XL kit before microinjection.

For eye-specific knockdown, fly stocks carrying an *upstream activation sequence* element (*UAS*)-driven siRNA transgene specific for CCT1 (GD CCT1, VDRC id 18628 and KK CCT1, VDRC id 100575) and CCT2 (KK CCT2, VDRC id 105794) were obtained from VDRC and were crossed with flies carrying eye-specific *Glass multiple reporter* (GMR) promoter driven *GAL4*. The progenitor lines of the GD/KK stocks without any siRNA transgene (KK or GD progenitor) were crossed with the GMR-*GAL4* stocks to be used as negative controls.

To obtain eye-mosaic CCT mutants, fly stocks carrying FRT sites and a single copy of the *CCT1* null alleles, CCT1^{179} (*w*;;*CCT1*^{179} *FRT80B e/TM6B e*) or CCT^{299} (*w*;;*CCT*^{299} *FRT80B e/TM6B e*) (Gupta and Schupbach, 2003; Weber et al., 2003) were crossed with stocks carrying the *FLP*/GMR-*hid* genes (*y w eyFLP*;;GMR-*hid FRT80B/TM6B* rec40). For transgenic rescue of the eye-mosaic mutants, stocks expressing the rescue transgene on chromosome 2 were first crossed with the stocks carrying the *CCT1* null allele.

All flies were reared in standard fly food medium in either bottles or vials, where fly media contained 10 g agar, 88 g dextrose, 88 g maize, 19 g yeast and 29 ml Nipagin (10% Methylparaben in absolute alcohol) per litre of sterile distilled water. Flies were maintained at 25°C and 70% humidity with alternating 12 h dark/light cycles. For the experiments, flies of either sex at 3-5 days post-eclosion were collected after being anaesthetized on CO_2 pads.

Yeast Strains, Plasmids and Culture

Yeast strains and plasmids used in this study are listed in the Key Resources table. Gene deletions and epitope tagging at the endogenous locus were achieved using a one-step polymerase chain reaction (PCR)-based method (Janke et al., 2004; Longtine et al., 1998) and confirmed by PCR. Yeast plasmids were generated using standard PCR and cloning techniques. For expression of Pct1 with a carboxyl-terminal GFP tag, Pct1 was cloned from genomic DNA in frame with GFP in both YCplac111 and pRS313 plasmids. These constructs were used as a template for site directed mutagenesis (Agilent QuickChange II XL) to generate Pct1 constructs with the mutated nuclear localization signals (NLSm1) and catalytic (C)-domain (Cmut). The NLS for Pct1 was predicted using the cNLS mapper software that was also verified by MacKinnon et al. (2009). The three residues mutated to compromise catalytic activity in the Cmut were chosen from Payne et al. (2014) and Lee et al. (2009). For the construction of Pct1 mutants lacking the membrane binding (M)-domain (pRS313-*PCT1*Mdel-GFP), the DNA fragments flanking the targeted deletion were PCR amplified and cloned into the pRS313 plasmid using the In-fusion cloning kit (Takara). For identification of the putative M-domain of Pct1, multiple protein sequence alignments were performed with homologs from different species having known M-domain boundaries (*Homo sapiens*, *Ratus norvegicus*, *Ciona intestinalis*, *Drosophila melanogaster*, *Trichoplax adherens*, *Caenorhabditis elegans*, *Hansenula polymorpha*). All plasmid constructs generated in this study were verified by DNA sequencing.

All reagents were purchased from Sigma (St. Louis, MO), unless otherwise specified. Yeast cells were grown at 30°C in a gyratory shaker (at 210 rpm) in synthetic complete (SC) medium containing 2% glucose, 0.2% yeast nitrogen base (Difco, BD, Franklin Lakes, NJ), 0.6% ammonium sulfate and amino acids drop-out (60 mg/L leucine, 55 mg/L adenine, 55 mg/L uracil, 55 mg/L tyrosine, 20 mg/L of arginine, 10 mg/L histidine, 60 mg/L isoleucine, 40 mg/L lysine, 60 mg/L phenylalanine, 50 mg/L threonine, 10 mg/L methionine, 40 mg/L tryptophan) lacking the appropriate amino acids for plasmid selection. Where indicated in the figures, 1 mM choline or 1 g/L 5-fluorotic acid (FOA) was added to the culture medium. For yeast transformation, cells were grown in YPD [2% glucose, 2% bactopeptone (BD, Franklin Lakes, NJ) and 1% yeast extract (BD, Franklin Lakes, NJ)] or selective SC medium, if plasmid selection was required, and transformed using the lithium acetate method.

For growth assays on plates, yeast cells were grown to exponential phase in selective SC medium, with the indicated supplementation where required, and 10 μL of standard 10-fold serial dilutions were spotted onto the appropriate SC plates and incubated at 30°C for 2–4 days. For time-course choline supplementation experiments, cells were grown in SC media overnight and were diluted in fresh SC media to an OD_{600}=0.5 followed by further incubation at 30°C for 24 h. The cells were then supplemented with 1mM choline and incubated further for the times indicated in figures.

METHOD DETAILS

Immunohistochemistry

Tibial and femoral growth plate sections of 15-day old wild type C57BL/6J mice and *Mtp* knockout livers of adult mice were provided by Prof. Graham R. Williams (Imperial College London, UK) and Prof. Mahmood Hussain (SUNY Downstate Medical Center, USA), respectively. Retinal, liver and white adipose tissue sections were obtained from 10 weeks old C57BL/6 wild type mice. Harvested mouse tissues were fixed with 4% paraformaldehyde and embedded with paraffin. Tissues were sectioned at 4 μm and subjected to heat-induced antigen retrieval with sodium citrate buffer at 97°C for 20 min for subsequent PCYT1A immunostaining. PCYT1A

primary antibody (ab109263, Abcam) was used at 1:10-1:100 dilution and Alexa Fluor 594 or 647-conjugated secondary antibodies (ThermoFisher) were used at 1:250-1:500 dilution. Tissues were mounted with ProLong Gold Antifade Mountant.

Immunostaining

Cells were fixed with 4% paraformaldehyde for 15 min and permeabilised with 0.5% TritonX-100 for 10 min prior to blocking with 3% BSA for 1 h at room temperature (RT). Immunostaining with PCYT1A primary antibody (ab109263, Abcam) at 1:100 dilution was performed at 4°C overnight and was followed by washing with PBS and incubation with Alexa Fluor 594 or 647-conjugated secondary antibodies at 1:1000 dilution for 1 hour at RT. After washing with PBS, lipid droplets were stained with BODIPY 493/503 at 1:1000 dilution for 30 min at RT. Cells were mounted on microscope slides with ProLong Gold Antifade Mountant with DAPI.

Confocal Microscopy of Dissociated Ommatidia and S2 Cells

Fluorescence microscopy was carried out on freshly dissociated ommatidia from adult or pupal stage flies as indicated. Cold anesthetized flies were decapitated, and retinal tissue was dissected out in cold ringer solution (195 mM NaCl, 5 mM KCl, 1.8 mM $CaC1_2$, 4 mM $MgCl_2$ and 10 mM TES). The ommatidia were mechanically dissociated in ringer solution supplemented with 10% FBS using the method described in Hardie (1991). The ommatidia were transferred to a Petri dish with a glass coverslip bottom and stained with DAPI (final conc. 0.2 μg/ml) and incubated for 10 min at RT before imaging.

Drosophila S2 cells were transfected with expression plasmids having an inducible metallothionein promoter (*pMT*) by using lipofectamine LTX reagent following the manufacturer's protocol. Expression was induced by addition of 500 μM $CuSO_4$ to the medium 24 h after transfection. For the oleic acid loading experiment, 6 h after the induction of expression, oleic acid (900 μM) was added to the medium and the cells were incubated further for 24 h. BODIPY 588/568 C12 was added along with the oleic acid (1:1000 dilution) to allow lipid droplet visualization at the end. Prior to imaging, cells were washed with PBS and stained with DAPI for 10 min.

All images were obtained from live cells using the 63X immersion oil objective (1.3 NA) on the Leica TCS SP8 confocal microscope. The following excitation/emission settings were used: GFP (488 nm/ 494 – 562nm), DAPI (405nm/ 420-480nm); BODIPY 558/568 C12 (520 nm/ 589nm - 671nm).

Where necessary the signal level or brightness in images were adjusted using Adobe Photoshop to aid visualization.

Electroretinograms (ERGs)

ERGs were recorded as described previously (Hardie et al., 2015). Live flies were placed in trimmed pipette tips with their heads protruding and immobilised with low-melting point wax. A low-resistance (~10 MΩ) glass microelectrode filled with fly Ringer (140 mM NaCl, 5 mM KCl, 1.5 mM $CaCl_2$, 4 mM $MgCl_2$) was inserted into the eye for recordings and a similar reference electrode was inserted into the head. Signals were amplified by a DAM60 preamplifier (World Precision Instruments) and sampled and analysed using pClamp software (Molecular Devices CA). Response intensity functions were determined from the negative going maintained component of responses to 1 sec flashes from an ultrabright red (640nm) LED fixed at a distance of ~5mm from the eye.

Immunoblotting

For preparation of lysate from fly heads, 1x NuPAGE LDS sample buffer (Life Technologies) supplemented with 100mM DTT was added to the tissues (5μl per fly head) in 1.5ml micro-centrifuge tubes and tissues were lysed by multiple rounds of homogenization with a pestle homogenizer and boiling at 90°C. The lysate was separated from tissue/cell debris by centrifugation at 12,000 rpm for 20 min and resolved by SDS-PAGE.

Human fibroblast and 3T3-L1 cells were lysed in RIPA buffer (Sigma) supplemented with Complete-Mini Protease Inhibitor (Roche) and PhosSTOP Phosphatase Inhibitor (Roche). 25–40 μg of protein lysate, quantified using Bio-Rad DC protein quantification kit, was diluted in NuPAGE 4× LDS sample buffer (Life Technologies) containing 10% β-mercaptoethanol and subjected to SDS-PAGE.

Following transfer onto nitrocellulose membranes using the iBLOT dry blotting system (Life Technologies), membranes were washed in Tris-buffered saline containing 0.1% (v/v) Tween 20 (TBST; Sigma), and then blocked for 1 h at room temperature in 5% (w/v) powdered skimmed milk diluted in TBS-T. Membranes were further incubated with appropriate primary antibodies for 16 h at 4°C. Primary antibodies were diluted as follows: mouse anti-Rh1, 1:1000 in 3% (w/v) BSA-TBS-T; rabbit anti-β actin, 1:1000 in 3% (w/v) BSA-TBS-T; anti-PCYT1A, 1:1000 in 5% (w/v) milk–TBS-T and anti-calnexin, 1:1000 in 5% (w/v) milk–TBS-T. All blots were washed with TBS-T and incubated for 1 h at room temperature with the specific secondary HRP-conjugated antibody (1:5000 in 5% (w/v) milk-TBS-T) listed in the Key Resources table. The blots were washed again and developed using the Immobilon Western Chemiluminescent HRP Substrate (Millipore). All images were acquired on the ChemiDoc XRS imaging system (BioRad).

Electron Micrographs

Cold anesthetized flies were decapitated and the heads were fixed in cold primary fixative (2 % glutaraldehyde, 2 % paraformaldehyde, 2 mM calcium chloride in 0.05 M sodium cacodylate buffered to pH 7.3) on a transparent silicone dissection dish using 0.1mm insect pins with their proboscis facing upwards and a sharp razor blade was used to remove the proboscis and bisect the heads which were incubated in the primary fixative overnight at 4°C. The half-heads were then washed (5 times for 3 min each) with cold 0.05 M sodium cacodylate buffer (pH 7.4) containing 2 mM calcium chloride and incubated in the same buffer supplemented with 1% osmium tetroxide and 1.5% potassium ferricyanide for 18-48 h at 4°C. After a rinse (5 times with distilled water), the half-heads were incubated in 1% thiocarbohydrazide for 30 min at RT. After another rinse, incubated in 2% aqueous osmium

tetroxide for 2 h at RT; and rinsed again before incubation in 2% uranyl acetate in 0.05 M maleate buffer (pH 5.5) overnight at 4°C. The tissue was dehydrated by double washes each of 50%, 70%, 90% and 100% ethanol, followed by two washes with acetone and acetonitrile, with drying in between each solvent change. Post-dehydration, the half-heads were infiltrated in two successive overnight incubations with 1:1 and 1:2 acetonitrile: Quetol resin mixtures (without BDMA) with gentle agitation at RT. Lastly, the samples were transferred to Quetol resin with BDMA and incubated at RT for five days. The half-heads were then transferred to moulds with fresh resin and oriented in desired angles before curing at 65°C for two days. The embedded half-heads were first sectioned (0.5 μm thickness) using a glass knife mounted in an ultramicrotome (Reichert-Jung Ultracut E, Germany) and were stained on glass slides with Toluidine Blue for observation under a light microscope. Once the desired orientation and depth was observed, ultra-thin sections (80 nm thickness) were cut using a diamond cutting knife (DiATOME Ultra 45°, USA) and sections were retrieved on EM grids for imaging using the Technai G2 80-200 kV transmission electron microscope.

Yeast Confocal Microscopy, Image Analysis and Quantifications

Yeast cells were grown to the indicated growth phase at 30°C in selective SC medium, pelleted and mounted on slides (ThermoFisher Scientific). Cells were immediately imaged at RT using a Leica TCS SP8 confocal microscope with 63x 1.4 numerical aperture oil immersion objective lens. In a sequential scan using white light laser, GFP was excited at 488 nm and the emission signal was collected between 494 and 562 nm; mCherry was excited at 587 nm and the emission was collected between 594 and 719 nm. For lipid droplet labelling, yeast cells were stained for 10 min with 10 μM monodansylpentane (MDH; SM1000a; Abgent). MDH signal was acquired using diode laser at 405 nm excitation and emission was collected between 420 and 480 nm. Images were analysed using Leica LAS AF Lite software and ImageJ. Quantification of lipid droplet area was performed using ImageJ. Lipid droplet structures which were larger than 20 pixels and smaller than 3 pixels were filtered by FFT Bandpass Filter, with threshold adjustment used to assist the calculation of the particles.

Quantification of Pct1-GFP localization was performed using ImageJ by drawing a line of 5-pixel average width across the nucleus. Plotted pixel intensities were used to determine Pct1-GFP localisation. Pct1-GFP was scored as intranuclear when the Pct1-GFP pixel intensity increased between the maximum pixel intensity of Sec63-mCherry (an ER marker surrounding nucleus) and scored as nuclear membrane bound when the maximum pixel intensity for both GFP and mCherry overlapped. To detect Pct1-GFP association with LDs, 3D images were acquired and analysed in IMARIS software to determine their co-localization.

FLIP Assay

Photobleaching experiments were performed on a Leica TCS SP8 confocal microscope with the optional FRAP Booster enabled. Yeast cells expressing Pct1-GFP and Sec63-mCherry were pelleted and imaged at room temperature. Images were acquired using a 63x oil immersion objective lens. After acquiring two images at 5-sec intervals, selected regions of interest were photobleached with 3 iterations of 100% laser power (white light laser) at 488 nm. The fluorescence intensity of GFP at the regions of interest was recorded for another 8 frames at 15 sec intervals. Photobleaching cycle was repeated for another 4 times. For data analyses, the fluorescence intensity of GFP was corrected by the loss of GFP fluorescence intensity obtained from cells under identical conditions but without a photobleaching event. The distance of the cells was greater than 3.5 μm.

Yeast Lipid Profiling

Yeast cells were grown to the indicated growth phases and prepared for mass spectrometry analysis as previously described (Folch et al., 1957) with minor modifications. Whole yeast cells (50 mg) were homogenized in 1 mL 2:1 chloroform-methanol mixture (v/v) and 100 mg 0.5 mm diameter glass beads (BioSpec products) in a FastPrep-24 instrument (MP biomedicals), using five short pulses of 5 m/s for 1 min and 1 min on ice between each pulse. The homogenized cells were emulsified by addition of 400 μL sterile water and thorough mixing for 1 min using Vortex-Genie 2 (Scientific Industries). The samples were centrifuged at 13000 rpm for 10 min. The organic layer was collected and the remaining mixture was used in a second lipid extraction by following the same procedures. The organic layers from both extractions were combined in an amber 2 mL glass container (Agilent Technologies) and allowed to air-dry in a fume hood overnight.

For the lipid analysis, an LCMS method similar to that described previously was used (Lu et al., 2016; Koulman et al., 2009). After addition of 60 μl internal standard in methanol (containing: Cholesteryl-2,2,3,4,4,6-d6-octadecanoate CE(18:0-d6), Myristoylphosphocholine-d42 LPC(C14:0)-d42, 1-palmitoyl(d31)-2-oleoyl-glycero-3-phosphocholine PC(C16:0-d31/C18:1), 1-palmitoyl(d31)-2-oleoyl-glycero-3-phosphoethanolamine PE(C16:0-d31/C18:1), N-palmitoyl(d31)-d-erythro-sphingosylphosphorylcholine (16:0-d31 SM) M(C16:0-d31), Glyceryl-tri(hexadecanoate-d31) (48:0-d93 TAG) al at 10μg/ml), samples were vortexed and 740 μl of 4:1 mix of 2-propanol and acetonitrile was added.

Using an UltiMate 3000 Rapid Separation LC System (Thermo Fisher Scientific, Hemel Hempstead, United Kingdom) full chromatographic separation of intact lipids was achieved: briefly, injection of 5 μL (partial loop) onto an Acquity UPLC® BEH, stationary phase C18, 130 Å, 1.7 μm, I.D. 2.1 mm X 50 mm reversed phase UPLC-column maintained at 55°C. Mobile phase A was made up of 6:4, acetonitrile and water with 10 mM ammonium formate solution. Mobile phase B was made up of 9:1, 2-propanol and acetonitrile with 10 mM ammonium formate solution. The flow was maintained at 1000 μL / min through the following gradient changes. Time (Minutes) % Mobile phase A: 0.00 min / 60%A; 0.40 min / 57% A; 0.45 min / 50% A; 2.40 min / 46% A; 2.45 min / 30% A; 2.90 min / 19% A; 4.00 min / 1% A; 5 min / 1% A; 7 min /60% A; 8.00 min / 60% A.

Total run time per sample was 8 min with a cycle time of 10 min during which the column was re-equilibrated and the injection needle was washed (washing solution is 9:1, IPA and ACN with 0.2 % formic acid). The mass spectrometer used was the Thermo Scientific Exactive Orbitrap (Thermo Fisher Scientific, Hemel Hempstead, United Kingdom) with a heated electrospray ionisation source (HESI). The mass spectrometer was tuned routinely using different positive ionisation calibration solution (recommended by Thermo Scientific). Additionally, the HESI was optimised at 50:50 mobile phase A to mobile phase B for spray stability (Capillary temperature; 380°C, source heater temperature; 420°C, sheath gas flow; 60 (arbitrary), auxiliary gas flow; 20 (arbitrary), sweep gas; 5 (arbitrary), source voltage; 3.5kV. The mass spectrometer resolution was set to 50,000 with a full-scan range of m/z 110 to 1200 Da cycling between positive and negative mode.

For each lipid the area under the curve was determined using high resolution extracted ion chromatograms with a window of 5 ppm and expressed relative to the internal standard for that lipid class.

Estimates of the Lipid Contribution to Membrane Stored Curvature Elastic (SCE) Stress

To a first order approximation, the two material parameters C_0 (the spontaneous curvature) and K_M (the bending modulus) in combination govern the membrane SCE stress (τ) within a lipid bilayer, Equation 1.

$$\tau = -2K_M C_0 \quad \text{(Equation 1)}$$

Estimates of curvature elastic τ can be made using a linear mixing rule, where C_0 for a mixture of lipids (C_{0mix}) is determined from C_0 values for the individual lipid components of the mixture, using a principle of ideal additive mixing (Dymond, 2016). Thus, C_{0mix} for a mixture of two lipids (A, B) in a bilayer is given by Equation 2, x_B is the mole fraction of lipid B and C_{0A} and C_{0B} are the spontaneous curvatures of lipids A and B respectively.

$$C_{0mix} = (1 - x_B)C_{0A} + x_B C_{0B} \quad \text{(Equation 2)}$$

K_M varies by a relatively small amount in comparison to C_0 for lipids and an average value (5.0×10^{-20} J) was used for estimatingτ.

Lipid spontaneous curvatures are defined as the $C_0 = 1/R_0$ where R_0 is the radius of curvature of an unstressed monolayer cylinder of lipids in an inverse hexagonal lyotropic liquid crystal phase, when measured at the pivotal plane. The pivotal plane is that point along the length of the lipid molecule where the area occupied does not change on bending the lipid monolayer.

Increases in lipid unsaturation, chain branching and to a lesser extent chain length make R_0 tighter and thus increase SCE stress in membranes. Changes to lipid headgroups are less straight forward to predict, for neutral headgroups the size of the headgroup is the dominating feature, assuming hydrocarbon chain structure is unchanged. Thus, smaller head groups make R_0 tighter and increase SCE stress. Charged headgroups introduce the complication of greater headgroup-headgroup repulsion, which favours less tight values of R_0 and can decrease SCE stress.

Estimates of the SCE stress for membranes composed of the lipidomes reported were made, using Equations 1 and 2 above. When C_0 values of lipids with the correct chain length were not available in the literature, C_0 for a lipid with the same headgroup and with the same distribution of unsaturation in each chain, with overall similar chain length was used. Estimates of lipid contribution to stored curvature elastic stress were calculated by including lipid compositions for PC, PE, PS, PA and PG lipids. PI lipids were omitted due to their being no C_0 values in the literature. A sensitivity analysis showed the inclusion of PI lipids, with speculative C_0 values, had no significant effect on the trends reported, due to their relatively low abundance in the membrane. DAG and TAG lipids were omitted from this analysis since these are predominantly associated with lipid droplets (Kuerschner et al., 2008). This analysis was also repeated with the DAG levels included, with very similar patterns of the results, likely reflecting that DAG levels did not change significantly over time (Figure 7).

Estimate of the Total Membrane Stored Curvature Elastic (SCE) Stress of Cells (Referred to Herein as P_{SCE})

P_{SCE} is a data-driven metric of the total SCE stress in a cellular system i.e. a proxy of protein and lipid contributions to SCE stress. It is determined from the absolute amounts of lipid using previously published methodology (Dymond, 2016), using the results of a data-driven modelling approach. Where P_{SCE} is defined by a ratio control function, Equation 3, which calculates the balance of lipids that increase stored curvature elastic stress (type II lipids) and lipids that decrease SCE stress (type 0 lipids). P_{SCE} is calculated from the lipidome for each population of cells,

$$P_{SCE} = \frac{\sum_{n=0}^{b} w_n \, [L_{II,n}]}{\sum_{m=0}^{a} \frac{1}{wm} \, [L_{0,m}]} \quad \text{(Equation 3)}$$

Where $[L_{II}, {}_n]$ denotes the concentration of type II lipid n and $(w)_n$ is a weighting factor for lipid n. Similarly $[L_0, {}_m]$ is the concentration of type 0 lipid m and $(w)_m$ is the weighting factor for lipid m. The variables a and b are the total numbers of type 0 and type II lipids respectively.

Values of the weighting factor w were obtained from a data driven modelling approach and taken from previously published studies (Dymond, 2016), where cells were cultured under conditions that altered their membrane composition. w was determined from a coarse-grained model of lipid structure based on well-established trends, of how the number of unsaturations in each lipid chain and the headgroup structure contribute to SCE stress. The final w values selected gave the lowest variance in P_{SCE} across a range of asynchronous cell populations and thus reflect, in a coarse-grained way, both protein and lipid contributions to total membrane

SCE stress. The two sets of lipids (type II and type 0) are determined from their w values compared to w for the pivot lipid (w_{Lp}). Lipids with $w > w_{Lp}$ are denoted type II, lipids with $w \leq w_{Lp}$ are denoted type 0. Previous work shows that this pivot lipid species is PE (34:1).

Calculations of P_{SCE} were performed using PC, PE, PS, PA, PI and DAG lipids. TAG lipids were omitted due their location in lipid droplets rather than the within cellular membranes. PG was omitted from the analyses, due to their being currently no parameters available to incorporate it. A sensitivity analysis showed that the low abundancy of PG lipids means that their inclusion in the models has no effect on the data and trends reported.

QUANTIFICATION AND STATISTICAL ANALYSIS

Quantitative data are reported as mean ± SD. As indicated in the figure legends, differences between means were assessed by two-tailed Student's t tests or one-way ANOVA with Bonferroni multiple comparisons test using GraphPad Prism software (GraphPad, San Diego). Statistical significance was defined as $p < 0.05$.

Developmental Cell, Volume *45*

Supplemental Information

PCYT1A Regulates Phosphatidylcholine Homeostasis from the Inner Nuclear Membrane in Response to Membrane Stored Curvature Elastic Stress

Afreen Haider, Yu-Chen Wei, Koini Lim, Antonio D. Barbosa, Che-Hsiung Liu, Ursula Weber, Marek Mlodzik, Kadri Oras, Simon Collier, M. Mahmood Hussain, Liang Dong, Satish Patel, Anna Alvarez-Guaita, Vladimir Saudek, Benjamin J. Jenkins, Albert Koulman, Marcus K. Dymond, Roger C. Hardie, Symeon Siniossoglou, and David B. Savage

SUPPLEMENTAL INFORMATION

Fig. S1

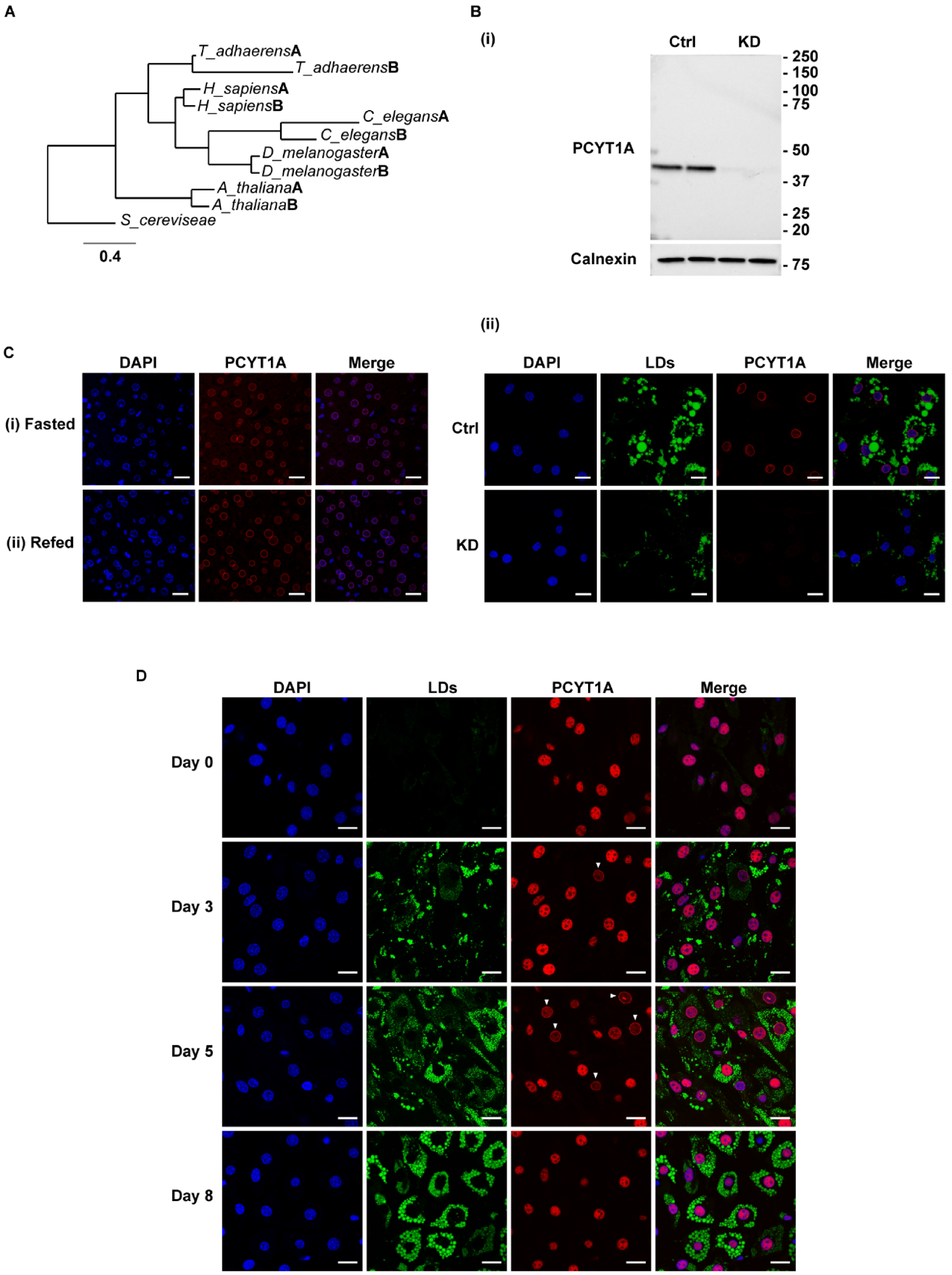

Figure S1. Related to figure 1. PCYT1A nuclear localization in WT mouse hepatocytes and differentiating OP9 adipocytes.

(A) CCT phylogenetic tree based on the alignment of sequences comprising the catalytic and M-domain of CCT from selected Metazoan, fungal and plant representatives. UniProt sequence IDs from top to bottom: B3RI62, B3RI63, P49585, Q9Y5K3, Q8IU09, P49583, Q7K4C7, Q9W0D9, Q9ZV56, F4JJE0, P13259; Labels A and B distinguish the paralogues in one species. Alignments were carried out with PROMALS (http://prodata.swmed.edu/promals/promals.php) and the tree was calculated with PhyMl algorithm with aLTR test as implemented in Phylogeny.fr package (http://www.phylogeny.fr). The branch length is proportional to the number of substitutions per site.

(B)Verification of the specificity of a PCYT1A primary antibody.

(i) PCYT1A immunoblotting using lysates obtained from mouse OP9 pre-adipocytes transfected with 30 nM non-targeting negative control (Ctrl) or *PCYT1A* specific siRNA (KD); Calnexin is used as a loading control. (ii) Immunostaining of PCYT1A in mouse OP9 adipocytes at day 2 of differentiation (induction with insulin & oleic acid as described in Methods). PCYT1A shows nuclear localization in cells treated with negative control siRNA (Ctrl), while it is almost undetectable in cells treated with *PCYT1A* specific siRNA (KD). BODIPY (green) was used to stain lipid droplets (LDs) as described in Methods.

(C) PCYT1A localizes to the nuclear membrane of hepatocytes from 10-week old WT mice (i) fasted for 16 h or (ii) fasted for 16 h and refed for 4 h.

(D) During adipogenic differentiation induced by hormonal mixture (dexamethasone, IBMX and insulin) in OP9 cells, PCYT1A is found in the intranuclear region in pre-adipocytes and transiently translocates to nuclear membrane (cells marked with white arrow heads) between days 3 and 5 of differentiation. BODIPY (green) was used to stain lipid droplets (LDs) as described in Methods.

Scale bars, 20 μm.

Fig. S2

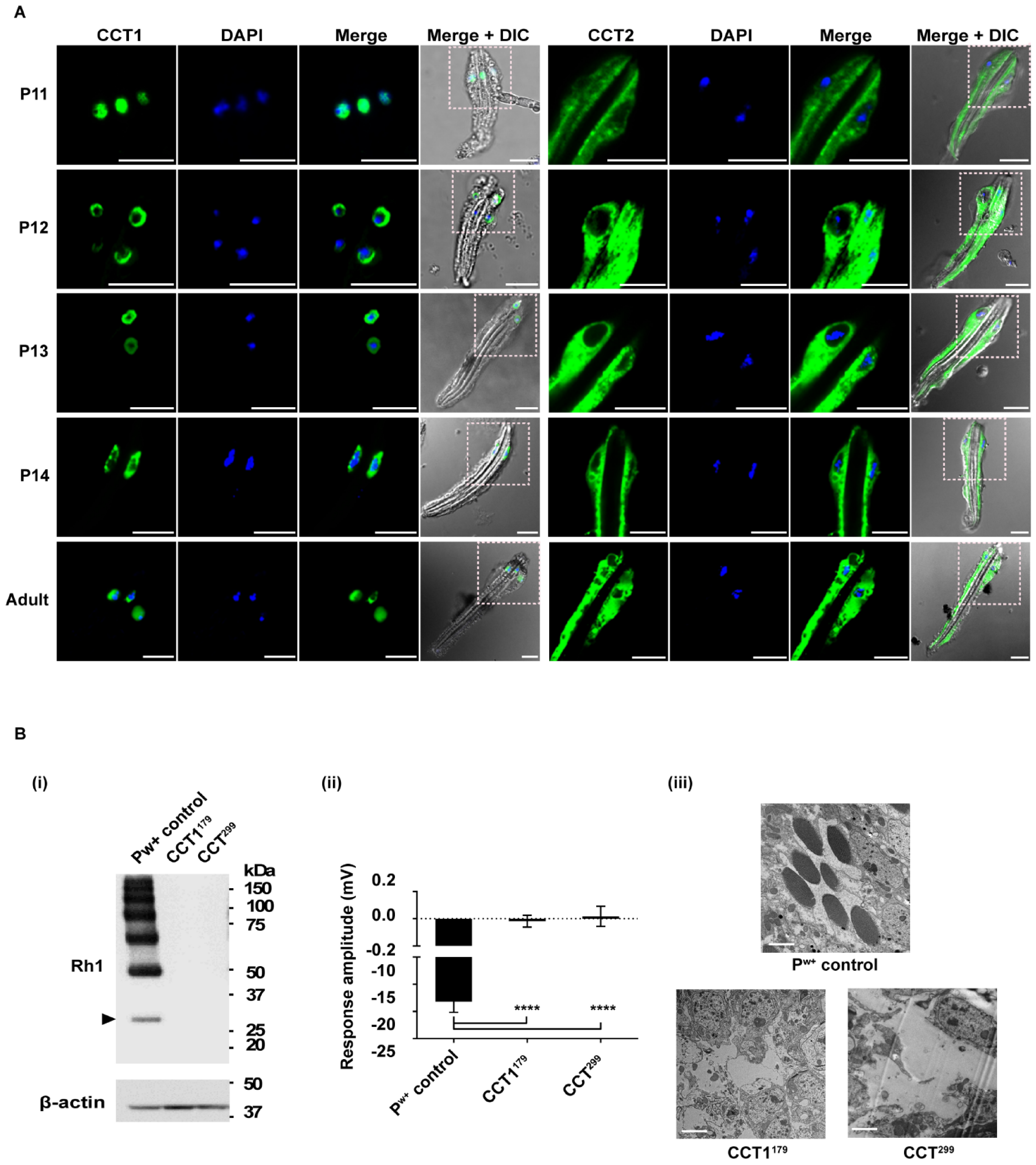

A
CCT1
DAPI
Merge
Merge + DIC
CCT2
DAPI
Merge
Merge + DIC
P11
P12
P13
P14
Adult
B
(i)
(ii)
(iii)
Pw+ control
CCT1179
CCT299
kDa
150
100
75
50
37
25
20
Rh1
β-actin
50
37
Response amplitude (mV)
0.2
0.0
-0.2
-10
-15
-20
-25
****
****
Pw+ control
CCT1179
CCT299
Pw+ control
CCT1179
CCT299

Figure S2. Related to figure 2. CCT1 is associated with the nuclear membrane in photoreceptors during pupal development.

(A) Individual ommatidia from different pupal (P11-14) and adult stages of transgenic flies expressing N-terminal GFP-tagged CCT1 or CCT2 were dissociated, stained with DAPI and imaged as described in methods. Merged fluorescent channels are shown with the DIC images (merge + DIC) for context. All panels are zoomed in regions from the boxed region in the 'merge + DIC' panel. Scale bars, 10 µm.

(B) Eye-specific GMR-hid *CCT* null flies, CCT^{179} (*CCT1*Δ) and CCT^{299} (*CCT1*Δ and *CCT2*Δ) are blind and have defective rhabdomere development (i) Rhodopsin expression is essentially undetectable in flies with *CCT* null eyes compared to the wild-type control (P^{w+}) as seen in immunoblots probed with an Rh1-antibody. (ii) Response amplitude generated in ERG recordings at maximal intensity of light was negligible in *CCT* null eyes. Data are mean ± SD from 4-5 flies of each genotype. One-way ANOVA with Bonferroni multiple comparisons ****, $p<0.0001$. (iii)Transmission electron micrographs of retinal sections from the wild-type flies (P^{w+} control) and flies with *CCT* null eyes ($CCT1^{179}$ and CCT^{299}). Scale bars, 2µm.

Fig. S3

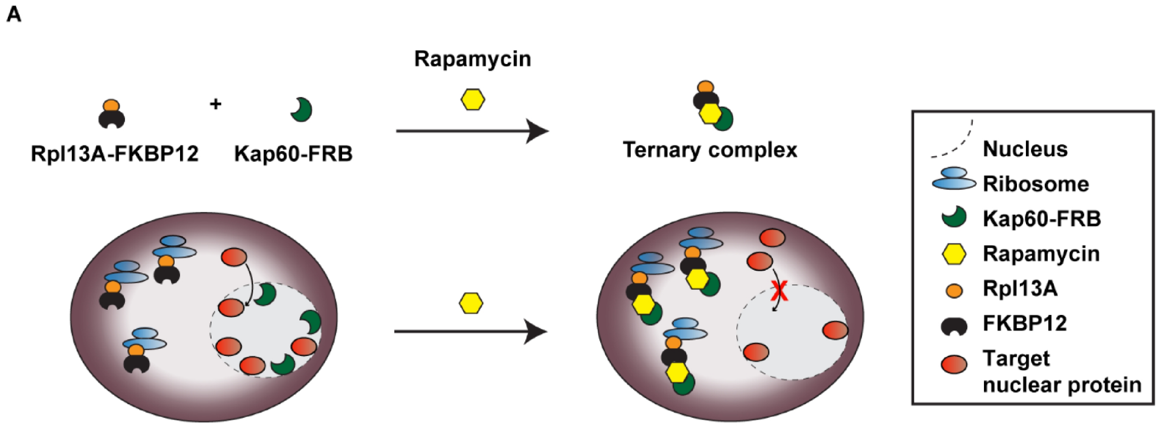
A
Rapamycin
Rpl13A-FKBP12
+
Kap60-FRB
Ternary complex
Nucleus
Ribosome
Kap60-FRB
Rapamycin
Rpl13A
FKBP12
Target
nuclear protein

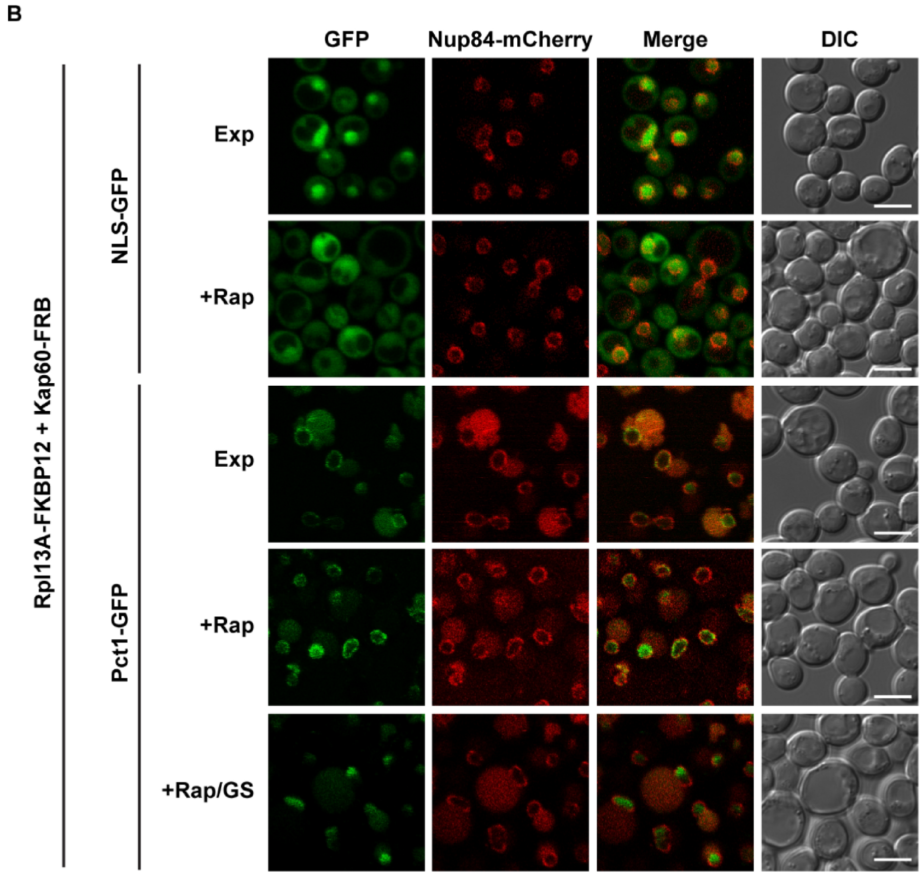
B
GFP
Nup84-mCherry
Merge
DIC
Rpl13A-FKBP12 + Kap60-FRB
NLS-GFP
Pct1-GFP
Exp
+Rap
Exp
+Rap
+Rap/GS

Figure S3. Related to figure 3. Pct1 associates with the inner nuclear membrane.

(A) Schematic of the anchor-away technique (adapted from Haruki *et al*., 2008). Rapamycin promotes tethering of the karyopherin Kap60 (Kap60-FRB) to the ribosomal protein Rpl13A (Rpl13A-FKBP12), due to heterodimerization of FKBP12 and FRB, causing depletion of Kap60 from the nucleus and would block the nuclear import of target proteins.

(B) Anchor-away strain expressing Rpl13A-2xFKBP12, Kap60-FRB, Nup84-mCherry (nuclear membrane marker) and Pct1-GFP was grown to the exponential phase (Exp) and treated with rapamycin (+Rap) for 15 min. Glucose starvation (+Rap/GS) was induced after the initial rapamycin treatment by transferring the cells into rapamycin-medium lacking glucose for 30 min. To confirm that depletion of Kap60 prevents nuclear import, GFP fused to a known nuclear localisation signal (NLS-GFP) was used as a positive control. Cells were imaged as described in Methods. Scale bars, 5 µm. NLS-GFP concentrates in the nucleus in untreated cells but it is increasingly redistributed to the cytosol following Kap60 inactivation demonstrating that nuclear import is successfully blocked.

Fig. S4

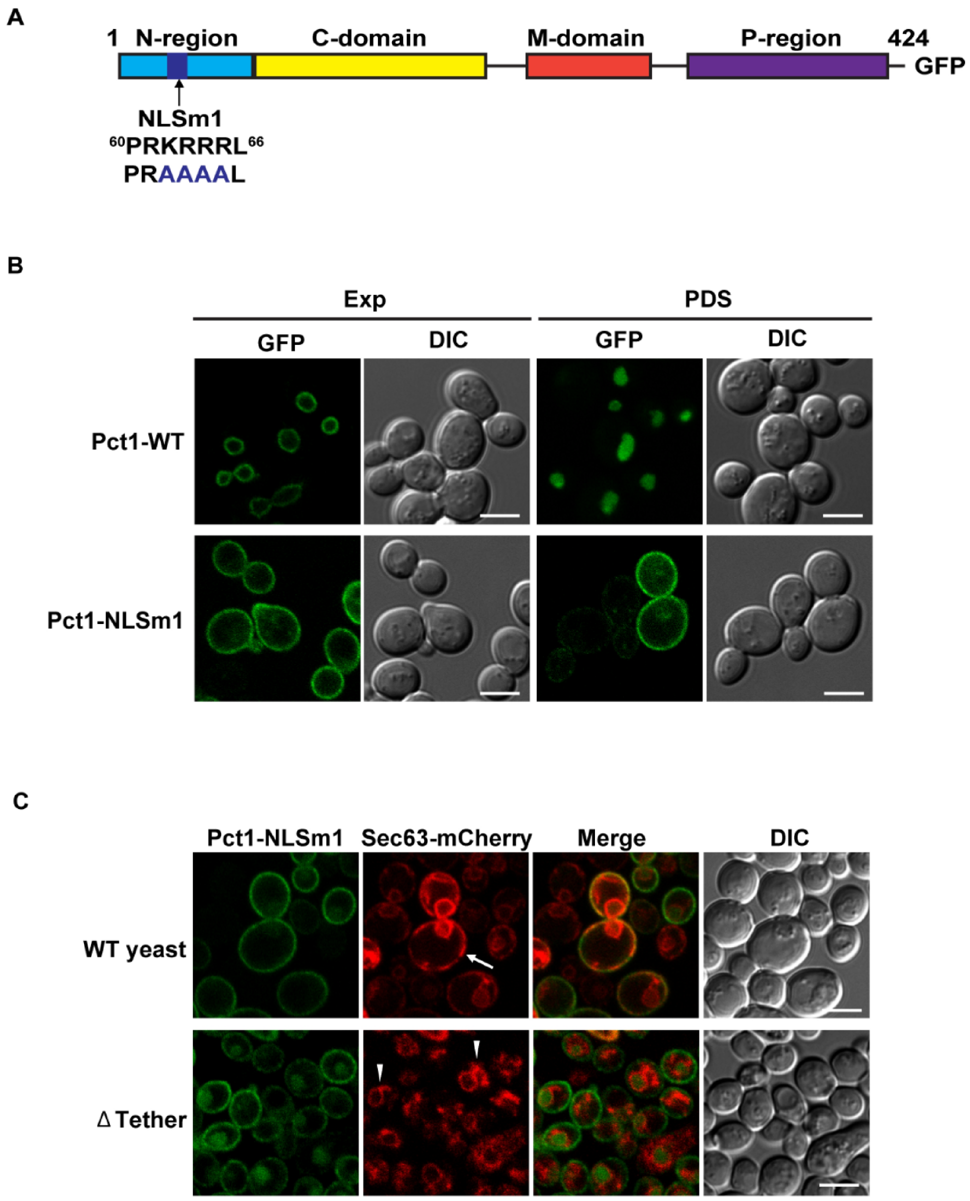
A
1 N-region
C-domain
M-domain
P-region
424
GFP
NLSm1
60PRKRRRL66
PRAAAAL
B
Exp
PDS
GFP
DIC
GFP
DIC
Pct1-WT
Pct1-NLSm1
C
Pct1-NLSm1
Sec63-mCherry
Merge
DIC
WT yeast
Δ Tether

Figure S4. Related to figure 3. Abrogation of the Pct1 nuclear localisation signal (NLS1) sequence inhibits Pct1 nuclear translocation.

(A) Diagram showing the putative nuclear localization sequence (NLS) of Pct1. Basic residues of the NLS motif were mutated to alanine (blue) in NLSm1-Pct1-GFP. C-domain, catalytic domain; M-domain, membrane binding domain; P-region, phosphorylated region.

(B) *pct1*Δ cells expressing the GFP-tagged wild-type Pct1 (Pct1-WT) or NLSm1 mutant (Pct1-NLSm1) were grown to the indicated growth phase and imaged as described in Methods. Scale bars, 5 µm.

(C) GFP-tagged Pct1-NLSm1 and Sec63-mCherry (ER marker) were expressed in the Δtether strain and the corresponding wild type (WT) yeast strain, grown to exponential phase and imaged as described in Methods. White arrows mark the normal cortical ER in WT strain and arrowheads indicate the collapsed cortical ER in the Δtether cells. Scale bars, 5 µm.

Fig. S5

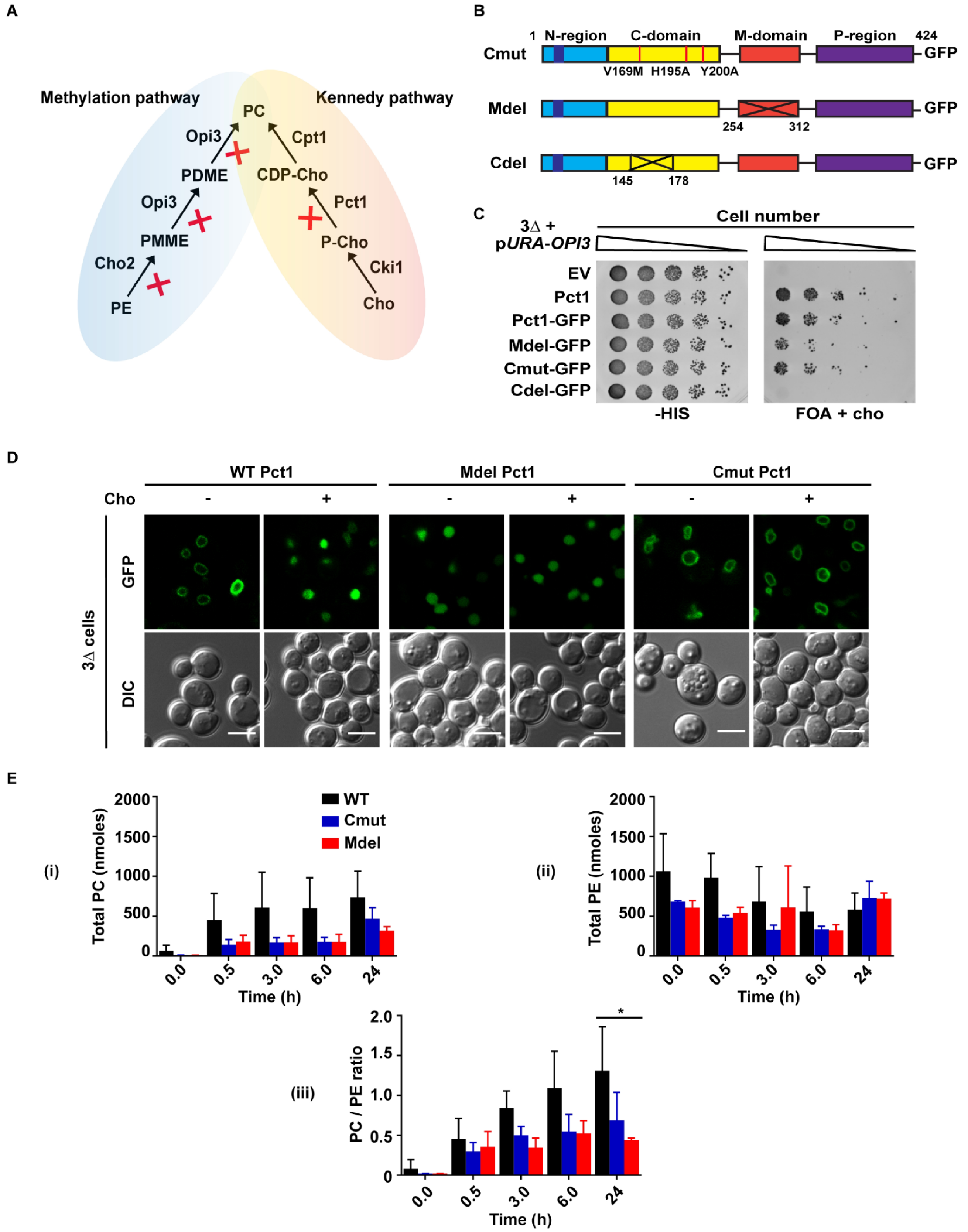
A
Methylation pathway
Kennedy pathway
PC
Opi3
Cpt1
PDME
CDP-Cho
Opi3
Pct1
PMME
P-Cho
Cho2
Cki1
PE
Cho
B
1 N-region C-domain M-domain P-region 424
Cmut
GFP
V169M H195A Y200A
Mdel
254 312
Cdel
145 178
C
3Δ +
pURA-OPI3
Cell number
EV
Pct1
Pct1-GFP
Mdel-GFP
Cmut-GFP
Cdel-GFP
-HIS
FOA + cho
D
WT Pct1
Mdel Pct1
Cmut Pct1
Cho
3Δ cells
GFP
DIC
E
(i)
Total PC (nmoles)
WT
Cmut
Mdel
Time (h)
(ii)
Total PE (nmoles)
(iii)
PC / PE ratio
*

Figure S5. Related to figure 5. Validation of the 3Δ (*cho2Δ opi3Δ pct1Δ*) *Saccharomyces cerevisiae* model.

(A) Schematic of the enzymes involved in PC biosynthesis. Deletion of *CHO2* and *OPI3* blocks the methylation pathway while deletion of Pct1 blocks the CDP-choline branch of the Kennedy pathway. PC, phosphatidylcholine; PE, phosphatidylethanolamine; P-cho, Phosphocholine; CDP-Cho, CDP-choline, PMME, phosphatidyl-N-mono-methylethanolamine; PDME, phosphatidyl-N, N dimethylethanolamine.

(B) Schematic illustration of the Pct1 domain organisation indicating the mutations in the Cmut, Mdel and Cdel Pct1 mutants. C-domain, Catalytic domain; M-domain, Membrane binding domain. Substitution mutations are indicated with red bars and targeted deletions are indicated with crossed boxes.

(C) 3Δ cells carrying Ycplac33-*URA3-OPI3* were transformed with a *CEN/HIS3* plasmid expressing either wild-type Pct1, Pct1-GFP or mutant- Mdel-GFP, Cmut-GFP and Cdel-GFP. Empty *CEN/HIS3* plasmid (EV) was used as a negative control. Cells were grown to exponential phase and serial dilutions of liquid cultures were spotted onto SC plates lacking histidine (-HIS) or supplemented with FOA.

(D) 3Δ cells expressing GFP-tagged WT, Mdel or Cmut Pct1 were grown to exponential growth phase with (+) or without (-) choline and imaged as described in Methods. DIC images are shown for context. Scale bars, 5 µm.

(E) 3Δ cells expressing GFP-tagged WT, Mdel or Cmut Pct1 were grown for 24 hours (h) followed by addition of 1 mM choline. Cells were collected before (0.0 h) and after choline supplementation at the indicated time points for lipidomic analysis. (i) PC and (ii) PE levels at the indicated time points are shown in nmoles (per 50 mg yeast) relative to internal standards. (iii) PC/PE ratio at the indicated time points. Data are shown as mean ± SD from independent repeats (n=8 for WT; n=3 each for Cmut and Mdel Pct1). *P <0.05 at 24 h using one-way ANOVA.

Fig. S6

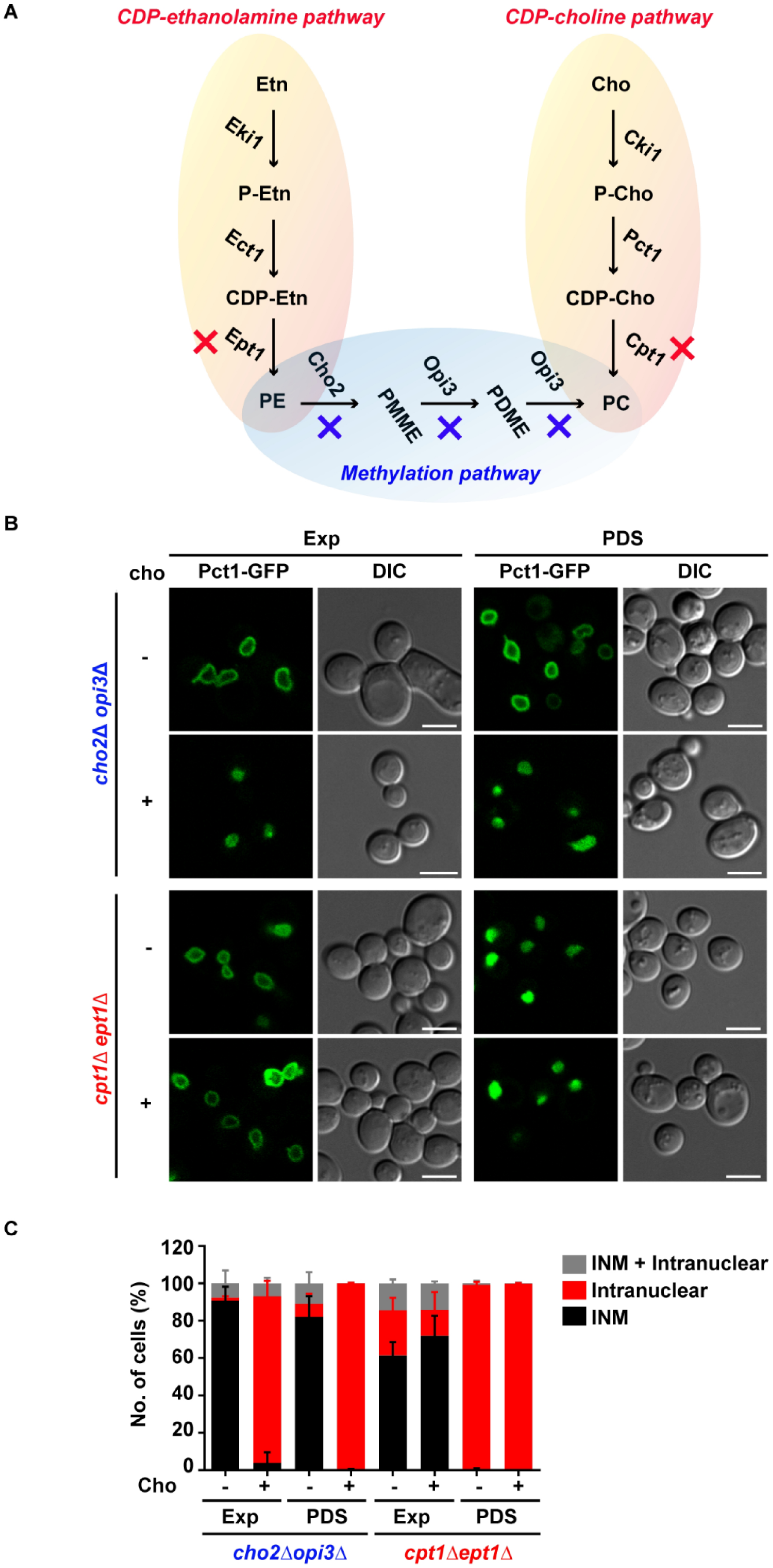
A
CDP-ethanolamine pathway
CDP-choline pathway
Etn
Eki1
P-Etn
Ect1
CDP-Etn
Ept1
PE
Cho
Cki1
P-Cho
Pct1
CDP-Cho
Cpt1
PC
Cho2
PMME
Opi3
PDME
Opi3
Methylation pathway
B
Exp
PDS
cho
Pct1-GFP
DIC
Pct1-GFP
DIC
cho2Δ opi3Δ
cpt1Δ ept1Δ
-
+
-
+
C
No. of cells (%)
120
100
80
60
40
20
0
INM + Intranuclear
Intranuclear
INM
Cho
- + - + - + - +
Exp
PDS
Exp
PDS
cho2Δopi3Δ
cpt1Δept1Δ

Figure S6. Related to figure 5. Pct1 localization in response to PC levels is independent of the source of PC.

(A) Schematic illustration of PC synthesis through the methylation and Kennedy pathway. The enzymes deleted in the *cho2*Δ*opi3*Δ mutant (blue cross) and in the *cpt1*Δ*ept1*Δ mutant (red cross) are indicated. Cho, choline; PC, phosphatidylcholine; PE, phosphatidylethanolamine; P-cho, Phosphocholine; CDP-Cho, CDP-choline; Etn, ethanolamine; P-Etn, Phosphoethanolamine; CDP-Etn, CDP-ethanolamine; PMME, phosphatidyl-N-mono- methylethanolamine; PDME, phosphatidyl-N,N dimethyl ethanolamine.

(B) Pct1-GFP was expressed in a *cho2*Δ*opi3*Δ mutant (defective in the methylation pathway) or a *cpt1*Δ*ept1*Δ mutant (defective in the CDP-choline pathway). Cells were grown to the indicated growth phase with (+) or without (-) excess choline and imaged as described in Methods. DIC images are shown for context. Scale bars, 5 µm.

(C) Quantification of the Pct1-GFP localization shown in (B). Data are mean ± SD based on random fields from three independent experiments. ~100 cells were counted at each time point in each independent experiment.

Fig. S7

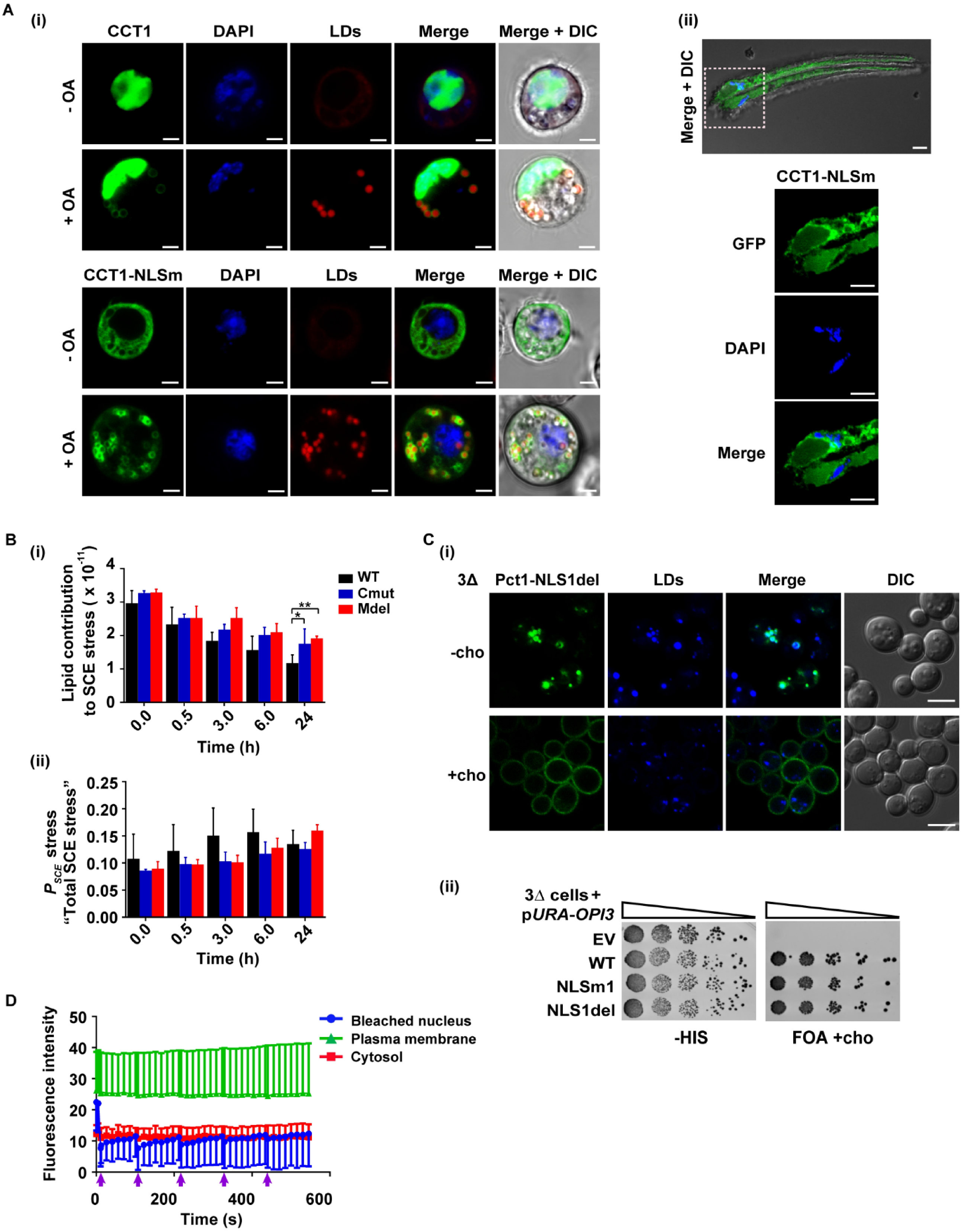
A
(i)
CCT1
DAPI
LDs
Merge
Merge + DIC
- OA
+ OA
CCT1-NLSm
DAPI
LDs
Merge
Merge + DIC
- OA
+ OA
(ii)
Merge + DIC
CCT1-NLSm
GFP
DAPI
Merge
B
(i)
Lipid contribution to SCE stress (x 10^-11)
WT
Cmut
Mdel
Time (h)
0.0
0.5
3.0
6.0
24
(ii)
P_SCE stress "Total SCE stress"
Time (h)
C
(i)
3Δ
Pct1-NLS1del
LDs
Merge
DIC
-cho
+cho
(ii)
3Δ cells + pURA-OPI3
EV
WT
NLSm1
NLS1del
-HIS
FOA +cho
D
Fluorescence intensity
Bleached nucleus
Plasma membrane
Cytosol
Time (s)

Figure S7. Related to figure 6 and 7. Abrogation of the predicted NLS of CCT1 and Pct1 prevents nuclear localization.

(A) The nuclear targeting signal in CCT1 was mutated to alanine residues (^{34}RKR^{35} → AAA) in the CCT1-NLSm mutant. (i) *Drosophila* S2 cells expressing C-terminal GFP-tagged wild type CCT1 (CCT1) or CCT1-NLS mutant (CCT1-NLSm) were grown with (+OA) or without (-OA) oleic acid for 24 hours. Live cell imaging in cells stained for lipid droplet (LDs) and nuclear (DAPI) visualization was performed as described in Methods. The merged fluorescent channels are shown with DIC (Merge + DIC) for context. Scale bars, 2.5 µm. (ii) GFP-tagged CCT1-NLSm was transgenically expressed in *D. melanogaster* eyes under the *Rhodopsin* (*Rh1*) promoter. Individual ommatidia were isolated and imaged as described in methods. All panels are zoomed in images from the boxed region in the 'merge + DIC' panel. Scale bars, 10 µm.

(B) 3Δ yeast cells expressing either WT Pct1-GFP or mutant Cmut- and Mdel-GFP were grown for 24 h followed by addition of 1 mM choline. Cells were collected before (0.0 h) and after choline supplementation at the indicated time points for lipidomics analyses and the (i) lipid-driven and (ii) total SCE stress in the cells was calculated as described in Methods. These estimates correspond to lipidomics data depicted in fig. S5E. Data are shown as mean ± SD from independent repeats (n=8 for WT; n=3 each for Cmut and Mdel Pct1). *P <0.05, **P <0.01 at 24 h using one-way ANOVA with Bonferroni multiple comparisons.

(C) The NLS1 region (amino acids 60-66) of Pct1 was deleted in the Pct1-NLS1del-GFP mutant and expressed in 3Δ cells. (i) Cells were grown with (+) or without (-) choline for 24 hours and stained to visualize lipid droplets (LDs), and imaged as described in Methods. DIC images are shown for context. Scale bar, 5 µm. (ii) The cells were grown to exponential phase and serial dilutions of liquid cultures were spotted onto plates lacking histidine (-HIS) or supplemented with FOA and choline (FOA + cho).

(D) *pct1*Δ cells expressing Pct1-NLSm1-GFP were repeatedly photobleached in the nucleus and there was no loss of GFP fluorescence at the plasma membrane, indicating little or no trafficking of Pct1-

NLSm1-GFP from the PM to the nucleus. Data are mean ± SD from three independent experiments (5-8 cells each). The GFP fluorescence intensities reported here were corrected for loss of fluorescence during imaging as described in Methods. Arrows on X-axis indicate time-points of each bleaching event.
